# Adverse event profile of fondaparinux sodium: a disproportionality analysis based on FAERS, JADER, and VigiAccess databases

**DOI:** 10.3389/fmed.2026.1731378

**Published:** 2026-02-05

**Authors:** Wei Jin, Jianwei Pan, Lin Yu, Menglu Zhu

**Affiliations:** 1 Department of Pharmacy, The Fourth Affiliated Hospital of School of Medicine, and International School of Medicine, International Institutes of Medicine, Zhejiang University, Yiwu, China; 2 Department of Pediatrics, The Fourth Affiliated Hospital of School of Medicine, and International School of Medicine, International Institutes of Medicine, Zhejiang University, Yiwu, China

**Keywords:** anticoagulant, disproportionality analysis, FAERS, fondaparinux sodium, JADER, VigiAccess

## Abstract

**Background:**

Fondaparinux sodium, a synthetic pentose sugar, selectively inhibits factor Xa and is mainly used to prevent venous thromboembolism during major lower extremity orthopedic surgeries. It is also utilized for treating unstable angina pectoris and non-ST-segment-elevation myocardial infarction (NSTEMI). Given its extensive clinical application, comprehensive evaluation of its safety profile in clinical practice is essential.

**Methods:**

This pharmacovigilance investigation systematically evaluated the post-marketing safety profile of fondaparinux sodium by leveraging real-world data from the FDA Adverse Event Reporting System (FAERS) database (Q1 2004 to Q3 2024), where fondaparinux sodium was the primary suspected drug. To further validate the robustness and generalizability of our findings, we cross-referenced the identified signals with data from two external sources: the VigiAccess database (Q2 2003 to Q4 2025) and the Japanese Adverse Drug Event Report (JADER) database (Q2 2007 to Q2 2025). Disproportionality analyses employed the reporting odds ratio (ROR), proportional reporting ratio (PRR), Bayesian confidence propagation neural network (BCPNN), and multiple empirical Bayes Gamma–Poisson shrinker (MGPS). Furthermore, sensitivity analyses were conducted to ensure the robustness of the results. Finally, the optimal parametric model was applied to estimate the temporal risk of adverse events (AEs).

**Results:**

In the FAERS database, we identified 5,700 fondaparinux sodium-related AE reports, corresponding to 17,061 unique Preferred Terms (PTs). Time-to-onset analysis (*N* = 2,220) showed an early-peak profile (*β* = 1.2, median: 7 days), with 81.98% of events within 30 days. Disproportionality analysis confirmed positive signals for haemorrhagic complications across all demographics. Subgroup analysis revealed higher bleeding RORs in females, and increased risks of anemia and skin necrosis in the elderly patients(≥ 65 years). Pediatric patients (< 18 years) showed rare but significant hepatotoxicity. Healthcare professionals reported more complex clinical terms, while consumers reported more symptomatic events. Several unbalanced signals, including haematemesis (ROR = 14.52, 95%CI: 11.99–17.58) and skin necrosis (ROR = 27.29, 95%CI: 20.06–37.12) were identified. Sensitivity analyses further confirmed the robustness of these findings, and key signals were cross-referenced with the VigiAccess database and JADER database. The detection of these signals highlights potential safety concerns that warrant further clinical monitoring rather than confirming definitive causality.

**Conclusion:**

This study serves as a hypothesis-generating inquiry into the real-world safety of fondaparinux sodium. While our findings align with established adverse reaction profiles, they also highlight potential novel signals that warrant further investigation. These insights provide valuable reference points for clinical monitoring, particularly in special populations, though further epidemiological studies are needed to verify causality.

## 1 Introduction

Fondaparinux sodium, a synthetic selective inhibitor of activated factor X, is derived from optimizing the natural pentose sequence found in heparin and low-molecular-weight heparin (LMWH). Its pentose structure significantly enhances the affinity for antithrombin (AT) and specifically binds to the activated site of antithrombin III (AT III) through its noncovalent bond so that the activated coagulation factor is rapidly inhibited, thereby reducing the generation of thrombin and the formation of fibrin. Compared with heparin, fondaparinux sodium is more selective for factor Xa and does not interact with platelets or plasma proteins. As a result, it rarely causes heparin-induced thrombocytopenia (HIT) or other side effects (1). Additionally, fondaparinux sodium has a relatively small molecular weight and long half-life *in vivo* and provides stable and sustained therapeutic effects.

Fondaparinux sodium is primarily utilized for preventing and treating venous thromboembolism (VTE) and acute coronary syndrome (ACS), as well as for preventing VTE during pregnancy and the puerperium (2–5). Owing to its unique mechanism and favourable pharmacokinetics, fondaparinux sodium has shown efficacy and safety in anticoagulation therapy (6–8). Despite the routine inclusion of adverse events such as hemorrhage, hematoma, edema, and anemia in product labeling, the clinical ubiquity of fondaparinux sodium has exposed critical gaps in our current safety framework. A primary concern lies in the inherent limitations of pre-marketing clinical trials; these studies, often characterized by restricted sample sizes and highly homogenized cohorts, struggle to reflect the safety profiles of diverse real-world populations, particularly pregnant women and patients with renal impairment, whose physiological responses to prolonged therapy remain underrepresented. Furthermore, because much of the existing evidence is derived from single-center observations or constrained case series, there is a distinct lack of the large-scale, real-world data necessary for robust clinical decision-making (9, 10). These vulnerabilities are compounded by the absence of a specific reversal agent for fondaparinux sodium, a significant therapeutic challenge that intensifies the risks associated with acute complications (11). Collectively, there is a pressing need for systematic pharmacovigilance research to bridge the gap between controlled trial data and complex clinical practice.

FAERS is a public database that collects global adverse event (AE) reports to monitor drug safety and is extensively used in pharmacovigilance research (12, 13). As a global cornerstone of pharmacovigilance, FAERS aggregates voluntary reports from a broad spectrum of healthcare professionals and consumers. This database serves as an ideal data source for the present study due to its expansive reach and its high temporal resolution facilitating rapid signal detection. Furthermore, the integration of standardized MedDRA coding allows for robust cross-study comparisons. While traditional clinical trials are inherently constrained by limited sample sizes, truncated observation periods, and stringent inclusion criteria, FAERS effectively bridges these gaps, providing a critical window into the rare or long-term complications that only manifest within the complexity of real-world clinical practice.

The application of disproportionality analysis to FAERS data has become a standard methodology for assessing drug safety, having successfully characterized the risk profiles of various agents ranging from antiarrhythmics to chemotherapeutics (14–17). However, to mitigate the biases inherent in single-source data mining and evaluate the global generalizability of the identified signals, it is essential to cross-reference findings with external global datasets. Therefore, in addition to FAERS, we incorporated data from two complementary sources: VigiAccess and JADER database. VigiBase, the WHO global database managed by the Uppsala Monitoring Centre (UMC), aggregates safety data from over 180 member countries (18, 19) and was accessed via its public interface, VigiAccess (19). This multi-source approach facilitates a robust external validation of the FAERS-detected signals across diverse reporting systems and demographics.

By integrating these multi-source real-world data, this research represents the first large-scale, pan-population analysis of fondaparinux sodium across multiple indications. We aim to evaluate established adverse event patterns while exploring potential novel safety signals across specific clinical contexts. These findings offer immediate translational value: they establish a foundation for risk stratification in vulnerable populations and provide evidence to optimize discontinuation windows for perioperative bleeding management. Ultimately, these insights support regulatory bodies in refining risk-control strategies and advancing a dynamic pharmacovigilance framework rooted in real-world evidence.

## 2 Methods and materials

### 2.1 Research methodology and data origins

**Figure 1:**
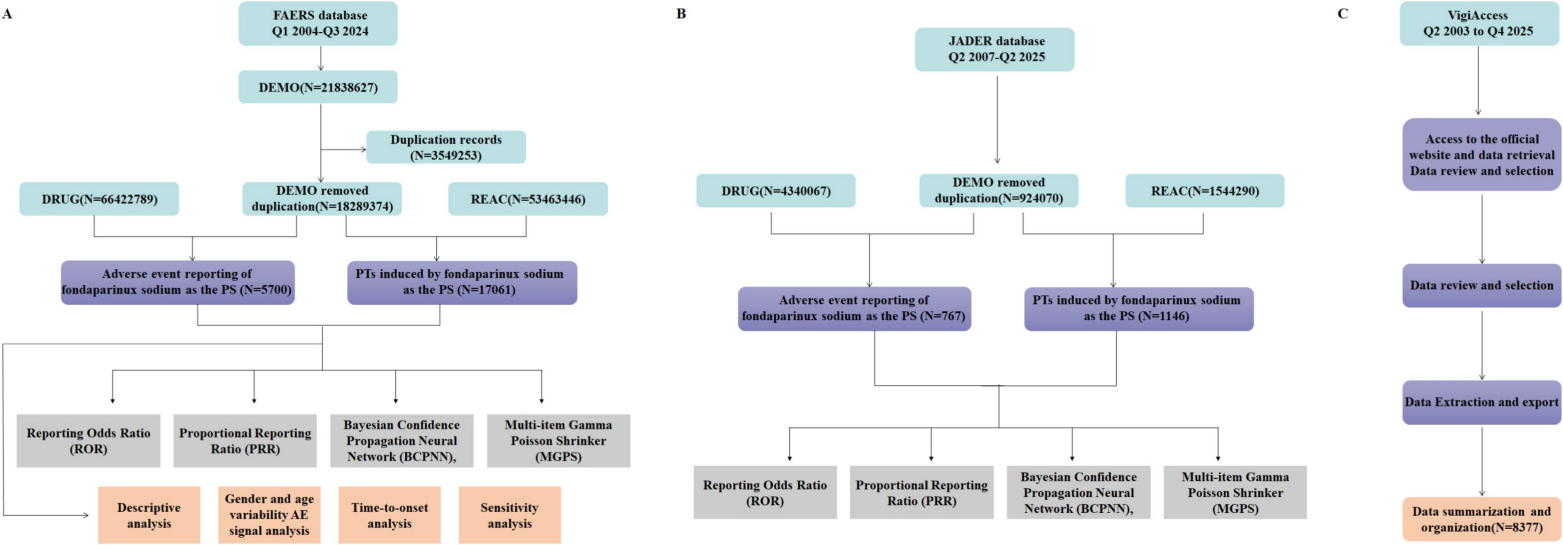
Flowchart of data selection and analysis for fondaparinux sodium-related adverse events in the FAERS database, JADER database, and VigiAccess database. **(A)** Data identification, extraction, and processing in FAERS. **(B)** Data identification, extraction, and processing in JADER. **(C)** Data identification, extraction, and processing in VigiAccess. Data were extracted from the FAERS database, including demographic information (DEMO), drug information (DRUG), and adverse event records (REAC). After deduplication, reports and PTs in which fondaparinux sodium was designated as the PS were included for analysis. Subsequent analyses included signal detection, descriptive analysis, gender and age variability AE signal analysis, time-to-onset analysis, and sensitivity analysis.

This retrospective observational study investigates the association between fondaparinux sodium and clinical adverse effects (AEs) using real-world data from three major pharmacovigilance platforms: the FAERS database, VigiAccess, and JADER database. FAERS is a crucial international database for collecting post-marketing safety data on pharmaceuticals and plays a key role in pharmacovigilance by monitoring safety signals and assessing drug–adverse event relationships. This study analyzed adverse event reports from the FAERS database spanning from Q1 2004 to Q3 2024, specifically focusing on cases where fondaparinux sodium was identified as the Primary Suspect (PS). To further validate the robustness and generalizability of these findings, we additionally queried two external datasets: the VigiAccess database (the public interface of VigiBase, up to Q4 2025) and the JADER database (spanning from Q2 2007 to Q2 2025). Data were extracted from publicly available quarterly files and systematically processed using Excel 2021 and R (version 4.3.0). The comprehensive data cleaning and analysis workflow, designed to extract and validate key safety information, is illustrated in Figure 1.

### 2.2 Data extraction

#### 2.2.1 Data source and processing

The FAERS database architecture consists of seven relational datasets: DEMO (demographic and administrative metadata), DRUG (drug data), REAC (adverse reaction data), OUTC (clinical outcomes), RPSR (reporting sources), INDI (indication details) and THER (drug therapy dates). These files are interconnected via the PRIMARYID variable, which serves as the unique record identifier. To ensure data integrity and mitigate analytical bias, we implemented a rigorous de-duplication protocol aligned with FDA guidelines (20). We selected the latest FDA_DT (FDA date) for matching CASE IDs (case identification numbers) when both CASE IDs and FDA_DT were identical and the higher PRIMARYID was selected.

#### 2.2.2 Signal detection and cohort selection

Safety signals were standardized using the Medical Dictionary for Regulatory Activities (MedDRA, version 27.1), where AEs were coded by Preferred Terms (PT) and categorized by System Organ Classes (SOC). To refine the clinical relevance of our cohort, the analysis was restricted to reports designating fondaparinux (including generic and brand names such as Fondaparinux Sodium, Arixtra, and Quixidar) as the Primary Suspect (PS) medication. Consistent with standardized signal detection practices, Secondary Suspect (SS) and Concomitant (C) medications were excluded. Furthermore, to address potential indication bias—where a pre-existing medical condition is incorrectly flagged as a drug reaction—we proactively excluded any PTs directly related to the drug’s therapeutic indications. Finally, to ensure data quality, reports missing essential demographic variables, such as age or sex, were removed from the analytical dataset.

#### 2.2.3 External validation sources

To validate the robustness of the FAERS findings, we cross-referenced signals with two external databases. First, global aggregate safety data were retrieved from the VigiAccess database,^1^ covering cumulative reports across diverse demographics and geographic regions. Second, we utilized the Japanese Adverse Drug Event Report (JADER) database, managed by the Pharmaceuticals and Medical Devices Agency (PMDA). The JADER database, which collects adverse drug event (ADE) reports from pharmaceutical companies and medical institutions since 2004, comprises four relational datasets: patient demographics (DEMO), drug information (DRUG), adverse reaction data (REAC), and medical history (HIST). In the JADER database, we performed the search using the corresponding Japanese keywords: ‘フォンダパリヌクスナトリウム’ (Fondaparinux Sodium) and ‘アリクストラ’ (Arixtra).

### 2.3 Statistical analysis

This research report strictly follows the READUS-PV checklist (21, 22), which is now one of the recommended reporting frameworks for pharmacovigilance signal-detection studies. Disproportionality analysis was employed to evaluate drug-adverse event associations by comparing target reporting ratios against database background frequencies via 2 × 2 contingency tables (23, 24). We utilized four complementary algorithms to ensure robust signal detection: the Reporting Odds Ratio (ROR), Proportional Reporting Ratio (PRR), Bayesian Confidence Propagation Neural Network (BCPNN), and Multi-item Gamma Poisson Shrinker (MGPS) (25–28). ROR and PRR for their high sensitivity in large datasets (25, 29), and the BCPNN and MGPS for their respective superior stability in quantifying uncertainty and managing rare events (30, 31). It is worth mentioning that disproportionality analysis is a method for detecting statistical associations between drugs and adverse events based on pharmacovigilance data. It solely indicates correlations at the data level and cannot establish causality. The identification of these positive signals was based on predefined thresholds for each algorithm, which serve as indicators of potential safety associations requiring further clinical validation.

In this study, a signal was considered positive if detected by at least one of the four algorithms to ensure comprehensive surveillance and capture rare events critical to patient safety (32, 33). While we used multi-algorithm cross-validation to strengthen core risk identification and reduce false positives, we did not mandate concurrent positivity across all methods. This strategy ensures that clinically significant risks, particularly emerging or rare events, are not overlooked due to the varying statistical stringencies of individual models.

Detailed 2 × 2 contingency tables and the corresponding mathematical formulas and detection thresholds are provided in Supplementary Tables S1, S2, respectively. Higher metric value indicate a more robust signal association between the drug and the adverse event. Signal Strength for signal detection was defined according to established algorithmic thresholds: ROR: The lower limit of the 95% confidence interval> 1, with at least 3 reported cases; PRR: PRR ≥ 2 and X^2^ ≥ 4, supported by 3 or more reports; BCPNN: The lower limit of the 95% confidence interval for the Information Component (IC_025_) > 0; MGPS: The lower limit of the 95% confidence interval for the Empirical Bayes Geometric Mean (EBGM_05_) > 2.

Data quality control was performed prior to analysis. Reports containing missing, erroneous, or logically inconsistent dates—such as instances where the adverse event predated drug administration—underwent a manual audit. We excluded any records that could not be reliably corrected, and the specific number of excluded cases was documented in the study flow.

The time-to-onset (TTO) was calculated as the interval between the commencement of fondaparinux sodium therapy and the occurrence of the AE. To determine the optimal parametric model for the TTO data, we tested and compared Weibull, Log-logistic, and Log-normal distributions. Goodness-of-fit was evaluated using the Akaike Information Criterion (AIC) and visual inspection of diagnostic plots; the model with the lowest AIC was selected for the final analysis. Furthermore, subgroup analyses were stratified by age (<18, 18–64, and ≥65 years) and sex to identify demographic-specific risk variations. All statistical processing was conducted in R (version 4.3.0), using the PhViD package for disproportionality analysis (ROR, PRR, and IC) and the openEBGM package for EBGM.

To evaluate the robustness of the identified safety signals and minimize potential confounding by common co-medications, a sensitivity analysis was performed. Specifically, ROR values were re-calculated after excluding reports involving prednisone, aspirin, and hydroxychloroquine. A signal was considered robust if it remained statistically significant (lower limit of 95% CI > 1 with at least 3 cases) after these exclusions.

## 3 Results

### 3.1 Clinical characteristics

After removing duplicate records from the FAERS database spanning Q1 2004 to Q3 2024, we identified 5,700 reports where fondaparinux sodium was designated as the PS medication. Standardization of these adverse event reports yielded 17,061 unique PTs associated with fondaparinux sodium therapy. According to data from the VigiAccess database, the earliest recorded adverse events associated with fondaparinux sodium date back to 2003. As of 2025, the WHO has accumulated a cumulative total of 8,376 reports related to fondaparinux sodium ADRs. Meanwhile, our analysis of the JADER database (spanning from Q2 2007 to Q2 2025) identified a total of 767 reports specifically within the Japanese population. Among FAERS database, 34.6% (*N* = 1,975) were reported for male patients, whereas 48.51% (*N* = 2,765) were reported for female patients, indicating a greater proportion of reports for females than males. Notably, this trend of higher reporting frequency in females was consistently observed across both the VigiAccess and JADER databases. In terms of age distribution, the elderly group (≥65 years) constituted the largest demographic in both the FAERS and VigiAccess databases, accounting for 38.26 and 46.45% of the total reports, respectively. Geographically, reporting volumes in both databases followed an identical descending order: Europe, the Americas, Asia, Africa, and Oceania. The clinical characteristics of the reports are summarized in Table 1.

**Table 1:** Clinical characteristics of fondaparinux sodium adverse event reports from the FAERS (Q1 2004-Q3 2024) FAERS, JADER (Q2 2007 to Q2 2025), and VigiAccess database (Q2 2003 to Q4 2025).

Characteristics	FAERS	JADER	VigiAccess
Number of events	5,700	767	8,376
Gender	*n* (%)		
Male	1,975 (34.65%)	223 (29.07%)	3,457 (41.27%)
Female	2,765 (48.51%)	501 (65.32%)	4,307 (51.41%)
Missing	960 (16.84%)	43 (5.61%)	613 (7.32%)
Age	*n* (%)		
<18	28 (0.49%)	N/A	57 (0.68%)
18–64	1,548 (27.16%)	N/A	2,823 (33.70%)
≥65	2,181 (38.26%)	N/A	3,891 (46.45%)
Missing	1,943 (34.09%)	N/A	1,606 (19.17%)
Top 5 reported countries	*n* (%)		
United States	2,022 (35.47%)	N/A	N/A
France	1,586 (27.82%)	N/A	N/A
Japan	420 (7.37%)	767 (100%)	N/A
Ireland	313 (5.49%)	N/A	N/A
Germany	259 (4.54%)	N/A	N/A
Geographical distribution	*n* (%)		
Europe	2,716 (47.65%)	N/A	4,520 (53.96%)
Americas	2,108 (36.98%)	N/A	2,472 (29.51%)
Asia	565 (9.91%)	767 (100%)	1,285 (15.34%)
Unspecified	244 (4.28%)	N/A	N/A
Africa	63 (1.11%)	N/A	89 (1.06%)
Oceania	4 (0.07%)	N/A	11 (0.13%)
Reporter	*n* (%)		
Healthcare professional	2,863 (50.23%)	699 (91.13%)	N/A
Non-healthcare professional	2,677 (46.96%)	64 (8.34%)	N/A
Missing	160 (2.81%)	4 (0.52%)	N/A
Reporting year	*n* (%)		
2025	N/A	2 (0.26%)	281 (3.35%)
2024	40 (0.70%)	1 (0.13%)	237 (2.83%)
2023	78 (1.37%)	6 (0.78%)	259 (3.09%)
2022	104 (1.82%)	2 (0.26%)	264 (3.15%)
2021	102 (1.79%)	1 (0.13%)	291 (3.47%)
2020	129 (2.26%)	2 (0.26%)	266 (3.18%)
2019	129 (2.26%)	2 (0.26%)	406 (4.85%)
2018	120 (2.11%)	N/A	359 (4.29%)
2017	169 (2.96%)	26 (3.39%)	338 (4.04%)
2016	213 (3.74%)	9 (1.17%)	305 (3.64%)
2015	219 (3.84%)	16 (2.09%)	414 (4.94%)
2014	408 (7.16%)	27 (3.52%)	1843 (22.00%)
2013	482 (8.46%)	30 (3.91%)	245 (2.93%)
2012	420 (7.37%)	63 (8.21%)	471 (5.62%)
2011	351 (6.16%)	64 (8.34%)	470 (5.61%)
2010	592 (10.39%)	80 (10.43%)	398 (4.75%)
2009	615 (10.79%)	132 (17.21%)	392 (4.68%)
2008	557 (9.77%)	158 (20.60%)	662 (7.90%)
2007	479 (8.40%)	146 (19.04%)	14 (0.17%)
2006	232 (4.07%)	N/A	60 (0.72%)
2005	77 (1.35%)	N/A	124 (1.48%)
2004	184 (3.23%)	N/A	178 (2.13%)
2003	N/A	N/A	99 (1.18%)

### 3.2 Signal detection associated with fondaparinux sodium

As detailed in Table 2, fondaparinux-related adverse events in the FAERS database involved a broad spectrum of 27 SOCs. Based on report frequency, the three most prominent SOCs were General disorders and administration site conditions (*N* = 2,221, 13.02%), Vascular disorders (*N* = 1,970, 11.55%), and Injury, poisoning and procedural complications (*N* = 1,788, 10.48%). Across the four disproportionativity algorithms, seven SOCs generated at least one positive signal, including Vascular disorders (*N* = 1,970, 11.55%), Blood and lymphatic system disorders (*N* = 1,074, 6.3%), Injury, poisoning and procedural complications (*N* = 1,788, 10.48%), Gastrointestinal disorders (*N* = 1,710, 10.02%), Investigations (*N* = 1,551, 9.09%), Renal and urinary disorders (*N* = 399, 2.34%), and Hepatobiliary disorders (*N* = 184, 1.08%). Among these, the first two SOCs met the positivity criteria for all four methods and exhibited the highest ROR values, while the latter five met the thresholds for the ROR and BCPNN algorithms. Crucially, the robustness of some signals was corroborated by the JADER database analysis (Supplementary Table S3). Consistent with FAERS database, JADER database identified positive signals for Injury, poisoning and procedural complications (*N* = 290, ROR = 10.73, 95% CI: 9.39–12.26), Vascular disorders (*N* = 139, ROR = 5.18, 95% CI: 4.34–6.19), and Gastrointestinal disorders (*N* = 197, ROR = 2.39, 95% CI: 2.05–2.79). Notably, despite the broad involvement of multiple categories, the majority of SOCs yielded negative results in the disproportionativity analysis.

**Table 2:** Signal strength of fondaparinux sodium AEs across SOC in the FAERS database.

System organ class (SOC)	Case numbers	ROR (95%CI)	PRR (95%CI)	*χ* ^2^	EBGM (EBGM05)	IC (IC025)
General disorders and administration site conditions	2,221	0.7 (0.67, 0.73)	0.74 (0.7, 0.78)	242.62	0.74 (0.71)	−0.43 (−0.5)
Vascular disorders*	1,970	5.87* (5.6, 6.16)	5.31* (5.27, 5.35)	7,033.09	5.3* (5.1)	2.41* (2.34)
Injury, poisoning and procedural complications*	1,788	1.13* (1.07, 1.18)	1.11 (1.07, 1.16)	22.41	1.11 (1.07)	0.15* (0.08)
Gastrointestinal disorders*	1,710	1.18* (1.12, 1.24)	1.16 (1.12, 1.21)	42.7	1.16 (1.12)	0.22* (0.14)
Investigations*	1,551	1.5 (1.42, 1.58)	1.45 (1.41, 1.5)	234.73	1.45 (1.39)	0.54 (0.46)
Nervous system disorders	1,510	1.04 (0.98, 1.09)	1.03 (01.08)	1.89	1.03 (0.99)	0.05 (−0.03)
Blood and lymphatic system disorders*	1,074	3.85* (3.62, 4.09)	3.67* (3.61, 3.73)	2,117.44	3.66* (3.48)	1.87* (1.78)
Respiratory, thoracic and mediastinal disorders	863	1.05 (0.98, 1.13)	1.05 (0.99, 1.12)	2.23	1.05 (0.99)	0.07 (−0.03)
Skin and subcutaneous tissue disorders	747	0.8 (0.74, 0.86)	0.81 (0.74, 0.88)	35.96	0.81 (0.76)	−0.31 (−0.42)
Musculoskeletal and connective tissue disorders	696	0.76 (0.7, 0.82)	0.77 (0.69, 0.84)	52.5	0.77 (0.72)	−0.38 (−0.5)
Cardiac disorders	482	1.06 (0.97, 1.16)	1.06 (0.97, 1.14)	1.44	1.06 (0.98)	0.08 (−0.06)
Renal and urinary disorders*	399	1.26* (1.15, 1.4)	1.26 (1.16, 1.36)	21.62	1.26 (1.16)	0.33* (0.19)
Infections and infestations	339	0.36 (0.32, 0.4)	0.37 (0.27, 0.48)	374.92	0.37 (0.34)	−1.42 (−1.58)
Psychiatric disorders	238	0.23 (0.2, 0.26)	0.24 (0.12, 0.37)	597.67	0.24 (0.22)	−2.04 (−2.23)
Neoplasms benign, malignant and unspecified (incl cysts and polyps)	226	0.49 (0.43, 0.56)	0.49 (0.37, 0.62)	119.74	0.49 (0.44)	−1.01 (−1.21)
Product issues	202	0.75 (0.65, 0.86)	0.75 (0.61, 0.89)	17.27	0.75 (0.67)	−0.42 (0.62)
Surgical and medical procedures	198	0.85 (0.74, 0.98)	0.85 (0.71, 0.99)	5.19	0.85 (0.76)	−0.23 (−0.44)
Hepatobiliary disorders*	184	1.17* (1.01, 1.36)	1.17 (1.03, 1.31)	4.61	1.17 (1.04)	0.23* (0.01)
Metabolism and nutrition disorders	173	0.46 (0.4, 0.54)	0.47 (0.32, 0.62)	106.93	0.47 (0.41)	−1.1 (−1.32)
Eye disorders	134	0.38 (0.32, 0.45)	0.39 (0.22, 0.56)	131.68	0.39 (0.34)	−1.36 (−1.61)
Pregnancy, puerperium and perinatal conditions	87	1.18 (0.95, 1.45)	1.17 (0.97, 1.38)	2.27	1.17 (0.98)	0.23 (−0.08)
Immune system disorders	76	0.4 (0.32, 0.5)	0.4 (0.17, 0.62)	70.01	0.4 (0.33)	−1.33 (−1.66)
Reproductive system and breast disorders	72	0.51 (0.4, 0.64)	0.51 (0.28, 0.74)	34.46	0.51 (0.42)	−0.98 (−1.31)
Social circumstances	40	0.54 (0.39, 0.73)	0.54 (0.23, 0.85)	16.11	0.54 (0.41)	−0.9 (−1.35)
Congenital, familial and genetic disorders	29	0.55 (0.38, 0.79)	0.55 (0.19, 0.92)	10.54	0.55 (0.41)	−0.86 (−1.38)
Ear and labyrinth disorders	27	0.36 (0.25, 0.53)	0.36 (−0.01, 0.74)	30.18	0.36 (0.27)	−1.46 (−2)
Endocrine disorders	25	0.57 (0.39, 0.85)	0.57 (0.18, 0.96)	8.02	0.57 (0.41)	−0.81 (−1.37)

Asterisks (*) indicate positive signals in algorithm; ROR, reporting odds ratio; PRR, proportional reporting ratio; EBGM, empirical Bayesian geometric mean; EBGM05, the lower limit of the 95% CI of EBGM; *χ*^2^, chi-squared; IC, information component; IC025, the lower limit of the 95% CI of the IC; CI, confidence interval; AEs, adverse events.

**Figure 2:**
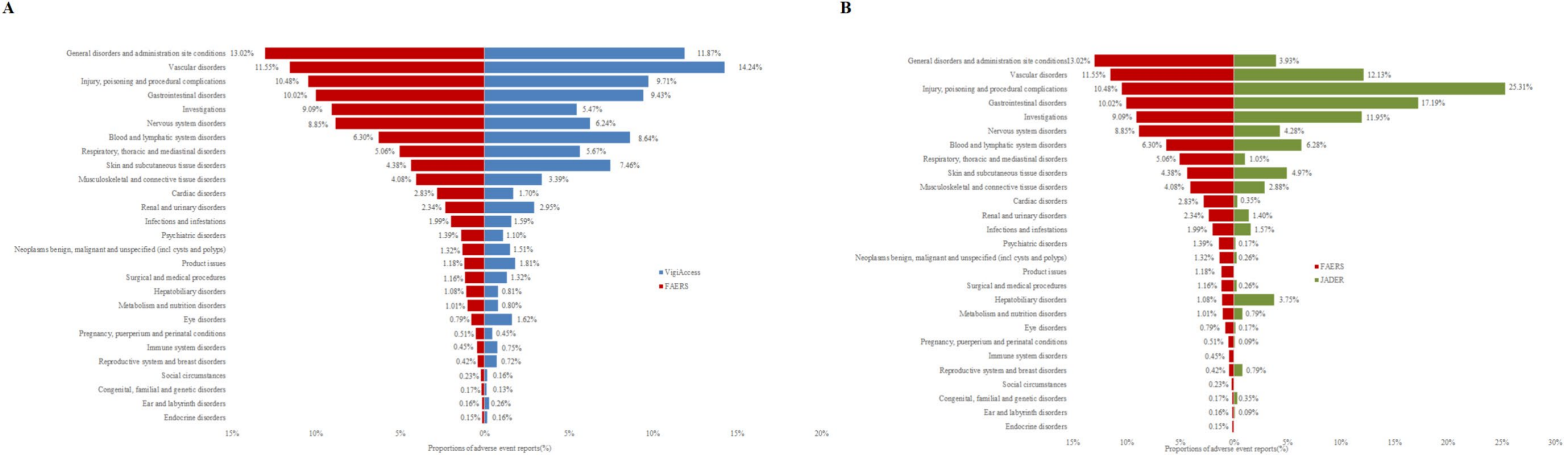
Comparison of AE distribution across SOCs between FAERS and external validation databases. **(A)** Proportions of AEs by system organ class for fondaparinux sodium based on data from FAERS and VigiAccess. **(B)** Proportions of AEs by system organ class for fondaparinux sodium based on data from FAERS and JADER. The *x*-axis represents the proportion of adverse event reports (%), and the *y*-axis lists different SOC categories. SOC, System Organ Class; AE, adverse event.

**Figure 3:**
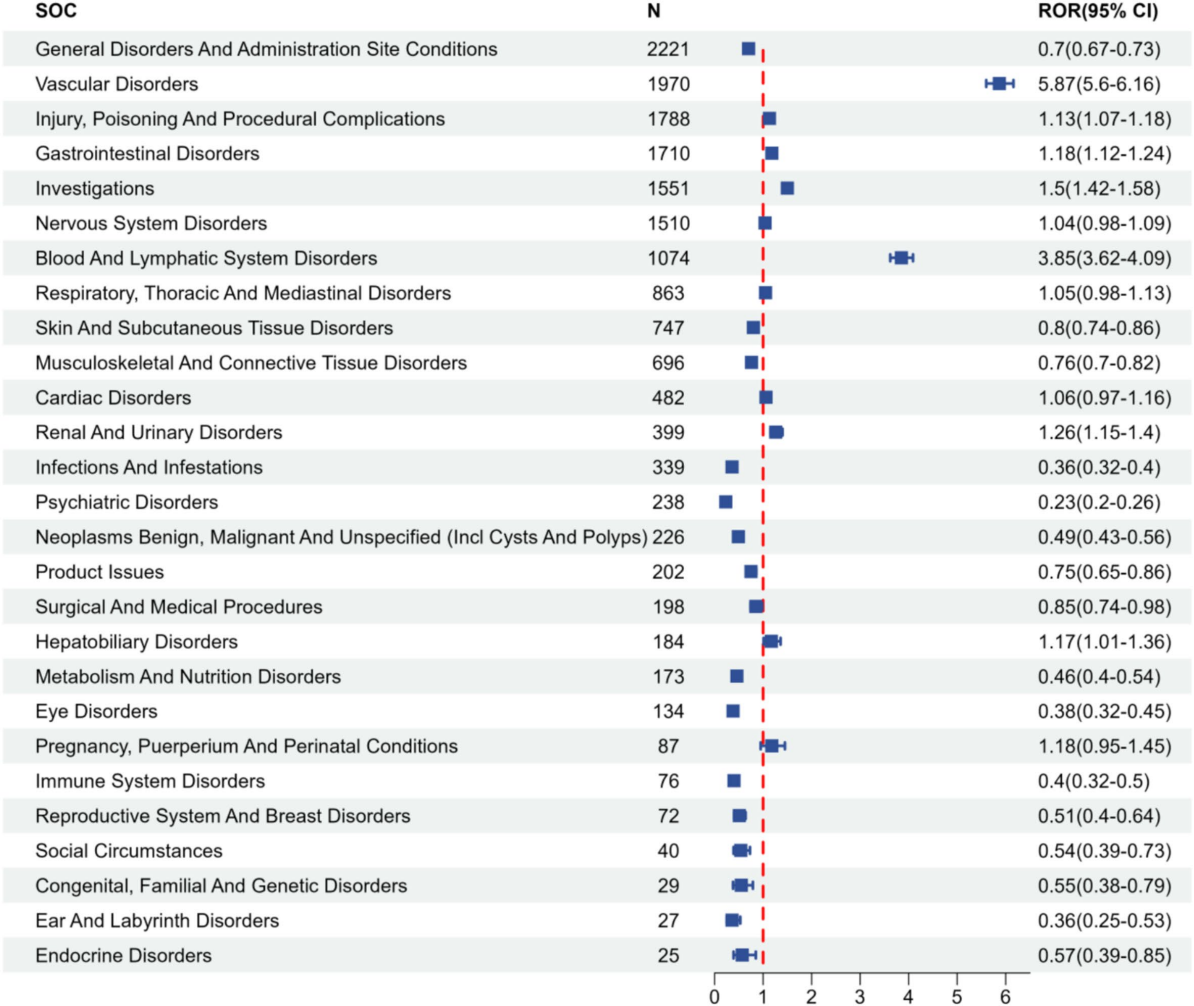
Forest plot of signal strength for fondaparinux sodium-related adverse events at the SOC level in the FAERS database. The *x*-axis shows ROR and its 95% CI, and the *y*-axis lists different SOC categories, ranked by N. Data are derived from Table 2. SOC, System Organ Class; N, number of reports; ROR, Reporting Odds Ratio; CI, Confidence Interval.

The distribution of AEs across System Organ Classes (SOCs) was compared between FAERS and the two external validation datasets, VigiAccess and JADER. Presented in the form of butterfly plots, these comparative analyses reveal the overall concordance as well as region-specific variations in the safety characteristics of fondaparinux sodium (Figure 2). Furthermore, the associated signal strengths within the FAERS database, characterized by ROR values and 95% confidence intervals, are illustrated across various SOC in the FAERS database in Figure 3.

### 3.3 Distribution of adverse events at the PT level

**Figure 4:**
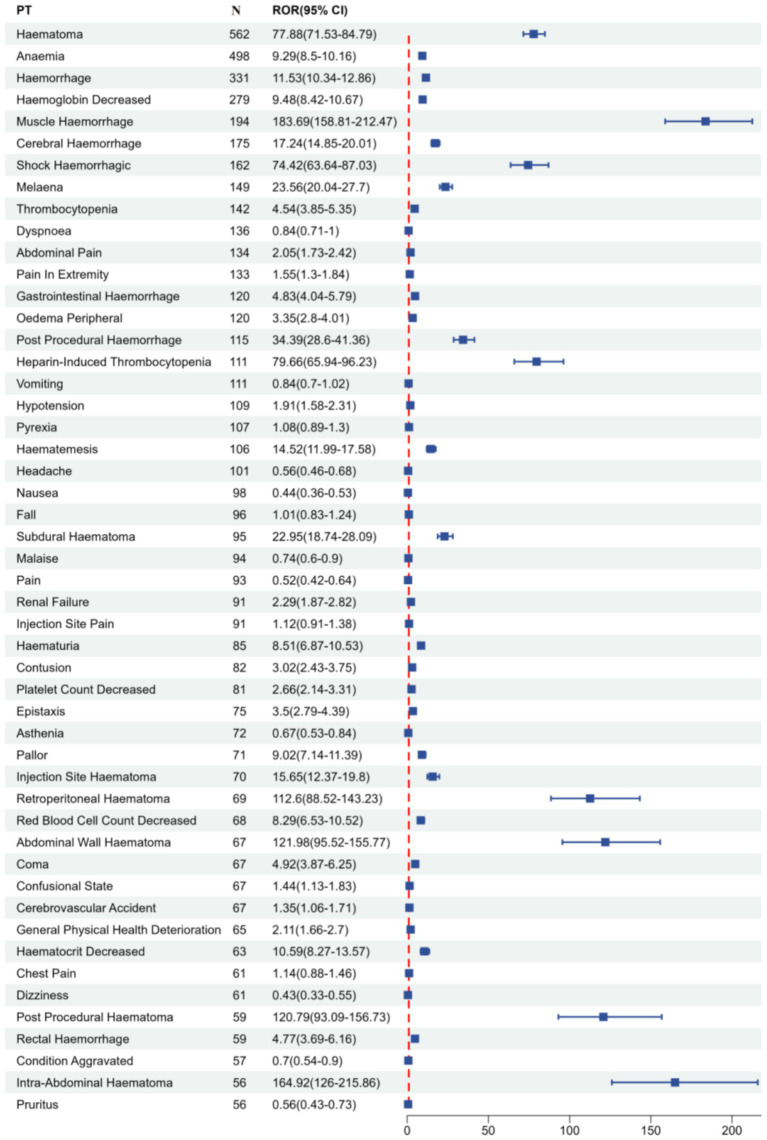
Forest plot of the top 50 signal strength for fondaparinux sodium-related adverse events in the FAERS database at the PT level. The *x*-axis shows ROR and its 95% CI, and the *y*-axis lists different PTs. PT, Preferred Term; ROR, Reporting Odds Ratio; CI, Confidence Interval; The forest plots display the top 50 most frequently reported PTs, including those that did not meet the predefined disproportionality thresholds, providing a complete overview of the reported safety profile.

**Figure 5:**
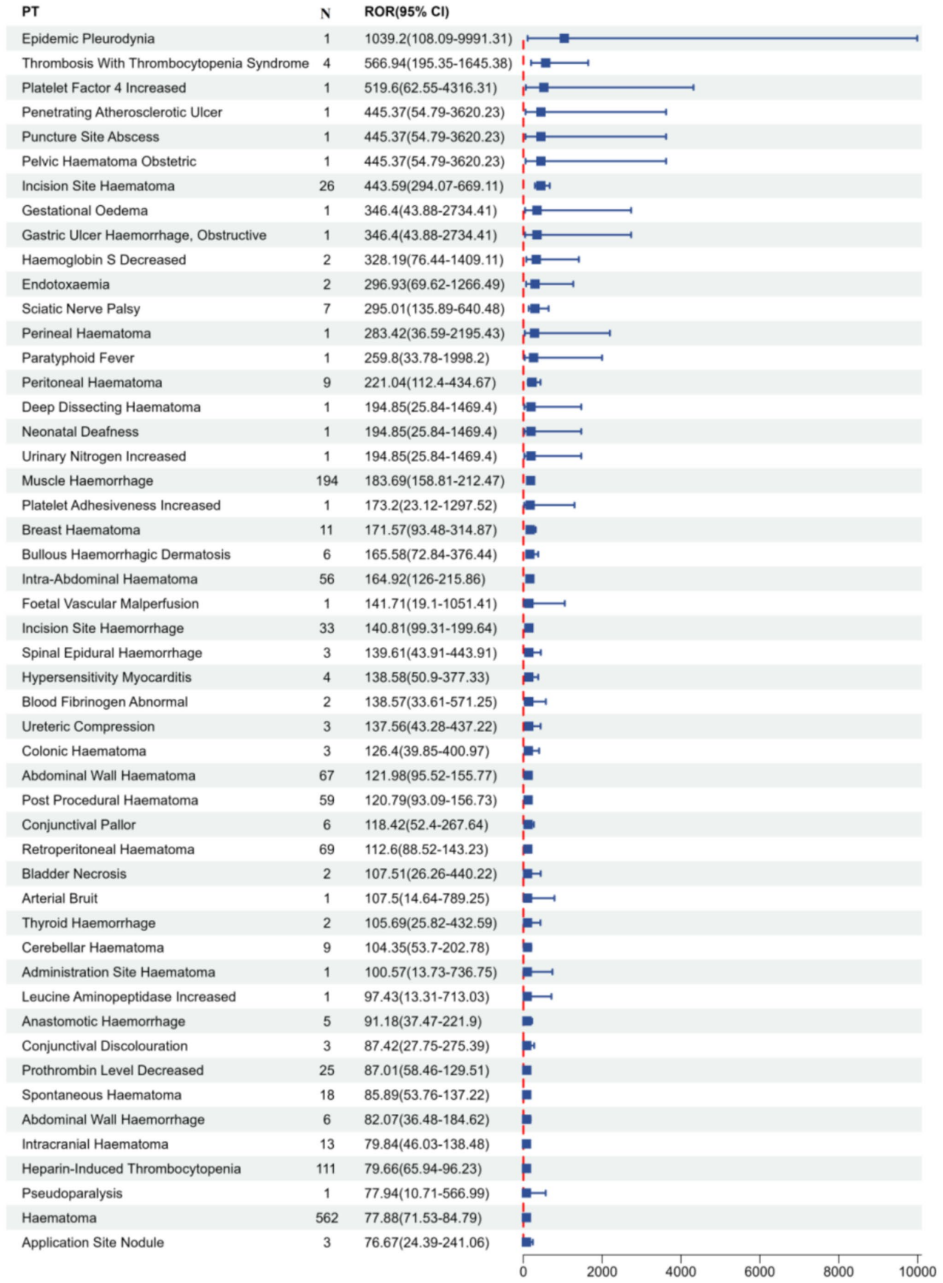
Forest plot of the top 50 ROR for fondaparinux sodium-related adverse events in the FAERS database at the PT level. The *x*-axis shows ROR and its 95% CI, and the *y*-axis lists different PTs. PT, preferred term; N, number of reports; ROR, reporting odds ratio; CI, confidence interval. This figure is intended for comparative analysis of ROR magnitude rather than a list of confirmed positive signals.

This study employed four algorithms at the PT level to analyse adverse drug reactions and assess adherence to screening criteria for PT acquisition. Fondaparinux sodium-related adverse events in the FAERS database were prioritized in descending order according to their report frequencies and ROR values. Table 3 summarizes the clinical profiles of the top 50 PTs by reporting frequency, which are further visualized in Figure 4. The top three PTs in terms of adverse drug event (ADE) report frequency were haematoma (562 cases), anaemia (498 cases) and haemorrhage (331 cases). A forest plot of the top 50 PTs according to ROR ranking is presented in Figure 5, highlighting the comparative signal intensities. The top three PTs according to the ROR values were epidemic pleurodynia (ROR = 1,039.2, 95%CI: 108.09–9,991.31), thrombosis with thrombocytopenia syndrome (ROR = 566.94, 95%CI: 195.35–1645.38) and platelet factor 4 increased (ROR = 519.6, 95%CI: 62.55–4,316.31). To address the potential for statistical instability in sparse data, signals with fewer than 3 reported cases or those exhibiting disproportionately wide 95% confidence intervals were interpreted with caution. These findings were characterized as preliminary signals rather than robust associations, following the methodological framework (26).

**Table 3:** Top 50 most frequently reported adverse events at the PT level for fondaparinux sodium in the FAERS database.

PT	Case numbers	ROR (95%CI)	PRR (95%CI)	*χ* ^2^	EBGM (EBGM05)	IC (IC025)
Haematoma*	562	77.88* (71.53, 84.79)	75.34* (75.26, 75.43)	40,272.33	73.59* (68.54)	6.2* (6.08)
Anaemia*	498	9.29* (8.5, 10.16)	9.05* (8.96, 9.13)	3,566.04	9.02* (8.37)	3.17* (3.04)
Haemorrhage*	331	11.53* (10.34, 12.86)	11.32* (11.22, 11.43)	3,109.51	11.29* (10.3)	3.5* (3.34)
Haemoglobin decreased*	279	9.48* (8.42, 10.67)	9.34* (9.22, 9.46)	2,075.03	9.31* (8.44)	3.22* (3.05)
Muscle haemorrhage*	194	183.69* (158.81, 212.47)	181.62* (181.47, 181.76)	32,930.34	171.67* (151.99)	7.42* (7.21)
Cerebral haemorrhage*	175	17.24* (14.85, 20.01)	17.07* (16.92, 17.22)	2,634.63	16.98* (14.99)	4.09* (3.87)
Shock haemorrhagic*	162	74.42* (63.64, 87.03)	73.73* (73.57, 73.88)	11,354.79	72.05* (63.2)	6.17* (5.94)
Melaena*	149	23.56* (20.04, 27.7)	23.36* (23.2, 23.53)	3,167.22	23.2* (20.26)	4.54* (4.3)
Thrombocytopenia*	142	4.54* (3.85, 5.35)	4.51* (4.34, 4.67)	387.87	4.5* (3.92)	2.17* (1.93)
Dyspnoea	136	0.84 (0.71, 1)	0.84 (0.68, 1.01)	3.95	0.84 (0.73)	−0.24 (−0.49)
Abdominal pain*	134	2.05*(1.73, 2.42)	2.04*(1.87, 2.21)	70.95	2.04 (1.77)	1.03* (0.78)
Pain in extremity*	133	1.55* (1.3, 1.84)	1.54 (1.37, 1.71)	25.57	1.54 (1.34)	0.63* (0.38)
Gastrointestinal haemorrhage*	120	4.83* (4.04, 5.79)	4.81* (4.63, 4.99)	361.77	4.8* (4.13)	2.26* (2)
Oedema peripheral*	120	3.35* (2.8, 4.01)	3.33* (3.16, 3.51)	196.29	3.33* (2.87)	1.74* (1.47)
Post procedural haemorrhage*	115	34.39* (28.6, 41.36)	34.17* (33.99, 34.35)	3,663.41	33.81* (28.98)	5.08* (4.81)
Heparin-induced thrombocytopenia*	111	79.66* (65.94, 96.23)	79.15* (78.96, 79.34)	8,353.44	77.21* (65.92)	6.27* (5.99)
Vomiting	111	0.84 (0.7, 1.02)	0.84 (0.66, 1.03)	3.25	0.84 (0.72)	−0.25 (−0.52)
Hypotension*	109	1.91* (1.58, 2.31)	1.9 (1.72, 2.09)	46.88	1.9 (1.63)	0.93* (0.65)
Pyrexia	107	1.08 (0.89, 1.3)	1.08 (0.89, 1.27)	0.58	1.08 (0.92)	0.11 (−0.17)
Haematemesis*	106	14.52* (11.99, 17.58)	14.43* (14.24, 14.62)	1,319.52	14.37*(12.24)	3.84* (3.56)
Headache	101	0.56 (0.46, 0.68)	0.56 (0.37, 0.76)	34.37	0.56 (0.48)	−0.83 (−1.11)
Nausea	98	0.44 (0.36, 0.53)	0.44 (0.24, 0.64)	71.12	0.44 (0.37)	−1.19 (−1.48)
Fall	96	1.01 (0.83, 1.24)	1.01 (0.81, 1.21)	0.01	1.01 (0.86)	0.02 (−0.28)
Subdural haematoma*	95	22.95* (18.74, 28.09)	22.82* (22.62, 23.02)	1,968.45	22.66* (19.13)	4.5* (4.21)
Malaise	94	0.74 (0.6, 0.9)	0.74 (0.54, 0.94)	8.83	0.74 (0.62)	−0.44 (−0.74)
Pain	93	0.52 (0.42, 0.64)	0.52 (0.32, 0.72)	41.32	0.52 (0.44)	−0.94 (−1.24)
Renal failure*	91	2.29*(1.87, 2.82)	2.29*(2.08, 2.49)	65.94	2.29 (1.92)	1.19* (0.89)
Injection site pain	91	1.12 (0.91, 1.38)	1.12 (0.92, 1.33)	1.22	1.12 (0.94)	0.17 (−0.14)
Haematuria*	85	8.51* (6.87, 10.53)	8.47* (8.26, 8.69)	559.04	8.45* (7.07)	3.08* (2.77)
Contusion*	82	3.02* (2.43, 3.75)	3.01* (2.79, 3.22)	109.87	3* (2.51)	1.59* (1.27)
Platelet count decreased*	81	2.66* (2.14, 3.31)	2.66* (2.44, 2.87)	83.72	2.65* (2.21)	1.41* (1.09)
Epistaxis*	75	3.5* (2.79, 4.39)	3.49* (3.26, 3.72)	133.31	3.49* (2.88)	1.8*(1.47)
Asthenia	72	0.67 (0.53, 0.84)	0.67 (0.44, 0.9)	11.7	0.67 (0.55)	−0.58 (−0.91)
Pallor*	71	9.02* (7.14, 11.39)	8.99* (8.76, 9.22)	502.88	8.97* (7.37)	3.16* (2.82)
Injection site haematoma*	70	15.65* (12.37, 19.8)	15.59* (15.35, 15.82)	951.16	15.52* (12.74)	3.96* (3.61)
Retroperitoneal haematoma*	69	112.6* (88.52, 143.23)	112.15* (111.91, 112.39)	7,337.24	108.29* (88.54)	6.76* (6.41)
Red blood cell count decreased*	68	8.29* (6.53, 10.52)	8.26* (8.02, 8.5)	433.02	8.24* (6.75)	3.04* (2.69)
Abdominal wall haematoma*	67	121.98* (95.52, 155.77)	121.51* (121.26, 121.75)	7,707.26	116.98* (95.34)	6.87* (6.51)
Coma*	67	4.92* (3.87, 6.25)	4.9* (4.66, 5.14)	207.81	4.89* (4)	2.29* (1.94)
Confusional state*	67	1.44* (1.13, 1.83)	1.44 (1.2, 1.68)	9.01	1.44 (1.18)	0.53* (0.17)
Cerebrovascular accident*	67	1.35* (1.06, 1.71)	1.35 (1.11, 1.59)	6	1.35 (1.1)	0.43* (0.08)
General physical health deterioration*	65	2.11* (1.66, 2.7)	2.11* (1.87, 2.35)	38.02	2.11 (1.72)	1.08* (0.72)
Haematocrit decreased*	63	10.59* (8.27, 13.57)	10.56* (10.31, 10.8)	543.39	10.52* (8.55)	3.4* (3.03)
Chest pain	61	1.14 (0.88, 1.46)	1.13 (0.88, 1.39)	0.98	1.13 (0.92)	0.18 (−0.19)
Dizziness	61	0.43 (0.33, 0.55)	0.43 (0.18, 0.68)	46.55	0.43 (0.35)	−1.22 (−1.59)
Post procedural haematoma*	59	120.79* (93.09, 156.73)	120.37* (120.11, 120.63)	6,724.96	115.93* (93.23)	6.86* (6.48)
Rectal haemorrhage*	59	4.77* (3.69, 6.16)	4.75* (4.5, 5.01)	174.74	4.75* (3.83)	2.25* (1.87)
Condition aggravated	57	0.7 (0.54, 0.9)	0.7 (0.44, 0.96)	7.54	0.7 (0.56)	−0.52 (−0.9)
Intra-abdominal haematoma*	56	164.92* (126, 215.86)	164.38* (164.12, 164.65)	8,638.51	156.2* (124.7)	7.29* (6.89)
Pruritus	56	0.56 (0.43, 0.73)	0.56 (0.3, 0.82)	19.28	0.56 (0.45)	−0.83 (−1.22)

Asterisks (*) indicate positive signals in algorithm; Bolded terms represent potential novel signals in top 50 frequency of adverse events at the PT level for fondaparinux sodium; ROR, reporting odds ratio; PRR, proportional reporting ratio; EBGM, empirical Bayesian geometric mean; PT, preferred term; IC025, the lower limit of the 95% credibility interval for the Information Component (IC); EBGM05, the lower limit of the 95% confidence interval for the Empirical Bayes Geometric Mean (EBGM); *χ*^2^, chi-squared.

**Figure 6:**
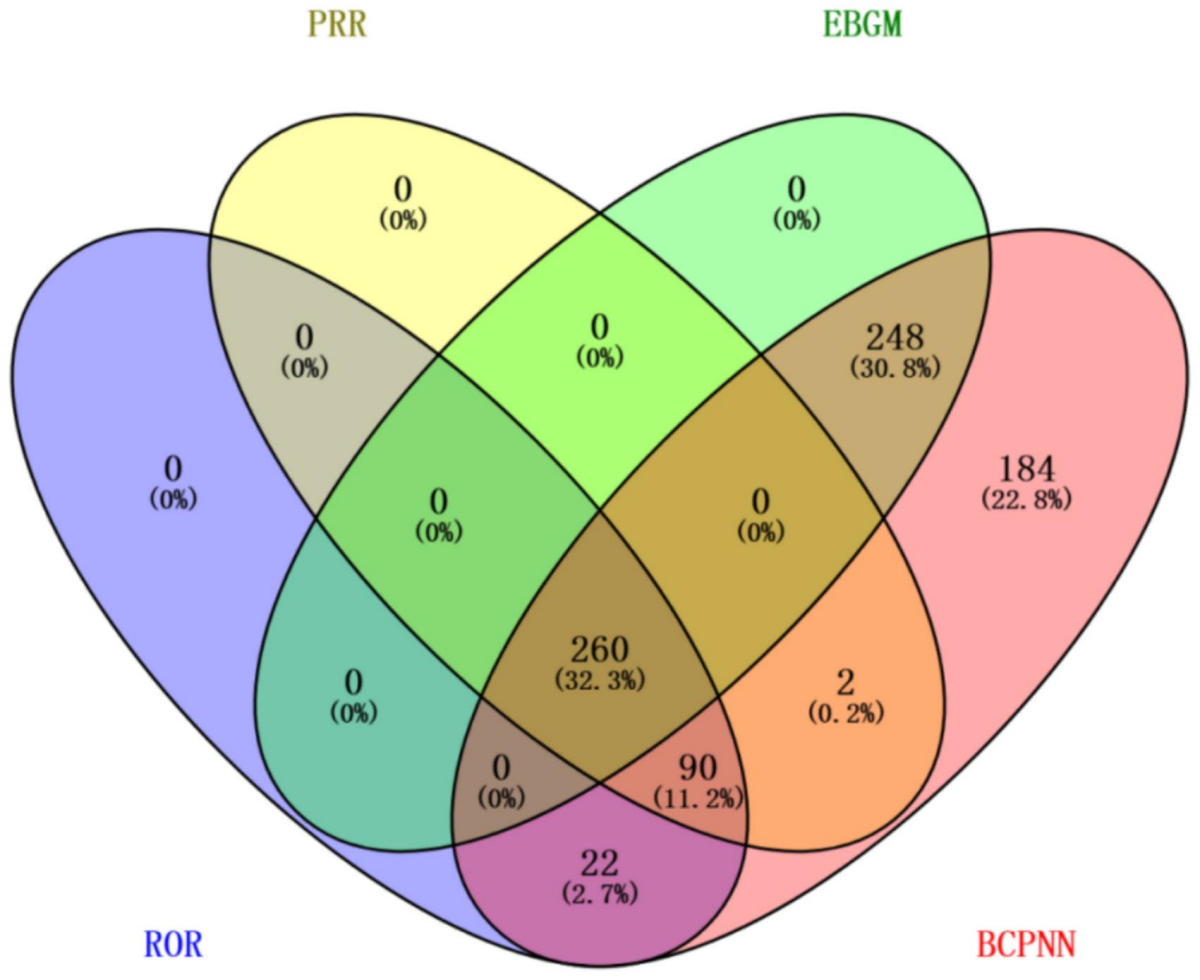
Sensitivity analysis of disproportionality signals based on methodological concordance. The Venn diagram illustrates the overlap of positive signals detected by four different disproportionality algorithms (ROR, PRR, EBGM, and BCPNN) in the FAERS database at the PT level. A total of 260 AEs were consistently identified as positive signals by all four algorithms. PT, Preferred Term; AE, Adverse Event; ROR, Reporting Odds Ratio; PRR, Proportional Reporting Ratio; EBGM, Empirical Bayes Geometric Mean; BCPNN, Bayesian Confidence Propagation Neural Network.

In the FAERS database, the PRR method identified 352 PTs, the ROR method identified 372 PTs, the EBGM method identified 508 PTs, and the BCPNN method identified 806 PTs. The Venn diagram (Figure 6) illustrates the overlap and distribution of signals identified by 1, 2, 3, or all 4 methods. The results showed that 260 robust signals (32.3%) were consistently identified as positive signals by all four algorithms. Among the adverse events related to fondaparinux sodium, known adverse reactions, including bleeding, hematoma, HIT and anemia were confirmed. Additionally, unlabelled adverse reactions in the product information, including haematemesis (106 cases, 0.62%), renal failure (91 cases, 0.53%), skin necrosis (41 cases, 0.24%), anuria (20 cases, 0.12%), conjunctival discolouration (3 cases, 0.02%), aphasia (35 cases, 0.21%), and coma (67 cases, 0.39%) were identified. Haematemesis, renal failure and coma is highlighted in bold in Table 3 as an unlabelled event. Other potential unlabelled signals, though not included in the Top 50 list due to lower reporting frequencies, met the positive criteria for all four algorithms. These 260 robust signals are documented in detail in Supplementary Table S4. In parallel, external validation using the JADER database revealed 68 PTs that met the positivity criteria for at least one of the four disproportionality methods (Supplementary Table S5). Among these, several key signals identified in the FAERS database were consistently validated in JADER, including established reactions such as bleeding, hematoma, HIT and anemia, as well as critical unlabelled events like haematemesis and skin necrosis. While not all 68 signals from JADER overlapped with the FAERS findings due to region-specific reporting variations, the concordance of these clinically significant adverse events across both databases provides additional support for the robustness of our primary findings. Furthermore, several potential signals identified in FAERS were also recorded in VigiAccess, including Haematemesis (102 cases, 0.63%), Coma (20 cases, 0.12%), Aphasia (16 cases, 0.10%), Renal failure (39 cases, 0.24%), Anuria (6 cases, 0.04%), and Skin necrosis (21 cases, 0.13%).

### 3.4 Subgroup analysis

Subgroup analysis revealed that among the top 50 positive signals, anaemia, haematoma, haemorrhage, muscle haemorrhage, and HIT exhibited robust signals across both sexes. These findings align with the biological profile of fondaparinux sodium as a selective Factor Xa inhibitor. The IC_025_ values for these core PTs remained consistently above 0 regardless of sex, suggesting that gender does not fundamentally alter the drug’s primary toxicity profile (see Supplementary Tables S6, S7). Notably, ROR values for several key bleeding indicators were significantly higher in females than in males, suggesting a potentially heightened sensitivity to anticoagulation or a higher reporting frequency in the female cohort (Table 4). Regarding sex-specific signals, urticaria appeared in the top 50 for females (ROR = 1.22, 95%CI: 0.87–1.72), albeit with a weak signal strength. In contrast, cardiac arrest (ROR = 1.66, 95%CI: 1.07–2.58) and tachycardia (ROR = 1.98, 95%CI: 1.26–3.11) were identified among the top 50 for males but did not reach the same threshold in females.

**Table 4:** Comparison of disproportionate differences in key bleeding indicators between genders in the FAERS database (among the top 50).

PT	Males ROR (95%CI)	Females ROR (95%CI)	Comparison
Muscle haemorrhage	80.82 (62.05–105.27)	397.75 (326.66–484.3)	Higher in the female cohort
Haematoma	52.45 (44.73–61.51)	88.69 (79.12–99.41)	
Intra-abdominal haematoma	Not in the top 50	211 (149.85–297.09)	
Post procedural haematoma	Not in the top 50	152.13 (107.82–214.66)	

PT, preferred term; ROR, reporting odds ratio.

Age-stratified analysis was conducted using the ROR method across three cohorts: pediatric patients (<18 years), adults patients (18–64 years), and the elderly patients (≥65 years). In the pediatric patients, signals were highly fragmented and non-specific; the majority of the top 50 AEs involved only 1–2 cases, leading to extreme statistical fluctuations (e.g., spontaneous haematoma with an ROR of 3,000.39, 95%CI: 370.9–24271.39). In contrast, signal profiles for adults and the elderly were more consolidated and robust, with significantly higher report counts and narrower confidence intervals centered on hematological abnormalities and haemorrhagic events (Supplementary Tables S8–S10). While bleeding remains the core pharmacological risk of fondaparinux sodium across all ages, the signal intensity varied significantly by group (Table 5). We identified distinct age-specific risks: the pediatric patients exhibited unique hepatotoxicity signals including hepatitis (ROR = 71.07, 95%CI: 22.39–225.58), hepatotoxicity (ROR = 44.34, 95%CI: 10.88–180.67), and transaminases increased (ROR = 16.08, 95%CI: 2.24–115.62),which were absent from the top 50 signals in other groups. Conversely, hemiplegia (ROR = 16.37, 95%CI: 11.19–23.93) and skin necrosis (ROR = 34.6, 95%CI: 23.78–50.36) emerged as clinically noteworthy signals within the elderly population.

**Table 5:** Comparison of disproportionate differences in key bleeding indicators between age group in the FAERS database.

PT	Pediatric (N)	Pediatric ROR (95%CI)	Adults (N)	Adults ROR (95%CI)	Elderly (N)	Elderly ROR (95%CI)
Anaemia	/	/	77	5.89 (4.71–7.38)	316	8.01 (7.16–8.97)
Haematoma	/	/	86	58.14 (46.9–72.06)	307	46.74 (41.63–52.47)
Muscle haemorrhage	/	/	43	181.92 (133.91–247.16)	100	114.08 (92.99–139.95)
Melaena	/	/	27	23.12 (15.82–33.78)	91	12.36 (10.04–15.21)
Post procedural haematoma	/	/	12	95.48 (53.85–169.3)	32	83.35 (58.35–119.06)
Heparin-induced thrombocytopenia	/	/	31	73.89 (51.75–105.5)	35	32.88 (23.5–45.99)

PT, preferred term; ROR, reporting odds ratio.

Subgroup analysis by reporter role revealed distinct patterns in signal coverage and clinical focus. Within the healthcare professional (HP) cohort, the vast majority of the top 50 frequent adverse events were identified as positive signals. While the consumer group also demonstrated a high proportion of positive signals, a clear divergence was observed in disproportionality metrics (ROR/IC values) and the specific nature of reported events (Supplementary Tables S11, S12).

The HP group demonstrated superior precision in identifying clinically complex terms and postoperative complications, such as haemoglobin decreased (ROR = 8.47, 95%CI: 7.25–9.89, *N* = 163), hepatic function abnormal (ROR = 4.25, 95%CI: 3.09–5.84, *N* = 38), and post procedural haematoma (ROR = 150.83, 95%CI: 113.08–201.17, *N* = 49). In contrast, consumer reports focused more heavily on visible or symptomatic events, capturing high-intensity signals for localized bleeding, including muscle haemorrhage (ROR = 338.23, 95%CI: 272.46–419.88, *N* = 92), abdominal wall haematoma (ROR = 263.86, 95%CI: 180.5–385.73, *N* = 29), and retroperitoneal haematoma (ROR = 278.02, 95%CI: 187.43–412.4, *N* = 27). Consumers also reported subjective experiences such as dizziness, pain, and falls more frequently, though some failed to meet the threshold for signal strength. Notably, regarding high-risk signals like HIT, both groups showed robust signals, with HPs reporting 61 cases (ROR = 46.62, 95%CI: 36.17–60.1) and consumers exhibiting a remarkably high signal intensity (ROR = 717.5, 95%CI: 512.68–1004.13, *N* = 42).

### 3.5 Sensitivity analysis

To evaluate the robustness of the safety signals and minimize potential confounding from common co-therapies, a sensitivity analysis was conducted by excluding reports involving prednisone, aspirin, and hydroxychloroquine. Following these exclusions, the dataset comprised 783 reports and 1,846 AEs. A comparison of the signal strengths before and after exclusion revealed that the core safety profile of fondaparinux sodium remained highly consistent. Interestingly, among the top 50 persistent potential adverse reactions the signal for post procedural haemorrhage showed an increase in intensity, with the ROR rising from 34.39 (95%CI: 28.6–41.36) to 36.55 (95%CI: 30.34–44.02) after the exclusion of confounders. High-intensity signals such as muscle haemorrhage (ROR = 173.77, 95%CI: 148.9–202.79) and retroperitoneal haematoma (ROR = 108.98, 95%CI: 84.59–140.41) persisted as top-ranking AEs in the sensitivity analysis (Supplementary Table S13). Other critical signals, including haematemesis (ROR = 14.80, 95%CI: 12.16–18.03), renal failure (ROR = 2.12, 95%CI: 1.7–2.65), skin necrosis (ROR = 28.78, 95%CI: 21.08–39.3), anuria (ROR = 7.21, 95%CI: 4.48–11.64), conjunctival discolouration (ROR = 94.52, 95%CI: 30–297.76), aphasia (ROR = 3.96, 95%CI: 2.8–5.6), and coma (ROR = 5.08, 95%CI: 3.97–6.49), remained significant with IC_025_ values consistently above zero, confirming their independent association with fondaparinux sodium use.

### 3.6 The assessment of time to onset for adverse events and log-logistic distribution analysis

**Figure 7:**
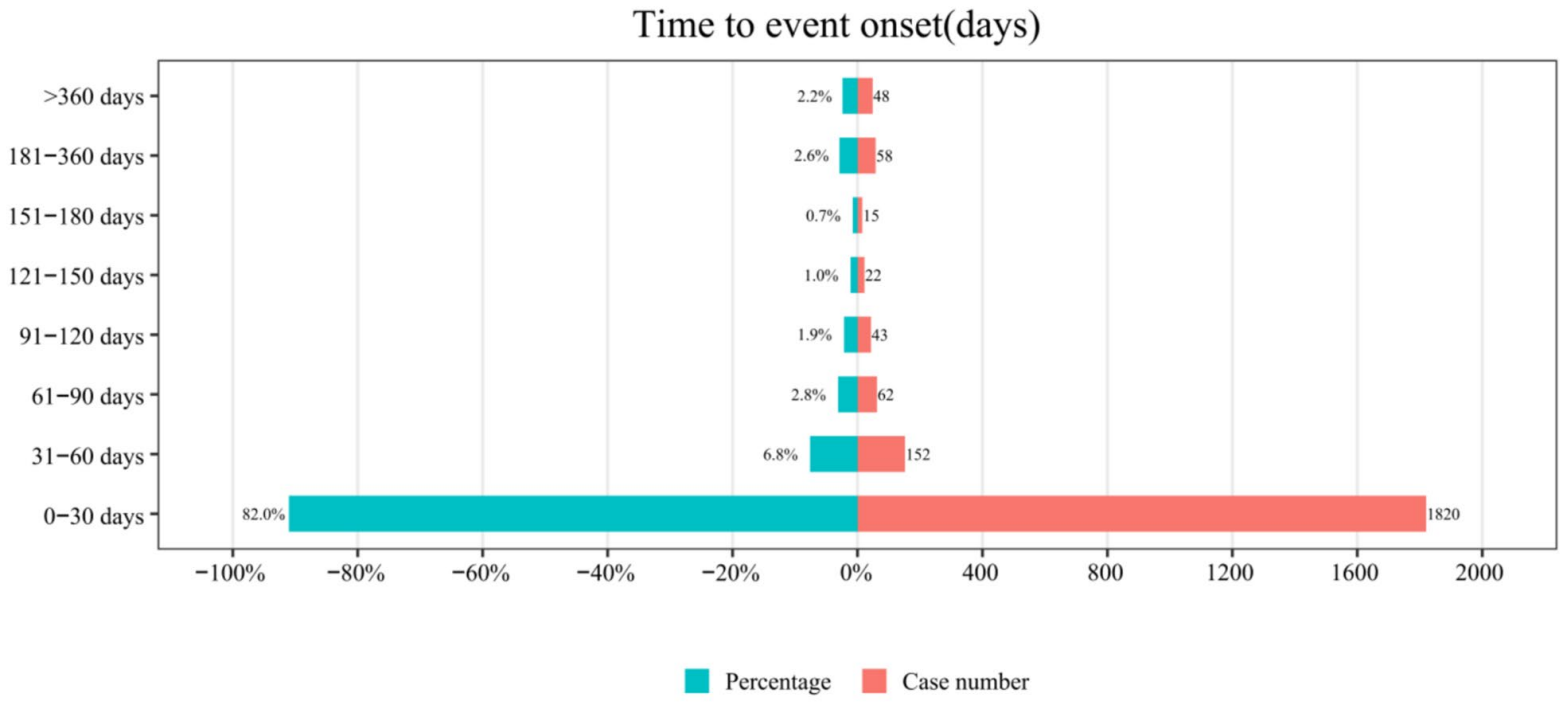
Distribution of time-to-onset for fondaparinux sodium-related adverse events in the FAERS database. The *x*-axis shows the number of cases number and the percentage, and the *y*-axis represents different time intervals (days).

**Figure 8:**
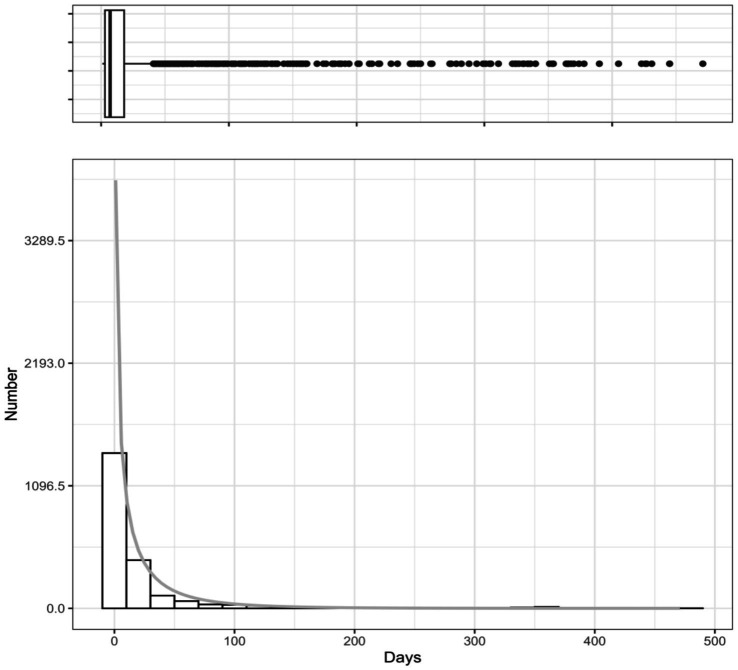
Distribution of time-to-onset for fondaparinux sodium-related adverse events in the FAERS database. The upper panel shows a box plot illustrating the distribution and outliers of onset time, while the lower panel presents a histogram displaying the number of cases over time. The *x*-axis shows time (days), and the *y*-axis represents number of reports.

From the initial 5,700 reports, 3,480 were excluded due to missing START_DT or EVENT_DT information, leaving a refined subset of 2,220 fondaparinux sodium-related AEs with complete temporal data for time-to-onset (TTO) analysis. As illustrated in Figure 7, AE occurrence demonstrated a distinct “early clustering” pattern: 81.98% (N = 1,820) of events occurred within 30 days of administration, followed by a sharp decline in frequency, with only 2.2% occurring after 360 days. Figure 8 presents the density plot and boxplot of the TTO distribution (with the full-range plot available in Supplementary Figure S1). The overall median TTO was 7 days (IQR: 3–19 days), indicating that the risk exposure window is primarily concentrated during the initial phase of treatment.

**Figure 9:**
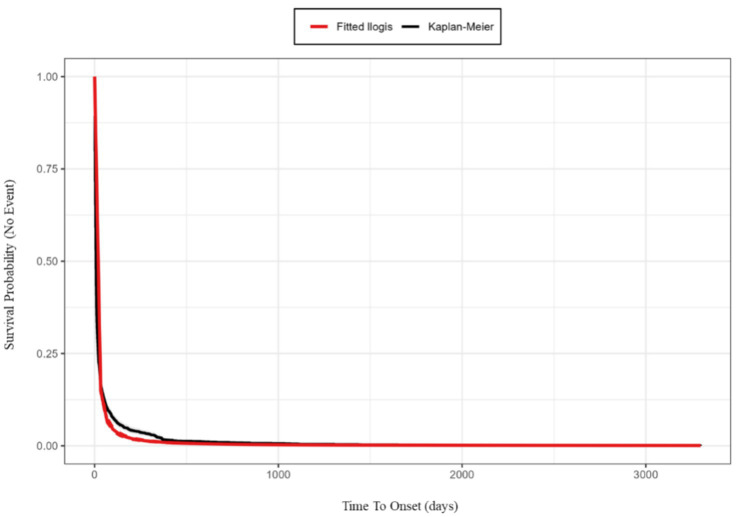
Visual goodness-of-fit plot: Overlay of the fitted log-logistic model and the Kaplan–Meier observed survival curve for time-to-onset data. The black line represents the empirical distribution function (Kaplan–Meier estimate) of the observed data, while the red line represents the theoretical distribution fitted using the log-logistic model.

As emphasized by Maignen et al. (34), the selection of an appropriate parametric model is critical in pharmacovigilance, as imposing an incorrect distribution (e.g., defaulting to Weibull) can obscure the true hazard profile of the adverse event. To ensure statistical rigor, we performed goodness-of-fit tests to compare multiple distribution models. Based on the Akaike Information Criterion (AIC), the optimal model was selected, with detailed comparison results provided in Supplementary Figure S2 and Supplementary Table S14. The Log-logistic distribution demonstrated the best fit (lowest AIC), outperforming the Weibull distribution, which ranked third. By adopting the Log-logistic model, we were able to capture the non-monotonic ‘hump-shaped’ risk pattern that the standard Weibull model would have missed. The visual goodness-of-fit plot of the Log-logistic model is displayed in Figure 9. The Log-logistic analysis yielded a shape parameter (*β*) of 1.2 (95% CI: 1.16–1.24) and a scale parameter (*α*) of 7.8 (95% CI: 7.33–8.27). Notably, a shape parameter β > 1 in the Log-logistic model indicates a unimodal hazard function, characterized by a rapid increase in risk to an early peak (consistent with the observed median of 7 days) followed by a gradual decline. This “early-peak” profile offers a more precise characterization of the hazard dynamics compared to the monotonic “early failure” model. The comprehensive statistical properties results are summarized in Table 6.

**Table 6:** Time to onset of fondaparinux sodium-associated adverse events and log-logistic model in the FAERS database.

Drug	TTO(days)		Log-logistic model		
	Case reports	Median days (IQR)	Scale parameter (95%CI)	Shape parameter (95%CI)	Pattern
Fondaparinux sodium	2,220	7(3–19)	7.8 (7.33–8.27)	1.2 (1.16–1.24)	early peak

TTO, time to onset; CI, confidence interval; IQR, interquartile range.

After establishing the overall ‘early peak’ pattern of fondaparinux sodium-related AEs, we performed stratified analyses on two representative high-risk clusters:bleeding (pharmacologically driven) and rash (immunologically driven). This sub-group analysis aimed to verify the robustness of the general temporal trend across different physiological systems and to identify specific clinical monitoring windows for distinct types of adverse reactions.

Stratified analysis revealed distinct temporal dynamics between bleeding and rash risks, despite their shared ‘early-clustering’ profile (Table 7). Subgroup analysis revealed that rash events (*N* = 54) manifested significantly earlier, with a median onset of 5.5 days (IQR: 2–12.5 days), compared to 7 days for haemorrhagic events (*N* = 778, IQR: 3–13.5 days). Regarding the temporal distribution, rash-related reactions exhibited a more pronounced early-peak pattern. This “early-peak” pattern is characteristic of drug-induced hypersensitivity reactions (DHRs), which are typically triggered by immune-mediated mechanisms shortly after drug exposure. Although the onset of bleeding occurred later than rash, both clusters showed a high cumulative incidence (>87%) within the first 30 days.

**Table 7:** Stratified analysis of the timing of different categories of adverse reactions in the FAERS database.

Index	Total AEs (*N* = 2,220)	Bleeding (*N* = 778)	Rash (*N* = 54)
Median	7 days	7 days	5.5 days
IQR	3–19 days	3.0–13.5 days	2.0–12.5 days
Very early stage proportion (≤7 days)	51.98%	55.01%	59.26%
First month percentage (≤30 days)	81.98%	87.28%	92.59%

## 4 Discussion

The four pharmacovigilance algorithms employed (ROR, PRR, EBGM, and BCPNN) exhibited varying sensitivities and degrees of overlap in identifying fondaparinux sodium-related adverse events PTs. Notably, 260 PTs (32.3% of the total) were consistently identified by all four methods in the FAERS database. This intersection set is regarded as a collection of high-confidence signals. From a statistical perspective, the convergence of frequentist and Bayesian models minimizes random errors inherent to any single approach, thereby enhancing the overall robustness of the findings. In this study, well-established adverse reactions—such as haemorrhage, haematoma, HIT, and anaemia—were all captured within this 260-signal subset, validating the reliability of this multi-algorithmic approach in identifying confirmed risks.

However, significant variation in signal volume was observed across methods, with BCPNN identifying 806 PTs—a count substantially higher than the other three algorithms. This discrepancy reflects the inherent trade-off between detection efficacy and the false-positive rate. The synergistic application of these four algorithms provides a complementary perspective for the safety evaluation of fondaparinux sodium: while the shared signals ensure the stability of our core conclusions, high-sensitivity methods like BCPNN expand the search scope to mitigate the risk of missing rare but serious safety signals.

This study is the first to conduct a broad and systematic pharmacovigilance survey of fondaparinux sodium-related adverse events using the FAERS, JADER, and VigiAccess databases. This study offers a comprehensive, real-world assessment of the safety profile of fondaparinux sodium. Through analysis of data from the FAERS database, the adverse reactions listed in the fondaparinux sodium drug instructions, including bleeding, hematoma, anemia and HIT, were confirmed.

Bleeding has been described as a common adverse effect of fondaparinux sodium in several studies. Cheng et al. (35) reported in a phase III clinical study that fondaparinux sodium demonstrated significant efficacy in preventing thromboembolism after hip or knee replacement surgery but was correlated with a heightened risk of bleeding. Similarly, Kumar et al. (36) reported that, compared with low-molecular-weight heparins, fondaparinux sodium was associated with a greater risk of bleeding. Our study results are consistent with previous findings, emphasizing the importance of individual patient assessment and close monitoring and management of this adverse reaction when fondaparinux sodium is used clinically. Another notable finding is the occurrence of hematologic and lymphatic system abnormalities, such as thrombocytopenia and anemia, which are consistent with our study results. Several cases in which fondaparinux sodium can induce HIT have been reported (37–39). Chen et al. (40) reported that fondaparinux sodium at a specific concentration forms large, stable complexes with PF4, promoting the binding of the HIT-like monoclonal KKO antibody and increasing platelet aggregation and activation. Schrag et al. (41) highlighted in their study that anaemia is one of the most common adverse reactions associated with heparin, including fondaparinux sodium. Therefore, when fondaparinux sodium is used clinically, vigilance for the possibility of hematologic and lymphatic system abnormalities is warranted.

Through disproportionality analysis of the FAERS database, we identified several safety signals not explicitly documented in current labeling or previously considered clinically rare, such as haematemesis, renal failure, skin necrosis, anuria, conjunctival discolouration, aphasia, and coma. External validation provided further context for these findings. In the JADER database, positive signals were independently verified for unlabelled events, specifically haematemesis and skin necrosis. Furthermore, while formal signal detection could not be performed on VigiAccess data due to the lack of detailed background information, the occurrence of these events was documented in the global dataset, including records for haematemesis, renal failure, skin necrosis, coma, aphasia, and anuria. While the presence of these reports across multiple databases lends support to our observations, it is imperative to clarify that statistical associations derived from spontaneous reporting systems (SRS) do not equate to definitive causal relationships.

Our findings significantly extend the recent work by Lu et al. (42), who investigated the safety profiles of subcutaneous anticoagulants using FAERS data through Q2 2024. While both studies observe similar demographic trends, such as female predominance and age distribution, our research offers a more extensive and granular evaluation. The discrepancy in reporting volume and signal detection primarily stems from our broader inclusion criteria and extended observation period (up to Q3 2024). Unlike the cited study, which restricted its analysis to HP reports and VTE-related indications, our study adopted a more inclusive approach.

This comprehensive strategy markedly enhanced our detection sensitivity and statistical power. For instance, we identified 67 cases of coma, 106 cases of haematemesis and 41 cases of skin necrosis, a 3-to 8-fold increase compared to the 12, 12 and 11 cases reported by Lu et al. (42), respectively. This larger sample size yields more robust statistical estimates with narrower confidence intervals. Furthermore, by employing four-algorithm cross-validation, we uncovered critical unlabelled signals not documented in their report, most notably renal failure (*N* = 91), anuria (*N* = 20), aphasia (*N* = 35) and conjunctival discolouration (*N* = 3). These findings fill a crucial gap in the existing literature and offer a more comprehensive safety reference for clinical practice.

The associations observed for coma and aphasia must be interpreted with caution. Given that fondaparinux sodium is primarily indicated for ACS and Deep Vein Thrombosis (DVT), these patients are inherently at high risk for thromboembolic or haemorrhagic complications. Clinically, these neurological events likely reflect sequelae of major cerebral infarction or secondary intracranial haemorrhage following potent anticoagulation, rather than direct neurotoxicity. This “confounding by indication” or “reverse causality” represents a primary limitation of disproportionativity analysis.

The high ROR values observed for rare adverse events must be interpreted with caution. In pharmacovigilance studies, disproportionality estimates often exhibit increased variability and potential inflation when based on a very small number of reports (25). To ensure the reliability of our findings, we applied a minimum threshold of *N* ≥ 3, consistent with the methodology (26). Regarding the extreme ROR for conjunctival discolouration (*N* = 3, ROR = 87.42), the biological plausibility remains low. As a selective Factor Xa inhibitor, fondaparinux sodium lacks the physicochemical properties to alter tissue pigmentation. Given the robust signals for haemorrhagic events (e.g., haematoma, *N* = 562, ROR = 77.88), we hypothesize this is a result of coding bias, where subconjunctival haemorrhage was misclassified during data entry. Such rare terms with minimal denominators often exhibit violent statistical fluctuations and should be viewed as potential false-positive signals due to sparse data bias rather than genuine safety risks. These results remain hypothesis-generating and require corroboration through clinical observation or broader epidemiological research.

Conversely, some potential novel signals possess clear biological rationales. The significant association with haematemesis (ROR = 14.52, 95%CI: 11.99–17.58) aligns perfectly with the pharmacological inhibition of the coagulation cascade, which elevates the risk of gastrointestinal mucosal bleeding (43). Similarly, the skin necrosis signal (ROR = 34.6, 95%CI: 23.78–50.36) identified in the elderly patients, although rare, is supported by literature suggesting that anticoagulant therapy may trigger microvascular thrombosis or idiosyncratic HIT-like immune responses, eventually culminating in tissue necrosis. This indicates that skin necrosis may serve as a clinically significant, albeit rare, warning signal for this population.

Skin necrosis can affect individuals of any age, leading to physiological issues such as pain and a heightened risk of infection (44, 45). It not only affects daily life but can also lead to sleep disturbances, decreased appetite and restricted mobility. Skin necrosis can also have profound psychological impacts; changes in appearance due to skin necrosis may lead to feelings of inferiority and reduced self-confidence. The relevant literature has reported skin reactions associated with anticoagulants, including coumarin derivatives and low-molecular-weight heparins. The skin manifestations caused by anticoagulants can range from localized allergic reactions to skin necrosis (46–48). At the start of warfarin therapy, a decrease in protein C can disrupt the coagulation–anticoagulation balance, resulting in a hypercoagulable state that may cause thrombosis and warfarin-related skin necrosis. Heparin-induced skin necrosis (HI5N) was first documented in 1973 by O’Toole (49), who observed four patients with local erythema and necrosis at subcutaneous heparin injection sites.

The literature suggests that the mechanism underlying heparin-induced skin necrosis involves an immune-mediated process in which heparin–antibody complexes bind to platelets, causing platelet aggregation, thromboembolism, and ischemic necrosis (50, 51). This mechanism may also be associated with HIT, as HIT can also occur during immune-mediated platelet aggregation. Additionally, heparin-induced skin necrosis may be related to isolated protein C deficiency or combined deficiencies of protein C and protein S. Fondaparinux sodium, a type of heparin, can also potentially cause thrombocytopenia. Therefore, awareness of the risk of skin necrosis in patients receiving fondaparinux sodium therapy is crucial. Understanding these mechanisms and risk factors can help in the early identification and management of such adverse reactions.

In this study, HIT (ROR = 79.66, 95%CI: 65.94–96.23) was identified as a strong positive signal. Although clinical studies report an extremely low incidence of fondaparinux sodium-induced HIT (<0.1), its high frequency in the FAERS database largely stems from protopathic bias, as the drug is frequently utilized as an alternative therapy for patients already suspected of having HIT (52, 53). This discrepancy can be attributed to the inherent nature of disproportionality analysis; the ROR reflects the strength of statistical association within the database rather than actual clinical incidence (27). This finding aligns with highlighting the necessity of accounting for clinical context to mitigate confounding during data interpretation.

The precise mechanism through which fondaparinux sodium causes renal impairment has yet to be determined. Notably, our study identified a consistent cluster of renal-related signals in the FAERS database: anuria (ROR = 7.85, 95% CI: 5.06–12.18) and renal failure (ROR = 2.29, 95% CI: 1.87–2.82). The occurrence of these events was further documented in the VigiAccess global database (39 cases of renal failure and 6 cases of anuria), suggesting a widespread reporting pattern.

As appropriately highlighted in critical care literature, patients receiving anticoagulants often suffer from severe underlying conditions, such as septic shock, acute heart failure, or major post-surgical complications (54, 55). These conditions are independent risk factors for hemodynamic instability and multi-organ dysfunction, including acute kidney injury (AKI) and anuria. Therefore, the high ROR values observed in our study may, to some extent, reflect confounding by indication, where the underlying severity of the patient’s condition rather than the drug itself precipitates the renal event.

Despite the potential for confounding, a biological basis for fondaparinux-associated renal exacerbation remains highly plausible, particularly regarding drug accumulation. Fondaparinux sodium demonstrates significant bioactivity following subcutaneous injection, with over 70% of the drug excreted unchanged via the kidneys (56). Its clearance is strictly correlated with renal function. Boneu et al. (57) reported that in elderly volunteers (aged 65–83 years) with reduced creatinine clearance (60 ± 15 mL/min), the plasma clearance of the drug significantly decreased compared to younger volunteers (aged 18–32 years, 132 ± 20 mL.min-1), resulting in a prolonged half-life (17 h vs. 21 h) (6). A correlation was observed between CLCr and the plasma clearance of the drug, indicating that the clearance rate of fondaparinux sodium decreases as renal function declines (58). Since renal function naturally deteriorates with age, elderly patients face a dual risk: reduced elimination capacity and increased drug exposure. In approximately two-thirds of elderly patients, renal excretion decreases by up to 50%, leading to substantial accumulation.

This accumulation can have clinical consequences. Studies indicate that patients with mild to moderate renal dysfunction exhibit median anti-Xa activity levels approximately 18–47% higher than those with normal renal function by postoperative day 7, with the gap widening to 30–80% by day 14 (59). In this context, while fondaparinux sodium may not be the sole initiator of renal failure, drug accumulation in patients with pre-existing renal compromise (CrCl < 30 mL/min) could exacerbate the renal burden, potentially driving the progression from renal insufficiency to anuria. Therefore, while the signals for anuria and renal failure likely represent a composite of underlying disease severity and drug effects, they underscore a critical safety message. Fondaparinux sodium should be administered with heightened caution in elderly patients and is contraindicated in severe renal impairment. Clinicians must rigorously monitor urine output and renal function, not only to prevent bleeding but also to detect early signs of drug accumulation that could worsen renal outcomes.

While the core safety profiles for both sexes remained highly consistent—dominated by haemorrhagic and haematoma-related events—female patients exhibited significantly higher signal intensities across multiple bleeding-related PTs. For instance, the ROR for muscle haemorrhage in females (397.75) markedly exceeded that in males (80.82). This divergence may be attributed to physiological factors, such as lower average body weight and sex-specific variations in renal clearance. Given that fondaparinux sodium is primarily excreted unchanged via the kidneys, standard dosing regimens may result in higher systemic exposure in females. Furthermore, the use of fondaparinux sodium for thromboprophylaxis during pregnancy may contribute to this trend (60). However, as FAERS lacks granular data on patient weight and dosage, these associations may be confounded by physiological differences. Additionally, fluctuations in oestrogen levels potentially modulate vascular endothelial function and haemostatic balance, further complicating the risk profile.

Age-stratified analysis revealed distinct risk shifts, highlighting the unique vulnerabilities of the elderly and the specific safety concerns in the pediatric population. In elderly patients (≥65 years): Both the absolute report counts and the signal intensity for anaemia were significantly higher than in the adult group. This likely reflects a heightened sensitivity to chronic anticoagulant-induced blood loss, potentially due to diminished organ reserves, increased tissue fragility, and polypharmacy. Furthermore, the emergence of specific signals such as hemiplegia and skin necrosis suggests more severe clinical sequelae following adverse events in this demographic.

In pediatric patients (<18 years), the sample size for this group was relatively limited (N < 3 for most PTs), resulting in extreme fluctuations in ROR values. However, in pharmacovigilance, the primary objective for rare populations like children is to identify severe latent hazards masked by low reporting frequencies. Despite the small N, the identified hepatotoxicity signals align biologically with published case reports (61) and FDA pediatric safety reviews. Given the unique developmental stage of the pediatric liver and immune system, these signals may reflect a rare idiosyncratic drug-induced liver injury (DILI). Nevertheless, due to the denominator limitations of the FAERS database, the magnitude of these ROR values should be interpreted with caution. Further validation through large-scale prospective pediatric patients is recommended.

HPs and consumers revealed significant divergence in data precision and clinical focus. HP reports were characterized by signals requiring objective medical validation, such as haemoglobin decreased and hepatic function abnormal, underscoring the indispensable role of professionals in monitoring biochemical toxicity. In contrast, the consumer cohort exhibited higher signal intensities for visible or localized events, such as muscle haemorrhage and abdominal wall haematoma, alongside subjective reports of pain and dizziness. This pattern reflects a heightened consumer sensitivity to symptomatic manifestations and impairments in quality of life. Notably, the remarkably high ROR for HIT in the consumer group (ROR = 717.5) may be susceptible to information bias or misclassification, as patients may tend to categorize various platelet-related concerns under this high-profile clinical term. These findings suggest that while consumer data provide valuable symptomatic insights, medically validated reports from HPs should remain the primary reference for evaluating complex safety signals.

Our analysis of the time-to-onset (TTO) data reveals a distinct ‘early-peak’ risk profile for fondaparinux sodium. The majority of adverse events (81.98%) occurred within the first month of therapy, with the risk concentration peaking rapidly within the first week. This observation is statistically corroborated by our Log-logistic model, where the estimated scale parameter (*α* = 7.8 days) aligns remarkably with the observed median TTO of 7 days. Furthermore, the shape parameter (*β* = 1.2) indicates a unimodal hazard function, suggesting that the risk does not merely decrease monotonically from day one (as implied by an early failure Weibull model), but rather escalates quickly to a peak around the first week before gradually subsiding.

Clinically, this temporal distribution likely reflects the pharmacokinetic properties of fondaparinux sodium, which reaches peak plasma concentrations within 2–3 h and achieves steady-state rapidly. The concentration of events in the first 30 days, and particularly the median onset at Day 7, defines a critical ‘high-vigilance window.’ This suggests that if patients tolerate the initial month of therapy without incident, the probability of late-onset adverse events diminishes significantly. Consequently, clinicians should prioritize intensive monitoring, primarily during the first week of administration to mitigate early-onset risks.

Subgroup analysis further elucidated the varying kinetics of risk release across different mechanisms. The occurrence of rash events (*N* = 54) was nearly 60% within the first week (≤7 days) and reached 92.59% within the first month (≤30 days), demonstrating a distinct early-onset characteristic. Although haemorrhagic events (*N* = 778) also frequently occurred in the early stages, the upper limit of their interquartile range (IQR) (13.5 days) was slightly higher than that of skin rashes. Rash-related signals exhibit a prominent ‘front-loaded’ characteristic, consistent with immune-mediated acute or delayed hypersensitivity; whereas haemorrhagic signals follow the principles of pharmacokinetic accumulation, lagging behind rash in median onset and demonstrating a more extended duration of risk. These temporal patterns carry key clinical implications: the first week of fondaparinux sodium therapy is a critical window for detecting early dermatological hypersensitivity, while vigilance for haemorrhagic complications must be sustained throughout the entire treatment course.

Despite the diverse physiological mechanisms across organ systems, the AE distribution demonstrated remarkable consistency within the Log-logistic model. This unity in heterogeneity provides a clear clinical roadmap: concentrating monitoring resources within the first month of treatment not only aligns with drug risk dynamics but also optimizes healthcare resource allocation. Furthermore, our findings underscore the importance of demographic-specific vigilance. Notably, the potential risks of hepatotoxicity in pediatric patients and skin necrosis in elderly patients necessitate a heightened clinical index of suspicion, as identified in our subgroup analysis. Implementing individualized surveillance, such as routine hepatic enzyme monitoring for pediatric patients and rigorous integumentary evaluations for the elderly patients, is essential for mitigating these age-specific risks during fondaparinux sodium therapy.

## 5 Limitations

This study has several limitations that warrant consideration. First, the inherent limitations of SRS must be acknowledged, as these systems are characterized by reporting bias, under-reporting, and a lack of clinical granularity. Specifically, the absence of detailed information including medical history, exact dosage, concomitant medications, and precise temporal links constrains the depth of AE assessment. Second, regarding methodological constraints, the lack of denominator data in the FAERS database representing the total exposed population precludes the calculation of true incidence rates or absolute risks. Consequently, disproportionality analysis serves as a hypothesis-generating tool rather than a confirmatory one and cannot establish definitive causality. Third, clinical confounding must be acknowledged. This study may be influenced by confounding by indication, where fondaparinux sodium is predominantly prescribed to high-risk populations (e.g., elderly patients or those post-major surgery with multiple comorbidities), potentially inflating the statistical association of events like anaemia and haemorrhage.

Finally, our study is strengthened by cross-validation with VigiAccess and the JADER database. It is important to acknowledge that heterogeneity exists between these data sources. While the qualitative presence of some potential signals was consistently observed across all three platforms, the quantitative proportional distribution of adverse events in VigiAccess and JADER did not perfectly mirror the FAERS findings. This discrepancy likely reflects inherent differences in global reporting cultures, regulatory frameworks, and population demographics. However, the identification of these critical signals across diverse independent databases, while exhibiting variations in relative frequencies, reinforces the conclusion that these findings are not artifacts of a single reporting system. Instead, they reflect genuine and variable safety concerns within the real-world population.

Furthermore, the inability to fully stratify results by drug role codes, such as primary suspect versus concomitant medications, may introduce confounding bias. Although this study prioritized reports listing fondaparinux sodium as a suspect drug, the inherent ambiguity in role assignment within the FAERS database remains a limitation. Nevertheless, the strong consistency between identified core signals and established pharmacological mechanisms, particularly for haemorrhage, suggests these findings are robust for hypothesis generation and clinical safety monitoring. Future large-scale prospective studies or multicenter collaborative research utilizing refined datasets are essential to isolate the independent effects of fondaparinux sodium and provide more definitive evidence for clinical decision-making.

## 6 Conclusion

This comprehensive pharmacovigilance study utilizes the FAERS database to delineate the real-world safety profile of fondaparinux sodium (Q1 2004 to Q3 2024), with key signals cross-validated using the VigiAccess database (Q2 2003 to Q4 2025) and JADER database (covering Q2 2007 to Q2 2025). Based on goodness-of-fit tests, the Log-logistic distribution (*β* > 1) best characterized the time-to-onset data, revealing a distinct early-peak hazard pattern where risks are predominantly concentrated within the first week of treatment. Crucially, through this multi-database cross-validation, several unbalanced signals were identified and corroborated, most notably haematemesis and skin necrosis. These findings provide hypothesis-generating evidence for potential unlabelled risks, warranting increased clinical vigilance and further epidemiological investigation.

While the core toxicity profile remains consistent with established labeling, demographic stratifications highlighted specific vulnerabilities: heightened bleeding intensity in females, potential hepatotoxicity in pediatric patients, and skin necrosis in the elderly. Sensitivity analyses further confirmed the robustness of these associations. Regarding potential novel signals such as anuria, and renal failure, we recommend cautious interpretation. While statistically significant, these events may partly reflect confounding by indication in critically ill patients with multi-organ dysfunction. Consequently, these findings should be viewed as hypothesis-generating evidence, underscoring the need for vigilant clinical monitoring and personalized risk stratification in complex clinical settings.

## Data Availability

The completed READUS-PV checklist associated with this research can be found in Supplementary Table S15. The data presented in this study are available on request from the corresponding author.

## Footnotes

1
https://www.vigiaccess.org
