# Supplementary material for: Adverse event profile of fondaparinux sodium: a disproportionality analysis based on FAERS, JADER, and VigiAccess databases
Source: Front Med (Lausanne). 2026 Feb 5;13:1731378. doi: 10.3389/fmed.2026.1731378 (PMC12916646; doi:10.3389/fmed.2026.1731378)
Supplement: Supplementary file 1 [file Supplementary_file_1.docx]

**Supplementary Material**

**Supplementary Tables**

Supplementary Table 1:

Two-by-two contingency table for disproportionality analyses.

|  | Target AEs | Other AEs | Total |
| --- | --- | --- | --- |
| Target drug | a | b | a+b |
| Other drugs | c | d | c+d |
| Total | a+c | b+d | a+b+c+d |

Abbreviation: AEs, adverse events; a, number of reports containing both the target drug and target adverse drug reaction; b, number of reports containing other adverse drug reaction of the target drug; c, number of reports containing the target adverse drug reaction of other drugs; d, number of reports containing other drugs and other adverse drug reactions.

Supplementary Table 2:

Four major algorithms used for signal detection.

| Algorithms | Equation | Criteria |
| --- | --- | --- |
| ROR | ROR=ad/b/c | lower limit of 95% CI>1, N≥3 |
|  | 95%CI=e^ln(ROR)±1.96(1/a+1/b+1/c+1/d)^0.5^ |  |
| PRR | PRR=a(c+d)/c/(a+b) | PRR≥2, χ^2^≥4, N≥3 |
|  | χ^2^=[(ad-bc)^2](a+b+c+d)/[(a+b)(c+d)(a+c)(b+d)] |  |
| BCPNN | IC=log_2_a(a+b+c+d)(a+c)(a+b) | IC_025_>0 |
|  | 95%CI= E(IC) ± 2V(IC)^0.5 |  |
| MGPS | EBGM=a(a+b+c+d)/(a+c)/(a+b) | EBGM_05_>2 |
|  | 95%CI=e^ln(EBGM)±1.96(1/a+1/b+1/c+1/d)^0.5^ |  |

Abbreviation: a, number of reports containing both the target drug and target adverse drug reaction; b, number of reports containing other adverse drug reaction of the target drug; c, number of reports containing the target adverse drug reaction of other drugs; d, number of reports containing other drugs and other adverse drug reactions. 95%CI, 95% confidence interval; N, the number of reports; χ2, chi-squared; IC, information component; IC025, the lower limit of 95% CI of the IC; E(IC), the IC expectations; V(IC), the variance of IC; EBGM, empirical Bayesian geometric mean; EBGM05, the lower limit of 95% CI of EBGM.

Supplementary Table 3:

Signal strength of fondaparinux sodium AEs across SOC in the JADER database.

| System Organ Class (SOC) | Case  numbers | ROR  (95%CI) | PRR  (95%CI) | χ^2^ | EBGM  (EBGM_05_) | IC  (IC_025_) |
| --- | --- | --- | --- | --- | --- | --- |
| Injury, poisoning and procedural complications* | 290 | 10.73(9.39-12.26) | 8.27(7.26-9.41) | 1899.26 | 8.22(7.19) | 3.04(1.37) |
| Gastrointestinal disorders* | 197 | 2.39(2.05-2.79) | 2.15(1.85-2.5) | 131.89 | 2.15(1.84) | 1.1(-0.56) |
| Vascular disorders* | 139 | 5.18(4.34-6.19) | 4.68(3.92-5.58) | 410.86 | 4.66(3.9) | 2.22(0.55) |
| Investigations* | 137 | 1.35(1.13-1.61) | 1.31(1.09-1.56) | 10.75 | 1.3(1.09) | 0.38(-1.28) |
| Blood and lymphatic system disorders | 72 | 1.01(0.79-1.28) | 1.01(0.79-1.28) | 0 | 1.01(0.79) | 0.01(-1.66) |
| Skin and subcutaneous tissue disorders | 57 | 0.83(0.63-1.08) | 0.84(0.64-1.09) | 1.95 | 0.84(0.64) | -0.26(-1.93) |
| Nervous system disorders | 49 | 0.43(0.32-0.57) | 0.46(0.34-0.61) | 35.24 | 0.46(0.34) | -1.13(-2.8) |
| General disorders and administration site conditions | 45 | 0.57(0.42-0.76) | 0.58(0.43-0.79) | 14.3 | 0.58(0.43) | -0.78(-2.44) |
| Hepatobiliary disorders | 43 | 1.01(0.75-1.37) | 1.01(0.75-1.37) | 0 | 1.01(0.75) | 0.02(-1.65) |
| Musculoskeletal and connective tissue disorders | 33 | 1.07(0.76-1.51) | 1.07(0.76-1.51) | 0.15 | 1.07(0.76) | 0.09(-1.57) |
| Infections and infestations | 18 | 0.18(0.11-0.28) | 0.19(0.12-0.3) | 68.21 | 0.19(0.12) | -2.4(-4.07) |
| Renal and urinary disorders | 16 | 0.35(0.21-0.57) | 0.35(0.22-0.58) | 19.6 | 0.35(0.22) | -1.5(-3.16) |
| Respiratory, thoracic and mediastinal disorders | 12 | 0.14(0.08-0.24) | 0.15(0.08-0.26) | 64.96 | 0.15(0.08) | -2.78(-4.45) |
| Metabolism and nutrition disorders | 9 | 0.17(0.09-0.33) | 0.18(0.09-0.34) | 35.7 | 0.18(0.09) | -2.49(-4.16) |
| Reproductive system and breast disorders* | 9 | 2.04(1.06-3.94) | 2.03(1.06-3.92) | 4.74 | 2.03(1.05) | 1.02(-0.65) |
| Cardiac disorders | 4 | 0.08(0.03-0.22) | 0.09(0.03-0.23) | 41.05 | 0.09(0.03) | -3.55(-5.22) |
| Congenital, familial and genetic disorders | 4 | 1.36(0.51-3.63) | 1.36(0.51-3.62) | 0.38 | 1.36(0.51) | 0.44(-1.23) |
| Neoplasms benign, malignant and unspecified (incl cysts and polyps) | 3 | 0.06(0.02-0.18) | 0.06(0.02-0.19) | 44.6 | 0.06(0.02) | -4.01(-5.68) |
| Surgical and medical procedures | 3 | 0.56(0.18-1.73) | 0.56(0.18-1.74) | 1.05 | 0.56(0.18) | -0.84(-2.51) |
| Eye disorders | 2 | 0.11(0.03-0.46) | 0.12(0.03-0.46) | 13.73 | 0.12(0.03) | -3.11(-4.78) |
| Psychiatric disorders | 2 | 0.09(0.02-0.35) | 0.09(0.02-0.35) | 19.29 | 0.09(0.02) | -3.51(-5.17) |
| Ear and labyrinth disorders | 1 | 0.4(0.06-2.85) | 0.4(0.06-2.86) | 0.89 | 0.4(0.06) | -1.31(-2.98) |
| Pregnancy, puerperium and perinatal conditions | 1 | 0.21(0.03-1.52) | 0.21(0.03-1.52) | 2.89 | 0.21(0.03) | -2.22(-3.89) |

Abbreviation: Asterisks (*) indicate positive signals in algorithm; ROR, reporting odds ratio; PRR, proportional reporting ratio; EBGM, empirical Bayesian geometric mean; EBGM05, the lower limit of the 95% CI of EBGM; χ2, chi-squared; IC, information component; IC025, the lower limit of the 95% CI of the IC; CI, confidence interval; AEs, adverse events.

Supplementary Table 4:

List of 260 PTs with positive signals across four disproportionality algorithms in the FAERS database.

| PT | Case  numbers | ROR  (95%CI) | PRR  (95%CI) | χ^2^ | EBGM  (EBGM_05_) | IC  (IC_025_) |
| --- | --- | --- | --- | --- | --- | --- |
| Haematoma | 562 | 77.88 (71.53-84.79) | 75.34, (75.26-75.43) | 40272.33 | 73.59 (68.54) | 6.2 (6.08) |
| Anaemia | 498 | 9.29 (8.5-10.16) | 9.05, (8.96-9.13) | 3566.04 | 9.02 (8.37) | 3.17 (3.04) |
| Haemorrhage | 331 | 11.53 (10.34-12.86) | 11.32, (11.22-11.43) | 3109.51 | 11.29 (10.3) | 3.5 (3.34) |
| Haemoglobin decreased | 279 | 9.48 (8.42-10.67) | 9.34, (9.22-9.46) | 2075.03 | 9.31 (8.44) | 3.22 (3.05) |
| Pulmonary embolism | 277 | 10.1 (8.97-11.37) | 9.95, (9.83-10.07) | 2226.9 | 9.92 (8.98) | 3.31 (3.14) |
| Muscle haemorrhage | 194 | 183.69 (158.81-212.47) | 181.62, (181.47-181.76) | 32930.34 | 171.67 (151.99) | 7.42 (7.21) |
| Deep vein thrombosis | 188 | 9.83 (8.51-11.35) | 9.73, (9.59-9.88) | 1470.15 | 9.71 (8.6) | 3.28 (3.07) |
| Cerebral haemorrhage | 175 | 17.24 (14.85-20.01) | 17.07, (16.92-17.22) | 2634.63 | 16.98 (14.99) | 4.09 (3.87) |
| Thrombosis | 172 | 7.47 (6.43-8.68) | 7.4, (7.25-7.55) | 951.55 | 7.39 (6.51) | 2.89 (2.66) |
| Shock haemorrhagic | 162 | 74.42 (63.64-87.03) | 73.73, (73.57-73.88) | 11354.79 | 72.05 (63.2) | 6.17 (5.94) |
| Melaena | 149 | 23.56 (20.04-27.7) | 23.36, (23.2-23.53) | 3167.22 | 23.2 (20.26) | 4.54 (4.3) |
| Thrombocytopenia | 142 | 4.54 (3.85-5.35) | 4.51, (4.34-4.67) | 387.87 | 4.5 (3.92) | 2.17 (1.93) |
| Gastrointestinal haemorrhage | 120 | 4.83 (4.04-5.79) | 4.81, (4.63-4.99) | 361.77 | 4.8 (4.13) | 2.26 (2) |
| Oedema peripheral | 120 | 3.35 (2.8-4.01) | 3.33, (3.16-3.51) | 196.29 | 3.33 (2.87) | 1.74 (1.47) |
| Drug interaction | 115 | 2.53 (2.11-3.04) | 2.52, (2.34-2.7) | 105.71 | 2.52 (2.16) | 1.33 (1.06) |
| Post procedural haemorrhage | 115 | 34.39 (28.6-41.36) | 34.17, (33.99-34.35) | 3663.41 | 33.81 (28.98) | 5.08 (4.81) |
| Product quality issue | 111 | 2.77 (2.3-3.33) | 2.76, (2.57-2.94) | 124.28 | 2.75 (2.36) | 1.46 (1.19) |
| Heparin-induced thrombocytopenia | 111 | 79.66 (65.94-96.23) | 79.15, (78.96-79.34) | 8353.44 | 77.21 (65.92) | 6.27 (5.99) |
| Haematemesis | 106 | 14.52 (11.99-17.58) | 14.43, (14.24-14.62) | 1319.52 | 14.37 (12.24) | 3.84 (3.56) |
| Subdural haematoma | 95 | 22.95 (18.74-28.09) | 22.82, (22.62-23.02) | 1968.45 | 22.66 (19.13) | 4.5 (4.21) |
| Haematuria | 85 | 8.51 (6.87-10.53) | 8.47, (8.26-8.69) | 559.04 | 8.45 (7.07) | 3.08 (2.77) |
| Contusion | 82 | 3.02 (2.43-3.75) | 3.01, (2.79-3.22) | 109.87 | 3 (2.51) | 1.59 (1.27) |
| Platelet count decreased | 81 | 2.66 (2.14-3.31) | 2.66, (2.44-2.87) | 83.72 | 2.65 (2.21) | 1.41 (1.09) |
| Epistaxis | 75 | 3.5 (2.79-4.39) | 3.49, (3.26-3.72) | 133.31 | 3.49 (2.88) | 1.8 (1.47) |
| Pallor | 71 | 9.02 (7.14-11.39) | 8.99, (8.76-9.22) | 502.88 | 8.97 (7.37) | 3.16 (2.82) |
| Injection site haematoma | 70 | 15.65 (12.37-19.8) | 15.59, (15.35-15.82) | 951.16 | 15.52 (12.74) | 3.96 (3.61) |
| Retroperitoneal haematoma | 69 | 112.6 (88.52-143.23) | 112.15, (111.91-112.39) | 7337.24 | 108.29 (88.54) | 6.76 (6.41) |
| Red blood cell count decreased | 68 | 8.29 (6.53-10.52) | 8.26, (8.02-8.5) | 433.02 | 8.24 (6.75) | 3.04 (2.69) |
| Abdominal wall haematoma | 67 | 121.98 (95.52-155.77) | 121.51, (121.26-121.75) | 7707.26 | 116.98 (95.34) | 6.87 (6.51) |
| Coma | 67 | 4.92 (3.87-6.25) | 4.9, (4.66-5.14) | 207.81 | 4.89 (4) | 2.29 (1.94) |
| Haematocrit decreased | 63 | 10.59 (8.27-13.57) | 10.56, (10.31-10.8) | 543.39 | 10.52 (8.55) | 3.4 (3.03) |
| Rectal haemorrhage | 59 | 4.77 (3.69-6.16) | 4.75, (4.5-5.01) | 174.74 | 4.75 (3.83) | 2.25 (1.87) |
| Post procedural haematoma | 59 | 120.79 (93.09-156.73) | 120.37, (120.11-120.63) | 6724.96 | 115.93 (93.23) | 6.86 (6.48) |
| Intra-abdominal haematoma | 56 | 164.92 (126-215.86) | 164.38, (164.12-164.65) | 8638.51 | 156.2 (124.7) | 7.29 (6.89) |
| International normalised ratio increased | 50 | 5.69 (4.31-7.52) | 5.68, (5.4-5.96) | 192.48 | 5.67 (4.49) | 2.5 (2.1) |
| Shock | 49 | 7.77 (5.87-10.29) | 7.75, (7.47-8.03) | 287.39 | 7.73 (6.11) | 2.95 (2.54) |
| Cerebral haematoma | 45 | 46.87 (34.9-62.93) | 46.75, (46.45-47.04) | 1984.87 | 46.07 (36) | 5.53 (5.1) |
| Investigation | 43 | 43.81 (32.41-59.22) | 43.71, (43.41-44.01) | 1769.68 | 43.12 (33.51) | 5.43 (4.99) |
| Haemorrhage intracranial | 43 | 9.66 (7.16-13.04) | 9.64, (9.34-9.94) | 332.03 | 9.61 (7.48) | 3.26 (2.83) |
| Hemiplegia | 43 | 17.79 (13.18-24.01) | 17.75, (17.45-18.04) | 675.73 | 17.65 (13.73) | 4.14 (3.7) |
| **Skin necrosis** | **41** | **27.29 (20.06-37.12)** | **27.22, (26.92-27.53)** | **1026.79** | **27 (20.87)** | **4.75 (4.31)** |
| Hepatic function abnormal | 41 | 3.99 (2.94-5.42) | 3.98, (3.68-4.29) | 91.48 | 3.98 (3.08) | 1.99 (1.55) |
| Platelet count increased | 40 | 10.63 (7.79-14.5) | 10.61, (10.3-10.92) | 346.88 | 10.57 (8.15) | 3.4 (2.95) |
| Retroperitoneal haemorrhage | 40 | 54.13 (39.58-74.02) | 54, (53.69-54.32) | 2045.58 | 53.1 (40.87) | 5.73 (5.28) |
| Hypovolaemic shock | 37 | 27.84 (20.13-38.49) | 27.78, (27.46-28.1) | 946.85 | 27.54 (21) | 4.78 (4.31) |
| Coronary artery thrombosis | 37 | 61.78 (44.61-85.57) | 61.65, (61.32-61.97) | 2164.87 | 60.47 (46.05) | 5.92 (5.44) |
| Coagulopathy | 36 | 7.48 (5.39-10.37) | 7.46, (7.14-7.79) | 201.03 | 7.45 (5.66) | 2.9 (2.42) |
| Activated partial thromboplastin time prolonged | 36 | 24.01 (17.29-33.34) | 23.96, (23.63-24.29) | 786.08 | 23.78 (18.07) | 4.57 (4.1) |
| Altered state of consciousness | 35 | 5.94 (4.26-8.28) | 5.93, (5.6-6.26) | 143.19 | 5.92 (4.48) | 2.57 (2.08) |
| **Aphasia** | **35** | **4 (2.87-5.58)** | **4, (3.67-4.33)** | **78.58** | **3.99 (3.02)** | **2 (1.52)** |
| Haemorrhagic stroke | 34 | 14.75 (10.53-20.67) | 14.73, (14.39-15.06) | 432.98 | 14.66 (11.06) | 3.87 (3.38) |
| Gastric haemorrhage | 33 | 9.62 (6.84-13.55) | 9.61, (9.27-9.95) | 253.77 | 9.58 (7.2) | 3.26 (2.76) |
| Haemarthrosis | 33 | 12.81 (9.09-18.03) | 12.78, (12.44-13.12) | 357 | 12.73 (9.56) | 3.67 (3.17) |
| Intra-abdominal haemorrhage | 33 | 47.87 (33.93-67.54) | 47.78, (47.44-48.13) | 1488.74 | 47.08 (35.3) | 5.56 (5.06) |
| Incision site haemorrhage | 33 | 140.81 (99.31-199.64) | 140.54, (140.19-140.89) | 4374.88 | 134.52 (100.44) | 7.07 (6.57) |
| Gastric ulcer | 32 | 5.81 (4.1-8.21) | 5.8, (5.45-6.14) | 126.8 | 5.79 (4.33) | 2.53 (2.03) |
| C-reactive protein increased | 32 | 3.15 (2.23-4.46) | 3.15, (2.8-3.49) | 46.81 | 3.14 (2.35) | 1.65 (1.15) |
| Purpura | 31 | 12.09 (8.5-17.21) | 12.07, (11.72-12.42) | 313.65 | 12.03 (8.95) | 3.59 (3.08) |
| Hemiparesis | 31 | 6.21 (4.37-8.84) | 6.2, (5.85-6.56) | 135.07 | 6.19 (4.61) | 2.63 (2.12) |
| Haemorrhage subcutaneous | 30 | 28.86 (20.14-41.36) | 28.81, (28.45-29.17) | 798.06 | 28.56 (21.13) | 4.84 (4.31) |
| Ecchymosis | 29 | 14.53 (10.08-20.93) | 14.5, (14.14-14.87) | 362.98 | 14.44 (10.64) | 3.85 (3.32) |
| Upper gastrointestinal haemorrhage | 28 | 5.68 (3.92-8.23) | 5.67, (5.3-6.04) | 107.51 | 5.66 (4.15) | 2.5 (1.96) |
| Mydriasis | 28 | 7.73 (5.33-11.2) | 7.72, (7.35-8.09) | 163.3 | 7.7 (5.64) | 2.94 (2.41) |
| Paralysis | 27 | 6.52 (4.47-9.52) | 6.51, (6.14-6.89) | 125.81 | 6.5 (4.74) | 2.7 (2.15) |
| Haemoptysis | 27 | 3.35 (2.3-4.89) | 3.35, (2.97-3.73) | 44.46 | 3.35 (2.44) | 1.74 (1.2) |
| Compartment syndrome | 27 | 36.06 (24.67-52.71) | 36, (35.62-36.38) | 908.33 | 35.6 (25.91) | 5.15 (4.6) |
| Subcutaneous haematoma | 26 | 69.62 (47.19-102.72) | 69.51, (69.13-69.9) | 1717.47 | 68.02 (49.12) | 6.09 (5.53) |
| Disseminated intravascular coagulation | 26 | 6.28 (4.27-9.23) | 6.27, (5.89-6.66) | 115.06 | 6.26 (4.54) | 2.65 (2.09) |
| Incision site haematoma | 26 | 443.59 (294.07-669.11) | 442.91, (442.5-443.32) | 10037.71 | 387.94 (275.03) | 8.6 (8.01) |
| Rash maculo-papular | 25 | 4.06 (2.74-6.01) | 4.05, (3.66-4.45) | 57.47 | 4.05 (2.92) | 2.02 (1.45) |
| Circulatory collapse | 25 | 4.98 (3.36-7.37) | 4.97, (4.58-5.36) | 79.19 | 4.96 (3.57) | 2.31 (1.74) |
| Prothrombin level decreased | 25 | 87.01 (58.46-129.51) | 86.88, (86.49-87.28) | 2064.89 | 84.56 (60.62) | 6.4 (5.83) |
| Gamma-glutamyltransferase increased | 23 | 3.48 (2.31-5.25) | 3.48, (3.07-3.89) | 40.65 | 3.48 (2.47) | 1.8 (1.21) |
| Wound haemorrhage | 23 | 21.3 (14.13-32.11) | 21.28, (20.87-21.69) | 441.45 | 21.14 (15) | 4.4 (3.81) |
| Brain herniation | 22 | 26.09 (17.14-39.7) | 26.06, (25.64-26.48) | 525.74 | 25.85 (18.19) | 4.69 (4.09) |
| Gastric ulcer haemorrhage | 22 | 13.22 (8.69-20.1) | 13.2, (12.78-13.62) | 247.08 | 13.15 (9.26) | 3.72 (3.11) |
| Product physical issue | 21 | 3.9 (2.54-5.98) | 3.89, (3.47-4.32) | 45.12 | 3.89 (2.72) | 1.96 (1.34) |
| Toxic skin eruption | 20 | 6.99 (4.51-10.84) | 6.98, (6.54-7.42) | 102.28 | 6.97 (4.83) | 2.8 (2.17) |
| Eosinophilia | 20 | 4.12 (2.65-6.38) | 4.11, (3.67-4.55) | 47.07 | 4.11 (2.85) | 2.04 (1.41) |
| Subarachnoid haemorrhage | 20 | 6.77 (4.36-10.5) | 6.76, (6.32-7.2) | 97.95 | 6.75 (4.67) | 2.75 (2.12) |
| Necrosis | 20 | 12.4 (7.99-19.24) | 12.38, (11.94-12.82) | 208.47 | 12.34 (8.54) | 3.63 (2.99) |
| **Anuria** | **20** | **7.85 (5.06-12.18)** | **7.85, (7.41-8.28)** | **119.18** | **7.83 (5.42)** | **2.97 (2.34)** |
| Extradural haematoma | 19 | 49 (31.14-77.12) | 48.95, (48.5-49.4) | 878.69 | 48.21 (32.99) | 5.59 (4.94) |
| Thrombocytosis | 19 | 19.15 (12.19-30.06) | 19.13, (18.67-19.58) | 324.4 | 19.01 (13.03) | 4.25 (3.6) |
| Haemothorax | 19 | 22.65 (14.42-35.57) | 22.62, (22.17-23.08) | 389.9 | 22.47 (15.4) | 4.49 (3.84) |
| Haemodynamic instability | 19 | 9.31 (5.93-14.61) | 9.3, (8.85-9.75) | 140.34 | 9.28 (6.36) | 3.21 (2.57) |
| Traumatic haematoma | 19 | 61.77 (39.21-97.29) | 61.7, (61.25-62.15) | 1112.59 | 60.52 (41.38) | 5.92 (5.27) |
| Gingival bleeding | 18 | 4.95 (3.12-7.86) | 4.95, (4.48-5.41) | 56.58 | 4.94 (3.35) | 2.3 (1.64) |
| Petechiae | 18 | 6.06 (3.82-9.63) | 6.06, (5.6-6.52) | 75.89 | 6.05 (4.11) | 2.6 (1.93) |
| Fibrin d dimer increased | 18 | 17.66 (11.11-28.08) | 17.65, (17.18-18.11) | 281.07 | 17.55 (11.91) | 4.13 (3.47) |
| Spontaneous haematoma | 18 | 85.89 (53.76-137.22) | 85.8, (85.33-86.27) | 1468.23 | 83.53 (56.44) | 6.38 (5.71) |
| Induration | 18 | 34.65 (21.77-55.15) | 34.62, (34.15-35.08) | 581.18 | 34.25 (23.21) | 5.1 (4.43) |
| Post procedural complication | 18 | 3.21 (2.02-5.1) | 3.21, (2.75-3.67) | 27.39 | 3.21 (2.18) | 1.68 (1.02) |
| Brain oedema | 17 | 4.76 (2.96-7.66) | 4.75, (4.28-5.23) | 50.32 | 4.75 (3.19) | 2.25 (1.57) |
| Duodenal ulcer | 17 | 8.45 (5.25-13.6) | 8.44, (7.96-8.92) | 111.21 | 8.42 (5.65) | 3.07 (2.39) |
| Blood lactate dehydrogenase increased | 17 | 3.54 (2.2-5.69) | 3.53, (3.06-4.01) | 30.85 | 3.53 (2.37) | 1.82 (1.14) |
| Leukocytosis | 17 | 3.34 (2.07-5.37) | 3.33, (2.86-3.81) | 27.74 | 3.33 (2.24) | 1.74 (1.05) |
| Hypovolaemia | 16 | 9.48 (5.8-15.49) | 9.47, (8.98-9.96) | 120.91 | 9.45 (6.27) | 3.24 (2.54) |
| Intracranial pressure increased | 16 | 10.14 (6.21-16.57) | 10.13, (9.64-10.62) | 131.31 | 10.1 (6.7) | 3.34 (2.64) |
| Intraventricular haemorrhage | 16 | 21.53 (13.16-35.21) | 21.51, (21.02-22) | 310.75 | 21.37 (14.16) | 4.42 (3.71) |
| Wound secretion | 16 | 13.89 (8.5-22.7) | 13.87, (13.38-14.37) | 190.31 | 13.82 (9.16) | 3.79 (3.09) |
| Duodenal ulcer haemorrhage | 16 | 18.92 (11.57-30.93) | 18.9, (18.41-19.39) | 269.64 | 18.79 (12.45) | 4.23 (3.53) |
| Hepatocellular injury | 15 | 3.53 (2.13-5.86) | 3.53, (3.02-4.04) | 27.18 | 3.53 (2.31) | 1.82 (1.1) |
| Live birth | 15 | 7 (4.21-11.61) | 6.99, (6.48-7.5) | 76.84 | 6.98 (4.57) | 2.8 (2.08) |
| Monoplegia | 15 | 11.89 (7.16-19.75) | 11.88, (11.37-12.39) | 148.91 | 11.84 (7.74) | 3.57 (2.84) |
| Hospice care | 14 | 5.25 (3.11-8.88) | 5.25, (4.73-5.78) | 48.11 | 5.24 (3.38) | 2.39 (1.64) |
| Phlebitis | 14 | 9.32 (5.51-15.75) | 9.31, (8.79-9.84) | 103.57 | 9.29 (5.99) | 3.22 (2.47) |
| Motor dysfunction | 14 | 4.89 (2.9-8.27) | 4.89, (4.37-5.41) | 43.26 | 4.88 (3.15) | 2.29 (1.54) |
| Venous thrombosis | 13 | 11.46 (6.64-19.75) | 11.45, (10.9-11.99) | 123.52 | 11.41 (7.23) | 3.51 (2.74) |
| Haematoma muscle | 13 | 44.32 (25.63-76.65) | 44.29, (43.74-44.84) | 542.38 | 43.68 (27.63) | 5.45 (4.67) |
| Intracranial haematoma | 13 | 79.84 (46.03-138.48) | 79.78, (79.23-80.33) | 986.03 | 77.81 (49.08) | 6.28 (5.5) |
| Embolism | 13 | 5.34 (3.1-9.2) | 5.34, (4.79-5.88) | 45.73 | 5.33 (3.38) | 2.41 (1.64) |
| Postoperative wound complication | 13 | 35.21 (20.38-60.83) | 35.18, (34.63-35.73) | 426.89 | 34.8 (22.02) | 5.12 (4.34) |
| Protein total decreased | 13 | 11.96 (6.94-20.63) | 11.95, (11.41-12.5) | 130.01 | 11.91 (7.55) | 3.57 (2.8) |
| Skin haemorrhage | 13 | 3.52 (2.04-6.06) | 3.52, (2.97-4.06) | 23.38 | 3.51 (2.23) | 1.81 (1.04) |
| Normochromic normocytic anaemia | 12 | 13.58 (7.7-23.94) | 13.57, (13-14.14) | 139.11 | 13.51 (8.41) | 3.76 (2.95) |
| Mouth haemorrhage | 12 | 5.84 (3.31-10.29) | 5.84, (5.27-6.4) | 47.99 | 5.83 (3.63) | 2.54 (1.74) |
| Prothrombin time prolonged | 12 | 6.46 (3.67-11.39) | 6.46, (5.89-7.02) | 55.25 | 6.45 (4.01) | 2.69 (1.89) |
| Contraindication to medical treatment | 12 | 27.32 (15.48-48.24) | 27.31, (26.74-27.87) | 301.48 | 27.08 (16.83) | 4.76 (3.95) |
| Arterial haemorrhage | 12 | 37.89 (21.44-66.96) | 37.86, (37.29-38.43) | 425.52 | 37.42 (23.24) | 5.23 (4.42) |
| Wound infection | 12 | 4.41 (2.5-7.77) | 4.41, (3.84-4.97) | 31.57 | 4.4 (2.74) | 2.14 (1.34) |
| Haematoma infection | 12 | 73.55 (41.48-130.39) | 73.5, (72.92-74.07) | 838.35 | 71.83 (44.48) | 6.17 (5.36) |
| Blood blister | 11 | 12.12 (6.7-21.91) | 12.11, (11.52-12.7) | 111.68 | 12.07 (7.35) | 3.59 (2.76) |
| Catheter site haemorrhage | 11 | 15.94 (8.81-28.84) | 15.93, (15.34-16.53) | 153.2 | 15.86 (9.66) | 3.99 (3.15) |
| Breast haematoma | 11 | 171.57 (93.48-314.87) | 171.46, (170.85-172.07) | 1766.93 | 162.57 (97.81) | 7.34 (6.49) |
| Creatinine renal clearance decreased | 11 | 9.01 (4.98-16.29) | 9.01, (8.41-9.6) | 78.06 | 8.98 (5.47) | 3.17 (2.33) |
| Venous thrombosis limb | 11 | 16.94 (9.36-30.64) | 16.93, (16.33-17.52) | 163.95 | 16.84 (10.26) | 4.07 (3.24) |
| Dermatitis bullous | 11 | 5.41 (3-9.78) | 5.41, (4.82-6) | 39.49 | 5.4 (3.29) | 2.43 (1.6) |
| Nerve compression | 11 | 4.22 (2.34-7.63) | 4.22, (3.63-4.81) | 27 | 4.22 (2.57) | 2.08 (1.24) |
| Brain stem haemorrhage | 10 | 36.31 (19.46-67.74) | 36.29, (35.67-36.91) | 339.24 | 35.89 (21.3) | 5.17 (4.29) |
| Haemorrhagic diathesis | 10 | 9.55 (5.13-17.77) | 9.55, (8.92-10.17) | 76.27 | 9.52 (5.66) | 3.25 (2.38) |
| International normalised ratio decreased | 10 | 7.93 (4.26-14.75) | 7.93, (7.31-8.55) | 60.38 | 7.91 (4.7) | 2.98 (2.11) |
| Haemorrhagic disorder | 10 | 31.6 (16.95-58.93) | 31.58, (30.96-32.21) | 293.2 | 31.28 (18.57) | 4.97 (4.09) |
| Pulmonary haemorrhage | 10 | 4.31 (2.32-8.02) | 4.31, (3.69-4.93) | 25.4 | 4.31 (2.56) | 2.11 (1.24) |
| Hypothermia | 10 | 3.49 (1.88-6.5) | 3.49, (2.87-4.11) | 17.76 | 3.49 (2.08) | 1.8 (0.93) |
| Procedural complication | 9 | 3.92 (2.04-7.53) | 3.92, (3.26-4.57) | 19.51 | 3.91 (2.26) | 1.97 (1.05) |
| Cerebellar haematoma | 9 | 104.35 (53.7-202.78) | 104.3, (103.64-104.96) | 890.98 | 100.96 (57.91) | 6.66 (5.73) |
| Arterial thrombosis | 9 | 15.89 (8.25-30.59) | 15.88, (15.22-16.53) | 124.84 | 15.8 (9.13) | 3.98 (3.07) |
| Pelvic haematoma | 9 | 72.35 (37.35-140.13) | 72.31, (71.65-72.97) | 618.58 | 70.69 (40.66) | 6.14 (5.22) |
| Peritoneal haematoma | 9 | 221.04 (112.4-434.67) | 220.92, (220.24-221.6) | 1839.93 | 206.37 (117.19) | 7.69 (6.74) |
| Small intestinal haemorrhage | 9 | 15.53 (8.07-29.91) | 15.53, (14.87-16.18) | 121.72 | 15.45 (8.93) | 3.95 (3.03) |
| Adrenal haemorrhage | 9 | 60.76 (31.41-117.54) | 60.73, (60.07-61.39) | 518.61 | 59.59 (34.31) | 5.9 (4.97) |
| Coagulation time prolonged | 9 | 19.7 (10.23-37.94) | 19.69, (19.03-20.34) | 158.66 | 19.57 (11.31) | 4.29 (3.37) |
| Hyperthermia | 9 | 4.16 (2.16-7.99) | 4.15, (3.5-4.81) | 21.53 | 4.15 (2.4) | 2.05 (1.14) |
| Subdural haemorrhage | 8 | 9.93 (4.96-19.88) | 9.92, (9.23-10.62) | 64 | 9.9 (5.54) | 3.31 (2.34) |
| Cardiac tamponade | 8 | 5.87 (2.93-11.75) | 5.87, (5.17-6.56) | 32.25 | 5.86 (3.28) | 2.55 (1.59) |
| Splenic haemorrhage | 8 | 54.96 (27.31-110.58) | 54.93, (54.23-55.63) | 416.27 | 54 (30.08) | 5.75 (4.78) |
| Joint effusion | 8 | 3.97 (1.99-7.95) | 3.97, (3.28-4.67) | 17.77 | 3.97 (2.22) | 1.99 (1.03) |
| Post procedural drainage | 8 | 213.26 (104.18-436.56) | 213.16, (212.44-213.87) | 1581.18 | 199.58 (109.59) | 7.64 (6.65) |
| Cerebral ischaemia | 8 | 5.12 (2.56-10.24) | 5.12, (4.42-5.81) | 26.45 | 5.11 (2.86) | 2.35 (1.39) |
| Effusion | 8 | 20.07 (10.01-40.23) | 20.06, (19.37-20.76) | 143.99 | 19.94 (11.15) | 4.32 (3.35) |
| Wound complication | 8 | 8.09 (4.04-16.2) | 8.09, (7.4-8.78) | 49.58 | 8.07 (4.52) | 3.01 (2.05) |
| Brain neoplasm malignant | 7 | 5.22 (2.49-10.95) | 5.22, (4.47-5.96) | 23.81 | 5.21 (2.8) | 2.38 (1.36) |
| Paraplegia | 7 | 8.5 (4.05-17.85) | 8.49, (7.75-9.24) | 46.16 | 8.47 (4.55) | 3.08 (2.06) |
| Pericardial haemorrhage | 7 | 10.77 (5.13-22.62) | 10.77, (10.02-11.51) | 61.8 | 10.73 (5.77) | 3.42 (2.4) |
| Sciatic nerve palsy | 7 | 295.01 (135.89-640.48) | 294.89, (294.12-295.67) | 1873.08 | 269.49 (140.88) | 8.07 (7.01) |
| Thrombophlebitis | 7 | 6.71 (3.2-14.09) | 6.71, (5.97-7.45) | 33.94 | 6.7 (3.6) | 2.74 (1.72) |
| Incorrect dosage administered | 6 | 4.38 (1.96-9.75) | 4.38, (3.57-5.18) | 15.6 | 4.37 (2.24) | 2.13 (1.04) |
| Anticoagulation drug level above therapeutic | 6 | 13.59 (6.09-30.3) | 13.58, (12.78-14.39) | 69.64 | 13.53 (6.92) | 3.76 (2.66) |
| Brain death | 6 | 6.72 (3.01-14.96) | 6.71, (5.91-7.51) | 29.12 | 6.7 (3.43) | 2.74 (1.65) |
| Abdominal wall haemorrhage | 6 | 82.07 (36.48-184.62) | 82.04, (81.23-82.85) | 467.98 | 79.96 (40.57) | 6.32 (5.21) |
| Antiphospholipid syndrome | 6 | 13.63 (6.11-30.39) | 13.62, (12.82-14.42) | 69.88 | 13.57 (6.94) | 3.76 (2.67) |
| Bullous haemorrhagic dermatosis | 6 | 165.58 (72.84-376.44) | 165.53, (164.71-166.35) | 931.73 | 157.23 (79.09) | 7.3 (6.17) |
| Monoparesis | 6 | 11.35 (5.09-25.31) | 11.35, (10.55-12.15) | 56.42 | 11.31 (5.78) | 3.5 (2.41) |
| Post procedural swelling | 6 | 20.34 (9.11-45.39) | 20.33, (19.53-21.13) | 109.57 | 20.21 (10.32) | 4.34 (3.24) |
| Lung cancer metastatic | 6 | 7.01 (3.15-15.62) | 7.01, (6.21-7.81) | 30.84 | 6.99 (3.58) | 2.81 (1.71) |
| Extensor plantar response | 6 | 21.43 (9.6-47.85) | 21.43, (20.62-22.23) | 116.04 | 21.29 (10.87) | 4.41 (3.32) |
| Conjunctival pallor | 6 | 118.42 (52.4-267.64) | 118.38, (117.57-119.2) | 672.8 | 114.09 (57.67) | 6.83 (5.72) |
| Abdominal mass | 5 | 6.84 (2.85-16.46) | 6.84, (5.97-7.72) | 24.89 | 6.83 (3.28) | 2.77 (1.59) |
| Vena cava thrombosis | 5 | 10.37 (4.31-24.96) | 10.37, (9.49-11.25) | 42.2 | 10.34 (4.96) | 3.37 (2.19) |
| Prurigo | 5 | 17.86 (7.41-43.02) | 17.85, (16.98-18.73) | 79.1 | 17.76 (8.51) | 4.15 (2.97) |
| Oesophageal candidiasis | 5 | 4.61 (1.92-11.07) | 4.6, (3.73-5.48) | 14.09 | 4.6 (2.21) | 2.2 (1.02) |
| Anastomotic haemorrhage | 5 | 91.18 (37.47-221.9) | 91.15, (90.26-92.04) | 433.15 | 88.59 (42.09) | 6.47 (5.27) |
| Hemianopia | 5 | 17.3 (7.18-41.68) | 17.3, (16.42-18.18) | 76.37 | 17.21 (8.25) | 4.11 (2.92) |
| Splenic rupture | 5 | 10.26 (4.27-24.7) | 10.26, (9.38-11.14) | 41.66 | 10.23 (4.91) | 3.35 (2.17) |
| Hypocoagulable state | 5 | 17.96 (7.46-43.27) | 17.96, (17.08-18.84) | 79.61 | 17.86 (8.56) | 4.16 (2.98) |
| Hepatic haematoma | 5 | 36.26 (15.01-87.57) | 36.25, (35.37-37.13) | 169.41 | 35.84 (17.14) | 5.16 (3.98) |
| Pyelocaliectasis | 5 | 19.32 (8.02-46.55) | 19.31, (18.44-20.19) | 86.3 | 19.2 (9.2) | 4.26 (3.08) |
| Renal haemorrhage | 5 | 8.43 (3.5-20.27) | 8.43, (7.55-9.3) | 32.63 | 8.41 (4.03) | 3.07 (1.89) |
| Superinfection | 5 | 11.67 (4.85-28.09) | 11.67, (10.79-12.55) | 48.58 | 11.63 (5.58) | 3.54 (2.36) |
| Portal vein thrombosis | 5 | 5.54 (2.3-13.31) | 5.54, (4.66-6.41) | 18.55 | 5.53 (2.65) | 2.47 (1.29) |
| Anaemia postoperative | 5 | 12.25 (5.09-29.48) | 12.24, (11.37-13.12) | 51.43 | 12.2 (5.85) | 3.61 (2.43) |
| Gastrointestinal ulcer | 5 | 7.79 (3.24-18.73) | 7.79, (6.91-8.66) | 29.5 | 7.77 (3.73) | 2.96 (1.78) |
| Bleeding time prolonged | 5 | 13.86 (5.76-33.37) | 13.86, (12.98-14.73) | 59.37 | 13.8 (6.62) | 3.79 (2.61) |
| Wound dehiscence | 5 | 5.08 (2.11-12.21) | 5.08, (4.2-5.95) | 16.34 | 5.07 (2.43) | 2.34 (1.16) |
| Postoperative thrombosis | 5 | 29.59 (12.26-71.39) | 29.58, (28.7-30.46) | 136.76 | 29.31 (14.03) | 4.87 (3.69) |
| Puncture site haemorrhage | 5 | 24.67 (10.23-59.48) | 24.66, (23.78-25.54) | 112.63 | 24.48 (11.72) | 4.61 (3.43) |
| Coagulation factor decreased | 5 | 63.12 (26.04-153.02) | 63.11, (62.22-63.99) | 299.55 | 61.87 (29.49) | 5.95 (4.76) |
| Prothrombin time shortened | 5 | 11.88 (4.94-28.6) | 11.88, (11-12.76) | 49.64 | 11.84 (5.68) | 3.57 (2.38) |
| Paresis | 5 | 7.54 (3.13-18.13) | 7.54, (6.66-8.41) | 28.28 | 7.52 (3.61) | 2.91 (1.73) |
| Bilirubin conjugated increased | 5 | 7.43 (3.09-17.88) | 7.43, (6.56-8.31) | 27.77 | 7.42 (3.56) | 2.89 (1.71) |
| Livedo reticularis | 5 | 8.48 (3.53-20.41) | 8.48, (7.6-9.36) | 32.9 | 8.46 (4.06) | 3.08 (1.9) |
| Pancreatic carcinoma metastatic | 5 | 5.9 (2.45-14.19) | 5.9, (5.02-6.77) | 20.3 | 5.89 (2.83) | 2.56 (1.38) |
| Neurological decompensation | 5 | 8.08 (3.36-19.43) | 8.08, (7.2-8.95) | 30.92 | 8.06 (3.87) | 3.01 (1.83) |
| Chemotherapy | 5 | 5.68 (2.36-13.66) | 5.68, (4.8-6.56) | 19.24 | 5.67 (2.72) | 2.5 (1.32) |
| Splenic haematoma | 4 | 49.89 (18.58-133.99) | 49.88, (48.89-50.87) | 188.58 | 49.11 (21.49) | 5.62 (4.31) |
| Gastrointestinal necrosis | 4 | 4.75 (1.78-12.68) | 4.75, (3.77-5.73) | 11.84 | 4.75 (2.09) | 2.25 (0.96) |
| Tumour haemorrhage | 4 | 5.06 (1.9-13.49) | 5.06, (4.08-6.04) | 13 | 5.05 (2.22) | 2.34 (1.04) |
| Product name confusion | 4 | 11.12 (4.16-29.67) | 11.11, (10.13-12.1) | 36.69 | 11.08 (4.87) | 3.47 (2.18) |
| Hypofibrinogenaemia | 4 | 14.88 (5.57-39.75) | 14.88, (13.9-15.86) | 51.54 | 14.81 (6.51) | 3.89 (2.59) |
| Renal haematoma | 4 | 27.06 (10.11-72.4) | 27.05, (26.07-28.03) | 99.48 | 26.83 (11.77) | 4.75 (3.45) |
| Cerebellar haemorrhage | 4 | 9.31 (3.49-24.84) | 9.31, (8.32-10.29) | 29.57 | 9.28 (4.08) | 3.21 (1.92) |
| Anticoagulation drug level below therapeutic | 4 | 17.47 (6.54-46.68) | 17.46, (16.48-18.45) | 61.74 | 17.37 (7.63) | 4.12 (2.82) |
| Jugular vein thrombosis | 4 | 7.91 (2.97-21.11) | 7.91, (6.93-8.89) | 24.09 | 7.89 (3.47) | 2.98 (1.69) |
| Thrombosis with thrombocytopenia syndrome | 4 | 566.94 (195.35-1645.38) | 566.8, (565.74-567.87) | 1911.65 | 479.76 (196.72) | 8.91 (7.5) |
| Hypersensitivity myocarditis | 4 | 138.58 (50.9-377.33) | 138.55, (137.55-139.55) | 522.99 | 132.7 (57.4) | 7.05 (5.73) |
| Ear haemorrhage | 4 | 6.89 (2.58-18.39) | 6.89, (5.91-7.87) | 20.11 | 6.88 (3.03) | 2.78 (1.49) |
| Soft tissue haemorrhage | 4 | 36.68 (13.69-98.32) | 36.68, (35.69-37.66) | 137.2 | 36.26 (15.89) | 5.18 (3.88) |
| Electrocardiogram st segment depression | 4 | 6.11 (2.29-16.3) | 6.11, (5.13-7.09) | 17.06 | 6.1 (2.68) | 2.61 (1.32) |
| Hepatic haemorrhage | 4 | 16.54 (6.19-44.19) | 16.54, (15.56-17.52) | 58.09 | 16.46 (7.23) | 4.04 (2.75) |
| Urinary bladder haemorrhage | 4 | 6.59 (2.47-17.57) | 6.58, (5.6-7.56) | 18.9 | 6.57 (2.89) | 2.72 (1.42) |
| Incisional drainage | 4 | 29.7 (11.09-79.5) | 29.69, (28.71-30.67) | 109.85 | 29.42 (12.91) | 4.88 (3.58) |
| Hyporeflexia | 4 | 6.98 (2.62-18.62) | 6.98, (6-7.96) | 20.44 | 6.96 (3.06) | 2.8 (1.51) |
| Hyperlactacidaemia | 4 | 5.77 (2.16-15.4) | 5.77, (4.79-6.75) | 15.76 | 5.76 (2.54) | 2.53 (1.24) |
| Hypercapnia | 4 | 4.77 (1.79-12.71) | 4.76, (3.78-5.75) | 11.88 | 4.76 (2.09) | 2.25 (0.96) |
| Varicose vein ruptured | 4 | 43.92 (16.37-117.84) | 43.91, (42.92-44.89) | 165.39 | 43.31 (18.96) | 5.44 (4.13) |
| Mucosal haemorrhage | 4 | 12.12 (4.54-32.36) | 12.12, (11.14-13.1) | 40.65 | 12.08 (5.31) | 3.59 (2.3) |
| Emergency care examination | 4 | 22.51 (8.42-60.21) | 22.51, (21.53-23.49) | 81.62 | 22.35 (9.82) | 4.48 (3.19) |
| Hemianopia homonymous | 3 | 14.19 (4.57-44.13) | 14.19, (13.06-15.33) | 36.62 | 14.13 (5.47) | 3.82 (2.37) |
| Vascular rupture | 3 | 6.64 (2.14-20.61) | 6.64, (5.5-7.77) | 14.33 | 6.63 (2.57) | 2.73 (1.28) |
| Gastroduodenal ulcer | 3 | 25.28 (8.12-78.75) | 25.28, (24.14-26.41) | 69.39 | 25.08 (9.69) | 4.65 (3.2) |
| Tracheal haemorrhage | 3 | 29.6 (9.49-92.28) | 29.6, (28.46-30.73) | 82.11 | 29.33 (11.33) | 4.87 (3.42) |
| Bladder tamponade | 3 | 35.3 (11.31-110.16) | 35.29, (34.15-36.43) | 98.84 | 34.91 (13.47) | 5.13 (3.67) |
| Acute abdomen | 3 | 6.95 (2.24-21.59) | 6.95, (5.82-8.09) | 15.26 | 6.94 (2.69) | 2.79 (1.35) |
| Bladder dysfunction | 3 | 10.34 (3.33-32.11) | 10.33, (9.2-11.47) | 25.21 | 10.3 (3.99) | 3.37 (1.92) |
| Rash scarlatiniform | 3 | 47.48 (15.18-148.51) | 47.47, (46.33-48.61) | 134.44 | 46.78 (18.02) | 5.55 (4.09) |
| Respiratory tract haemorrhage | 3 | 16.98 (5.46-52.8) | 16.97, (15.84-18.11) | 44.85 | 16.89 (6.53) | 4.08 (2.63) |
| Bloody discharge | 3 | 9.15 (2.95-28.43) | 9.15, (8.02-10.28) | 21.72 | 9.13 (3.54) | 3.19 (1.74) |
| Blood loss anaemia | 3 | 5.38 (1.73-16.7) | 5.38, (4.25-6.51) | 10.67 | 5.37 (2.08) | 2.43 (0.98) |
| Spinal cord haemorrhage | 3 | 26.57 (8.53-82.8) | 26.57, (25.43-27.71) | 73.2 | 26.35 (10.18) | 4.72 (3.27) |
| Oesophageal haemorrhage | 3 | 7.76 (2.5-24.08) | 7.75, (6.62-8.89) | 17.61 | 7.74 (3) | 2.95 (1.51) |
| Urethral haemorrhage | 3 | 17.62 (5.66-54.8) | 17.61, (16.48-18.75) | 46.74 | 17.52 (6.78) | 4.13 (2.68) |
| Ureteric compression | 3 | 137.56 (43.28-437.22) | 137.53, (136.38-138.69) | 389.44 | 131.76 (50.07) | 7.04 (5.56) |
| Cross sensitivity reaction | 3 | 16.76 (5.39-52.14) | 16.76, (15.63-17.89) | 44.22 | 16.68 (6.45) | 4.06 (2.61) |
| Pancreatitis haemorrhagic | 3 | 21.31 (6.84-66.33) | 21.3, (20.17-22.44) | 57.66 | 21.17 (8.18) | 4.4 (2.95) |
| Product monitoring error | 3 | 6.84 (2.2-21.23) | 6.84, (5.7-7.97) | 14.92 | 6.82 (2.64) | 2.77 (1.33) |
| Peripheral artery thrombosis | 3 | 7.45 (2.4-23.12) | 7.45, (6.31-8.58) | 16.7 | 7.43 (2.88) | 2.89 (1.45) |
| Procedural site reaction | 3 | 19.57 (6.29-60.9) | 19.57, (18.43-20.7) | 52.52 | 19.45 (7.52) | 4.28 (2.83) |
| Wound drainage | 3 | 12.29 (3.95-38.2) | 12.29, (11.16-13.42) | 30.99 | 12.25 (4.74) | 3.61 (2.17) |
| Blood fibrinogen increased | 3 | 12.36 (3.98-38.4) | 12.35, (11.22-13.49) | 31.18 | 12.31 (4.77) | 3.62 (2.18) |
| Marrow hyperplasia | 3 | 14.82 (4.77-46.09) | 14.82, (13.69-15.96) | 38.48 | 14.76 (5.71) | 3.88 (2.44) |
| Cardiac procedure complication | 3 | 16.21 (5.21-50.42) | 16.21, (15.07-17.34) | 42.59 | 16.13 (6.24) | 4.01 (2.56) |
| Aortic aneurysm rupture | 3 | 10.27 (3.3-31.9) | 10.27, (9.13-11.4) | 25.01 | 10.24 (3.96) | 3.36 (1.91) |
| Catheter site discharge | 3 | 10.2 (3.28-31.69) | 10.2, (9.07-11.33) | 24.81 | 10.17 (3.94) | 3.35 (1.9) |
| Colonic haematoma | 3 | 126.4 (39.85-400.97) | 126.38, (125.23-127.54) | 358.63 | 121.5 (46.24) | 6.92 (5.45) |
| Decerebration | 3 | 83.52 (26.53-262.91) | 83.5, (82.36-84.65) | 238.16 | 81.35 (31.16) | 6.35 (4.88) |
| Mechanical ventilation | 3 | 5.31 (1.71-16.47) | 5.3, (4.17-6.44) | 10.46 | 5.3 (2.05) | 2.41 (0.96) |
| Skin graft | 3 | 11.03 (3.55-34.27) | 11.03, (9.9-12.16) | 27.26 | 10.99 (4.26) | 3.46 (2.01) |
| Surgical procedure repeated | 3 | 14.3 (4.6-44.47) | 14.3, (13.17-15.43) | 36.94 | 14.24 (5.51) | 3.83 (2.38) |
| Spinal epidural haemorrhage | 3 | 139.61 (43.91-443.91) | 139.59, (138.43-140.74) | 395.09 | 133.65 (50.77) | 7.06 (5.58) |
| **Conjunctival discolouration** | **3** | **87.42 (27.75-275.39)** | **87.4, (86.26-88.55)** | **249.26** | **85.05 (32.56)** | **6.41 (4.94)** |
| Meningorrhagia | 3 | 38.65 (12.38-120.7) | 38.65, (37.51-39.78) | 108.67 | 38.18 (14.73) | 5.25 (3.8) |
| Abdominal symptom | 3 | 13.21 (4.25-41.07) | 13.21, (12.08-14.34) | 33.71 | 13.16 (5.09) | 3.72 (2.27) |
| Puncture site pain | 3 | 53.45 (17.07-167.36) | 53.44, (52.3-54.58) | 151.78 | 52.56 (20.22) | 5.72 (4.26) |
| Rectal ulcer haemorrhage | 3 | 42.91 (13.73-134.09) | 42.9, (41.76-44.04) | 121.1 | 42.33 (16.32) | 5.4 (3.95) |
| Fibrin degradation products increased | 3 | 32.59 (10.45-101.66) | 32.59, (31.45-33.72) | 90.9 | 32.26 (12.45) | 5.01 (3.56) |
| Heparin-induced thrombocytopenia test | 3 | 143.91 (45.22-457.92) | 143.88, (142.72-145.04) | 406.89 | 137.58 (52.23) | 7.1 (5.62) |
| Cell death | 3 | 5.26 (1.7-16.34) | 5.26, (4.13-6.4) | 10.34 | 5.26 (2.04) | 2.39 (0.95) |
| Application site nodule | 3 | 76.67 (24.39-241.06) | 76.66, (75.51-77.8) | 218.64 | 74.84 (28.7) | 6.23 (4.76) |
| Post procedural pulmonary embolism | 3 | 44.76 (14.32-139.91) | 44.75, (43.61-45.89) | 126.49 | 44.13 (17) | 5.46 (4.01) |

Bolded terms represent potential novel signals of adverse events at the PT level for fondaparinux sodium; ROR, reporting odds ratio; PRR, proportional reporting ratio; EBGM, empirical Bayesian geometric mean; EBGM05, the lower limit of the 95% CI of EBGM; IC, information component; IC025, the lower limit of the 95% CI of the IC; CI, confidence interval; χ2, chi-squared; PT, preferred term.

Supplementary Table 5:

List of 68 PTs that met the positivity criteria for at least one of the four disproportionality algorithms in the JADER database.

| PT | Case  numbers | ROR  (95%CI) | PRR  (95%CI) | χ^2^ | EBGM  (EBGM_05_) | IC  (IC_025_) |
| --- | --- | --- | --- | --- | --- | --- |
| Post procedural haemorrhage | 180 | 216.99（183.43-256.69) | 183.07（155.05-216.15) | 28720 | 161.28（136.34) | 7.33（5.66) |
| Haemorrhage | 71 | 30.58（23.99-38.97) | 28.74（22.56-36.62) | 1865.71 | 28.16（22.1) | 4.82（3.15) |
| Haemoglobin decreased | 55 | 15.97（12.16-20.97) | 15.25（11.62-20.02) | 726.53 | 15.09（11.49) | 3.92（2.25) |
| Anaemia | 54 | 4.81（3.66-6.33) | 4.63（3.52-6.09) | 154.81 | 4.62（3.51) | 2.21（0.54) |
| Post procedural haematoma | 51 | 845.51（594.52-1202.46) | 807.93（568.22-1148.75) | 25691.28 | 505.33（355.32) | 8.98（7.29) |
| Haemorrhage subcutaneous | 41 | 20.78（15.18-28.45) | 20.08（14.67-27.48) | 733.56 | 19.8（14.46) | 4.31（2.64) |
| Gastrointestinal haemorrhage | 34 | 9.01（6.4-12.69) | 8.77（6.23-12.35) | 233.36 | 8.72（6.19) | 3.12（1.46) |
| Hepatic function abnormal | 30 | 2.21（1.53-3.17) | 2.17（1.51-3.12) | 19.22 | 2.17（1.51) | 1.12（-0.55) |
| Haematoma | 22 | 39.67（25.86-60.85) | 38.93（25.38-59.71) | 790.54 | 37.86（24.68) | 5.24（3.57) |
| Melaena | 21 | 9.02（5.85-13.91) | 8.87（5.75-13.68) | 146.04 | 8.82（5.72) | 3.14（1.47) |
| Haematemesis | 19 | 22.53（14.26-35.58) | 22.17（14.04-35.01) | 378.16 | 21.83（13.82) | 4.45（2.78) |
| Intra-abdominal haemorrhage | 18 | 49.63（30.9-79.72) | 48.87（30.42-78.49) | 814.68 | 47.19（29.38) | 5.56（3.89) |
| Gastric haemorrhage | 15 | 30.26（18.08-50.65) | 29.88（17.85-50) | 409.78 | 29.25（17.48) | 4.87（3.2) |
| Subcutaneous haematoma | 14 | 51.57（30.15-88.21) | 50.95（29.79-87.15) | 660.75 | 49.13（28.72) | 5.62（3.94) |
| Shock haemorrhagic | 13 | 13.39（7.73-23.2) | 13.25（7.65-22.95) | 145.94 | 13.13（7.58) | 3.72（2.05) |
| Cerebral haemorrhage | 13 | 2.57（1.49-4.45) | 2.55（1.48-4.41) | 12.33 | 2.55（1.48) | 1.35（-0.32) |
| Upper gastrointestinal haemorrhage | 11 | 15.19（8.36-27.6) | 15.05（8.29-27.35) | 142.81 | 14.9（8.2) | 3.9（2.23) |
| Platelet count increased | 11 | 85.94（46.6-158.51) | 85.13（46.16-157) | 860.25 | 80.12（43.44) | 6.32（4.64) |
| Peripheral swelling | 10 | 23.7（12.65-44.4) | 23.5（12.54-44.03) | 211.81 | 23.11（12.34) | 4.53（2.86) |
| Subdural haematoma | 9 | 5.48（2.84-10.57) | 5.44（2.82-10.5) | 32.55 | 5.42（2.81) | 2.44（0.77) |
| Haematoma muscle | 9 | 23（11.87-44.55) | 22.82（11.78-44.22) | 184.73 | 22.46（11.59) | 4.49（2.82) |
| Shock | 9 | 2.65（1.38-5.12) | 2.64（1.37-5.09) | 9.19 | 2.64（1.37) | 1.4（-0.27) |
| Extradural haematoma | 8 | 26.26（13.01-52.99) | 26.08（12.93-52.63) | 189.36 | 25.61（12.69) | 4.68（3) |
| Gastric ulcer haemorrhage | 7 | 5.73（2.72-12.06) | 5.7（2.71-12) | 27.03 | 5.68（2.7) | 2.51（0.84) |
| Deep vein thrombosis | 7 | 2.76（1.31-5.81) | 2.75（1.31-5.79) | 7.81 | 2.75（1.31) | 1.46（-0.21) |
| Heparin-induced thrombocytopenia | 7 | 4.97（2.36-10.47) | 4.95（2.35-10.42) | 22 | 4.93（2.34) | 2.3（0.63) |
| Intra-abdominal haematoma | 7 | 152.96（69.86-334.9) | 152.03（69.44-332.86) | 943.75 | 136.71（62.44) | 7.09（5.39) |
| Thrombosis | 7 | 5.77（2.74-12.16) | 5.74（2.73-12.1) | 27.34 | 5.72（2.72) | 2.52（0.85) |
| Alanine aminotransferase increased | 7 | 2.35（1.12-4.94) | 2.34（1.11-4.92) | 5.37 | 2.34（1.11) | 1.22（-0.44) |
| Retroperitoneal haematoma | 7 | 33.39（15.74-70.83) | 33.19（15.64-70.41) | 213.32 | 32.42（15.28) | 5.02（3.34) |
| Haemarthrosis | 6 | 18.67（8.32-41.86) | 18.57（8.28-41.66) | 98.43 | 18.33（8.17) | 4.2（2.52) |
| Subarachnoid haemorrhage | 6 | 4.85（2.17-10.84) | 4.83（2.16-10.79) | 18.19 | 4.82（2.16) | 2.27（0.6) |
| Small intestinal haemorrhage | 5 | 7.9（3.27-19.05) | 7.87（3.26-18.98) | 29.81 | 7.83（3.24) | 2.97（1.3) |
| Gastric ulcer | 5 | 4.01（1.66-9.66) | 4（1.66-9.63) | 11.21 | 3.99（1.65) | 2（0.33) |
| Swelling | 5 | 12.83（5.31-31.01) | 12.78（5.29-30.88) | 53.78 | 12.66（5.24) | 3.66（1.99) |
| Anastomotic haemorrhage | 5 | 82.46（33.37-203.79) | 82.11（33.22-202.91) | 377.59 | 77.45（31.34) | 6.28（4.58) |
| Abdominal wall haematoma | 5 | 30.46（12.53-74.04) | 30.33（12.48-73.72) | 138.7 | 29.68（12.21) | 4.89（3.21) |
| Abdominal pain | 5 | 2.67（1.11-6.44) | 2.66（1.11-6.42) | 5.2 | 2.66（1.1) | 1.41（-0.26) |
| C-reactive protein increased | 5 | 2.79（1.16-6.72) | 2.78（1.15-6.7) | 5.7 | 2.78（1.15) | 1.47（-0.2) |
| Duodenal ulcer haemorrhage | 4 | 7.65（2.86-20.48) | 7.63（2.85-20.42) | 22.92 | 7.59（2.84) | 2.92（1.25) |
| Muscle haemorrhage | 4 | 13.15（4.9-35.26) | 13.11（4.89-35.14) | 44.31 | 12.99（4.84) | 3.7（2.03) |
| Gamma-glutamyltransferase increased | 4 | 4.35（1.63-11.62) | 4.34（1.62-11.59) | 10.24 | 4.33（1.62) | 2.11（0.44) |
| Pain in extremity | 4 | 3.07（1.15-8.2) | 3.06（1.15-8.18) | 5.55 | 3.06（1.14) | 1.61（-0.06) |
| Haematocrit decreased | 4 | 20.87（7.76-56.11) | 20.8（7.73-55.92) | 74.24 | 20.5（7.62) | 4.36（2.68) |
| Red blood cell count decreased | 4 | 7.38（2.76-19.75) | 7.36（2.75-19.69) | 21.87 | 7.32（2.74) | 2.87（1.2) |
| Cerebellar haemorrhage | 3 | 5.58（1.79-17.38) | 5.57（1.79-17.34) | 11.21 | 5.55（1.78) | 2.47（0.8) |
| Postoperative wound infection | 3 | 18.24（5.83-57.08) | 18.2（5.82-56.94) | 48.11 | 17.97（5.74) | 4.17（2.49) |
| Lower gastrointestinal haemorrhage | 3 | 5.86（1.88-18.24) | 5.85（1.88-18.2) | 12 | 5.83（1.87) | 2.54（0.87) |
| Impaired healing | 3 | 9.27（2.97-28.88) | 9.24（2.97-28.82) | 21.91 | 9.19（2.95) | 3.2（1.53) |
| Brain stem haemorrhage | 3 | 9.9（3.18-30.87) | 9.88（3.17-30.8) | 23.77 | 9.81（3.15) | 3.29（1.62) |
| Blood loss anaemia | 3 | 14.21（4.55-44.38) | 14.17（4.54-44.28) | 36.36 | 14.04（4.49) | 3.81（2.13) |
| Wound haemorrhage | 3 | 37.85（12-119.4) | 37.75（11.97-119.09) | 104.42 | 36.75（11.65) | 5.2（3.51) |
| Blood alkaline phosphatase increased | 3 | 4.21（1.35-13.09) | 4.2（1.35-13.06) | 7.29 | 4.19（1.35) | 2.07（0.4) |
| Protein total decreased | 3 | 24.69（7.87-77.47) | 24.63（7.85-77.28) | 66.8 | 24.21（7.72) | 4.6（2.91) |
| Retroperitoneal haemorrhage | 3 | 20.35（6.5-63.73) | 20.3（6.48-63.57) | 54.24 | 20.01（6.39) | 4.32（2.64) |
| Hepatic haemorrhage | 2 | 33.3（8.18-135.62) | 33.25（8.17-135.39) | 61.05 | 32.47（7.97) | 5.02（3.32) |
| Postoperative wound complication | 2 | 158.69（36.62-687.67) | 158.42（36.56-686.47) | 279.92 | 141.85（32.73) | 7.15（5.36) |
| Sciatic nerve palsy | 2 | 674.45（123.41-3686) | 673.27（123.19-3679.57) | 895.04 | 449.18（82.19) | 8.81（6.82) |
| Skin necrosis | 2 | 9.95（2.47-40.05) | 9.94（2.47-39.99) | 15.96 | 9.87（2.45) | 3.3（1.63) |
| Post procedural swelling | 2 | 2697.8（244.45-29774.05) | 2693.1（244.02-29722.08) | 1794.07 | 898.37（81.4) | 9.81（7.61) |
| Catheter site haemorrhage | 2 | 64.23（15.53-265.66) | 64.12（15.5-265.2) | 118.63 | 61.25（14.81) | 5.94（4.21) |
| Wound decomposition | 2 | 168.61（38.72-734.17) | 168.32（38.66-732.89) | 295.69 | 149.73（34.39) | 7.23（5.43) |
| Haemorrhagic ascites | 2 | 48.17（11.74-197.64) | 48.09（11.72-197.3) | 89.05 | 46.47（11.33) | 5.54（3.83) |
| Compartment syndrome | 2 | 26.98（6.65-109.49) | 26.93（6.64-109.31) | 48.96 | 26.42（6.51) | 4.72（3.03) |
| Vaginal haematoma | 2 | 385.4（79.97-1857.23) | 384.73（79.84-1854) | 595.36 | 299.46（62.14) | 8.23（6.32) |
| Pelvic haematoma | 2 | 299.75（64.69-1388.89) | 299.23（64.58-1386.47) | 486.39 | 245.01（52.88) | 7.94（6.07) |
| Arterial haemorrhage | 2 | 9.67（2.4-38.9) | 9.65（2.4-38.84) | 15.4 | 9.59（2.38) | 3.26（1.58) |
| Gastrostomy | 2 | 50.9（12.39-209.12) | 50.81（12.37-208.76) | 94.12 | 49（11.93) | 5.61（3.9) |

ROR, reporting odds ratio; PRR, proportional reporting ratio; EBGM, empirical Bayesian geometric mean; EBGM05, the lower limit of the 95% CI of EBGM; IC, information component; IC025, the lower limit of the 95% CI of the IC; CI, confidence interval; χ2, chi-squared; PT, preferred term.

Supplementary Table 6:

Top 50 most frequent AEs for fondaparinux sodium at the preferred term (PT) level in males from FAERS data.

| PT | Case numbers | ROR(95%CI) | PRR(95%CI) | χ2 | EBGM(EBGM05) | IC(IC025) |
| --- | --- | --- | --- | --- | --- | --- |
| Anaemia* | 166 | 7.71 (6.6, 8.99) | 7.52 (7.37, 7.67) | 939.91 | 7.51 (6.6) | 2.91 (2.68) |
| Haematoma* | 158 | 52.45 (44.73, 61.51) | 51.12 (50.97, 51.28) | 7636.06 | 50.27 (43.99) | 5.65 (5.42) |
| Haemorrhage* | 106 | 8.53 (7.04, 10.34) | 8.4 (8.21, 8.59) | 690.28 | 8.38 (7.13) | 3.07 (2.78) |
| Haemoglobin decreased* | 97 | 7.47 (6.11, 9.14) | 7.37 (7.17, 7.57) | 533.9 | 7.35 (6.22) | 2.88 (2.58) |
| Cerebral haemorrhage* | 73 | 14.73 (11.69, 18.56) | 14.57 (14.34, 14.79) | 918.53 | 14.5 (11.95) | 3.86 (3.52) |
| Pain in extremity* | 59 | 2.41 (1.87, 3.12) | 2.4 (2.15, 2.66) | 48.39 | 2.4 (1.94) | 1.26 (0.89) |
| Muscle haemorrhage* | 57 | 80.82 (62.05, 105.27) | 80.08 (79.82, 80.34) | 4333.94 | 77.99 (62.51) | 6.29 (5.9) |
| Thrombocytopenia* | 56 | 3.94 (3.03, 5.13) | 3.92 (3.66, 4.18) | 121.81 | 3.91 (3.14) | 1.97 (1.58) |
| Melaena* | 52 | 15.11 (11.49, 19.86) | 14.99 (14.71, 15.26) | 675.66 | 14.91 (11.86) | 3.9 (3.5) |
| Oedema peripheral* | 51 | 3.87 (2.94, 5.1) | 3.85 (3.58, 4.12) | 107.66 | 3.85 (3.05) | 1.94 (1.54) |
| Gastrointestinal haemorrhage* | 49 | 4.06 (3.06, 5.37) | 4.03(3.75,4.31) | 111.75 | 4.03 (3.18) | 2.01 (1.6) |
| Shock haemorrhagic* | 47 | 40.12 (30.06, 53.56) | 39.82 (39.54, 40.11) | 1755.49 | 39.31 (30.86) | 5.3 (4.88) |
| Dyspnoea | 47 | 0.8 (0.6, 1.07) | 0.81 (0.52, 1.09) | 2.21 | 0.81 (0.63) | -0.31 (-0.73) |
| Pyrexia | 45 | 1.09 (0.82, 1.47) | 1.09 (0.8, 1.38) | 0.36 | 1.09 (0.86) | 0.13 (-0.3) |
| Haematuria* | 43 | 7.12 (5.27, 9.62) | 7.08 (6.78, 7.38) | 224.13 | 7.06 (5.49) | 2.82 (2.38) |
| Abdominal pain* | 43 | 2.01 (1.49, 2.72) | 2.01 (1.71, 2.3) | 21.75 | 2.01 (1.56) | 1 (0.57) |
| Post procedural haemorrhage* | 43 | 34.02 (25.16, 46) | 33.79 (33.49, 34.09) | 1353.04 | 33.42 (25.96) | 5.06 (4.62) |
| Headache | 42 | 0.88 (0.65, 1.19) | 0.88 (0.58, 1.18) | 0.67 | 0.88 (0.68) | -0.18 (-0.62) |
| Fall | 41 | 1.28 (0.94, 1.74) | 1.28 (0.97, 1.58) | 2.47 | 1.28 (0.99) | 0.35 (-0.09) |
| Hypotension* | 40 | 1.59 (1.16, 2.16) | 1.58 (1.27, 1.89) | 8.58 | 1.58 (1.22) | 0.66 (0.21) |
| Haematemesis* | 38 | 11.03 (8.01, 15.18) | 10.97 (10.65, 11.29) | 343.21 | 10.93 (8.37) | 3.45 (2.99) |
| Vomiting | 37 | 0.94 (0.68, 1.3) | 0.94 (0.62, 1.26) | 0.14 | 0.94 (0.72) | -0.09 (-0.56) |
| Malaise | 34 | 0.88 (0.63, 1.24) | 0.89 (0.55, 1.22) | 0.51 | 0.89 (0.67) | -0.18 (-0.67) |
| Subdural haematoma* | 33 | 13.72 (9.74, 19.33) | 13.65 (13.31, 13.99) | 385.26 | 13.59 (10.2) | 3.76 (3.27) |
| Injection site pain* | 33 | 1.53 (1.09, 2.15) | 1.53 (1.19, 1.87) | 6.03 | 1.53 (1.15) | 0.61 (0.11) |
| Pain | 31 | 0.56 (0.39, 0.79) | 0.56 (0.21, 0.91) | 10.78 | 0.56 (0.42) | -0.84 (-1.35) |
| Asthenia | 31 | 0.75 (0.53, 1.07) | 0.75 (0.4, 1.1) | 2.54 | 0.75 (0.56) | -0.41 (-0.92) |
| Cerebrovascular accident | 30 | 1.41 (0.98, 2.01) | 1.4 (1.05, 1.76) | 3.49 | 1.4 (1.04) | 0.49 (-0.03) |
| Coma* | 29 | 4.9 (3.4, 7.06) | 4.88 (4.52, 5.25) | 89.5 | 4.88 (3.59) | 2.29 (1.76) |
| Platelet count decreased* | 29 | 2.13 (1.48, 3.07) | 2.12 (1.76, 2.49) | 17.27 | 2.12 (1.56) | 1.09 (0.56) |
| Nausea | 29 | 0.48 (0.34, 0.7) | 0.49 (0.12, 0.85) | 15.79 | 0.49 (0.36) | -1.04 (-1.56) |
| Renal failure | 28 | 1.42 (0.98, 2.06) | 1.42 (1.05, 1.79) | 3.47 | 1.42 (1.04) | 0.5 (-0.03) |
| Retroperitoneal haematoma* | 28 | 90.96 (62.4, 132.6) | 90.55 (90.17, 90.92) | 2405.86 | 87.88 (64.11) | 6.46 (5.91) |
| General physical health deterioration* | 28 | 2.15 (1.48, 3.11) | 2.14 (1.77, 2.51) | 17.04 | 2.14 (1.57) | 1.1 (0.56) |
| Dizziness | 27 | 0.58 (0.4, 0.85) | 0.58 (0.21, 0.96) | 8.02 | 0.58 (0.43) | -0.77 (-1.32) |
| Heparin-induced thrombocytopenia* | 27 | 36.83 (25.18, 53.88) | 36.67 (36.29, 37.05) | 925.5 | 36.23 (26.36) | 5.18 (4.63) |
| Haematocrit decreased* | 27 | 9.79 (6.7, 14.29) | 9.75 (9.37, 10.12) | 211.33 | 9.72 (7.08) | 3.28 (2.73) |
| Condition aggravated | 25 | 0.89 (0.6, 1.32) | 0.89 (0.5, 1.28) | 0.32 | 0.89 (0.64) | -0.16 (-0.73) |
| Chest pain | 24 | 1.21 (0.81, 1.81) | 1.21 (0.81, 1.61) | 0.87 | 1.21 (0.86) | 0.27 (-0.3) |
| Epistaxis* | 24 | 2.65 (1.77, 3.96) | 2.64 (2.24, 3.04) | 24.51 | 2.64 (1.89) | 1.4 (0.82) |
| Confusional state | 23 | 1.19 (0.79, 1.79) | 1.19 (0.78, 1.59) | 0.67 | 1.19 (0.84) | 0.25 (-0.34) |
| Pallor* | 23 | 6.7 (4.45, 10.09) | 6.68 (6.27, 7.09) | 110.84 | 6.66 (4.73) | 2.74 (2.15) |
| Arthralgia | 21 | 0.63 (0.41, 0.97) | 0.63 (0.2, 1.06) | 4.54 | 0.63 (0.44) | -0.66 (-1.28) |
| Haemorrhage intracranial* | 21 | 9.95 (6.48, 15.28) | 9.92 (9.49, 10.35) | 167.92 | 9.89 (6.91) | 3.31 (2.69) |
| Contusion* | 21 | 2.84 (1.85, 4.36) | 2.84 (2.41, 3.26) | 24.97 | 2.83 (1.98) | 1.5 (0.89) |
| Cardiac arrest* | 20 | 1.66 (1.07, 2.58) | 1.66 (1.22, 2.1) | 5.28 | 1.66 (1.15) | 0.73 (0.1) |
| Fatigue | 19 | 0.26 (0.16, 0.4) | 0.26 (-0.19, 0.71) | 40.71 | 0.26 (0.18) | -1.95 (-2.59) |
| Tachycardia* | 19 | 1.98 (1.26, 3.11) | 1.98 (1.53, 2.43) | 9.23 | 1.98 (1.36) | 0.99 (0.34) |
| Gait disturbance | 19 | 1.02 (0.65, 1.61) | 1.02 (0.57, 1.47) | 0.01 | 1.02 (0.7) | 0.03 (-0.61) |
| Rectal haemorrhage* | 19 | 3.45 (2.2, 5.42) | 3.44 (2.99, 3.89) | 32.93 | 3.44 (2.36) | 1.78 (1.14) |

Abbreviation: Asterisks (*) indicate positive signals in algorithm; ROR, reporting odds ratio; PRR, proportional reporting ratio; EBGM, empirical Bayesian geometric mean; EBGM05, the lower limit of the 95% CI of EBGM; IC, information component; IC025, the lower limit of the 95% CI of the IC; χ2, chi-squared; CI, confidence interval; PT, preferred term; AEs, adverse events.

Supplementary Table 7:

Top 50 most frequent AEs for fondaparinux sodium at the PT level in females from FAERS data

| PT | Case numbers | ROR(95%CI) | PRR(95%CI) | χ2 | EBGM(EBGM05) | IC(IC025) |
| --- | --- | --- | --- | --- | --- | --- |
| Haematoma* | 313 | 88.69 (79.12, 99.41) | 85.63 (85.52, 85.74) | 25536.43 | 83.52 (75.91) | 6.38 (6.22) |
| Anaemia* | 287 | 11.23 (9.98, 12.63) | 10.9 (10.79, 11.02) | 2580.84 | 10.87 (9.85) | 3.44 (3.27) |
| Haemoglobin decreased* | 170 | 12.52 (10.76, 14.58) | 12.3 (12.15, 12.45) | 1761.66 | 12.26 (10.8) | 3.62 (3.39) |
| Haemorrhage* | 130 | 10.87 (9.14, 12.93) | 10.73 (10.55, 10.9) | 1144.34 | 10.69 (9.25) | 3.42 (3.16) |
| Muscle haemorrhage* | 112 | 397.75 (326.66, 484.3) | 392.8 (392.61, 393) | 39164.21 | 351.56 (298.16) | 8.46 (8.17) |
| Abdominal pain* | 87 | 2.31 (1.87, 2.85) | 2.3 (2.09, 2.51) | 63.94 | 2.3 (1.92) | 1.2 (0.89) |
| Shock haemorrhagic* | 83 | 96.74 (77.7, 120.45) | 95.86 (95.64, 96.07) | 7574.31 | 93.21 (77.59) | 6.54 (6.22) |
| Melaena* | 83 | 34.12 (27.46, 42.4) | 33.82 (33.6, 34.03) | 2617.41 | 33.49 (27.92) | 5.07 (4.75) |
| Dyspnoea | 81 | 0.92 (0.74, 1.15) | 0.92 (0.71, 1.14) | 0.54 | 0.92 (0.77) | -0.12 (-0.44) |
| Cerebral haemorrhage* | 77 | 19.32 (15.43, 24.19) | 19.16 (18.94, 19.38) | 1318.37 | 19.06 (15.79) | 4.25 (3.92) |
| Pain in extremity | 69 | 1.27 (1, 1.61) | 1.27 (1.03, 1.5) | 3.97 | 1.27 (1.04) | 0.34 (0) |
| Vomiting | 68 | 0.87 (0.69, 1.1) | 0.87 (0.63, 1.11) | 1.32 | 0.87 (0.71) | -0.2 (-0.55) |
| Post procedural haemorrhage* | 67 | 38.56 (30.28, 49.1) | 38.28 (38.04, 38.52) | 2405.33 | 37.86 (30.92) | 5.24 (4.89) |
| Hypotension* | 66 | 2.48 (1.94, 3.16) | 2.47 (2.23, 2.71) | 57.68 | 2.47 (2.01) | 1.3 (0.95) |
| Thrombocytopenia* | 66 | 5.5 (4.31, 7) | 5.46 (5.22, 5.7) | 240.52 | 5.45 (4.45) | 2.45 (2.09) |
| Oedema peripheral* | 64 | 3.24 (2.53, 4.14) | 3.22 (2.98, 3.46) | 98.12 | 3.22 (2.62) | 1.69 (1.33) |
| Nausea | 60 | 0.43 (0.33, 0.55) | 0.43 (0.18, 0.68) | 45.69 | 0.43 (0.35) | -1.21 (-1.58) |
| Gastrointestinal haemorrhage* | 60 | 6.19 (4.8, 7.97) | 6.15 (5.9, 6.4) | 258.59 | 6.14 (4.96) | 2.62 (2.25) |
| Haematemesis* | 57 | 17.65 (13.6, 22.92) | 17.55 (17.29, 17.81) | 885.09 | 17.46 (14.03) | 4.13 (3.74) |
| Renal failure* | 57 | 3.73 (2.87, 4.84) | 3.71 (3.45, 3.97) | 113.08 | 3.71 (2.98) | 1.89 (1.51) |
| Pyrexia | 55 | 1.12 (0.86, 1.46) | 1.12 (0.85, 1.38) | 0.66 | 1.12 (0.89) | 0.16 (-0.23) |
| Heparin-induced thrombocytopenia* | 54 | 108.54 (82.71, 142.44) | 107.9 (107.63, 108.17) | 5540.25 | 104.55 (83.28) | 6.71 (6.31) |
| Malaise | 54 | 0.7 (0.54, 0.92) | 0.7 (0.44, 0.97) | 6.78 | 0.7 (0.56) | -0.51 (-0.9) |
| Contusion* | 53 | 3.11 (2.38, 4.08) | 3.1 (2.83, 3.37) | 75.48 | 3.1 (2.47) | 1.63 (1.24) |
| Subdural haematoma* | 50 | 34.96 (26.44, 46.23) | 34.77 (34.49, 35.05) | 1623.41 | 34.42 (27.25) | 5.11 (4.7) |
| Fall | 49 | 0.88 (0.67, 1.17) | 0.89 (0.61, 1.16) | 0.73 | 0.89 (0.7) | -0.18 (-0.58) |
| Pain | 49 | 0.46 (0.35, 0.61) | 0.46 (0.18, 0.74) | 30.91 | 0.46 (0.37) | -1.11 (-1.52) |
| Injection site pain | 49 | 0.95 (0.72, 1.26) | 0.95 (0.67, 1.23) | 0.13 | 0.95 (0.75) | -0.07 (-0.48) |
| Headache | 48 | 0.42 (0.32, 0.56) | 0.43 (0.14, 0.71) | 37.44 | 0.43 (0.34) | -1.23 (-1.64) |
| Injection site haematoma* | 48 | 16.02 (12.05, 21.28) | 15.94 (15.65, 16.22) | 669.03 | 15.87 (12.51) | 3.99 (3.57) |
| Pallor* | 47 | 12.2 (9.16, 16.26) | 12.15 (11.86, 12.43) | 479.19 | 12.11 (9.52) | 3.6 (3.18) |
| Red blood cell count decreased* | 47 | 11.42 (8.57, 15.22) | 11.36 (11.08, 11.65) | 442.9 | 11.33 (8.91) | 3.5 (3.08) |
| Platelet count decreased* | 44 | 3.29 (2.45, 4.43) | 3.28 (2.99, 3.58) | 69.83 | 3.28 (2.56) | 1.71 (1.28) |
| Confusional state* | 42 | 1.8 (1.33, 2.44) | 1.8 (1.5, 2.1) | 14.94 | 1.8 (1.4) | 0.85 (0.41) |
| Epistaxis* | 39 | 3.79 (2.76, 5.19) | 3.77 (3.46, 4.09) | 79.51 | 3.77 (2.9) | 1.91 (1.46) |
| Coma* | 37 | 5.64 (4.08, 7.79) | 5.62 (5.3, 5.94) | 140.46 | 5.61 (4.28) | 2.49 (2.02) |
| Asthenia | 36 | 0.63 (0.45, 0.87) | 0.63 (0.3, 0.95) | 7.96 | 0.63 (0.48) | -0.67 (-1.15) |
| Abdominal wall haematoma* | 35 | 119.83 (85.48, 167.98) | 119.37 (119.03, 119.7) | 3966.42 | 115.28 (86.9) | 6.85 (6.36) |
| Intra-abdominal haematoma* | 35 | 211 (149.85, 297.09) | 210.18 (209.84, 210.52) | 6855.01 | 197.79 (148.54) | 7.63 (7.13) |
| General physical health deterioration* | 35 | 2.38 (1.71, 3.32) | 2.37 (2.04, 2.7) | 27.84 | 2.37 (1.8) | 1.25 (0.76) |
| Retroperitoneal haematoma* | 35 | 136.09 (97, 190.94) | 135.57 (135.23, 135.9) | 4492.82 | 130.32 (98.17) | 7.03 (6.53) |
| Haematuria* | 34 | 10.82 (7.72, 15.16) | 10.78 (10.44, 11.12) | 300.8 | 10.75 (8.11) | 3.43 (2.94) |
| Post procedural haematoma* | 34 | 152.13 (107.82, 214.66) | 151.56 (151.22, 151.91) | 4864.64 | 145.02 (108.72) | 7.18 (6.68) |
| Urticaria | 34 | 1.22 (0.87, 1.72) | 1.22 (0.89, 1.56) | 1.4 | 1.22 (0.92) | 0.29 (-0.2) |
| Chest pain | 33 | 1.12 (0.79, 1.57) | 1.12 (0.78, 1.46) | 0.4 | 1.12 (0.84) | 0.16 (-0.34) |
| Shock* | 33 | 12.06 (8.56, 16.98) | 12.02 (11.68, 12.36) | 332.2 | 11.98 (8.99) | 3.58 (3.09) |
| Cerebrovascular accident | 32 | 1.38 (0.98, 1.95) | 1.38 (1.03, 1.72) | 3.33 | 1.38 (1.03) | 0.46 (-0.04) |
| Rectal haemorrhage* | 32 | 5.5 (3.88, 7.78) | 5.48 (5.13, 5.83) | 117.08 | 5.47 (4.09) | 2.45 (1.95) |
| Pruritus | 32 | 0.55 (0.39, 0.78) | 0.55 (0.21, 0.9) | 11.61 | 0.55 (0.41) | -0.85 (-1.36) |
| Haematocrit decreased* | 31 | 11.37 (7.99, 16.19) | 11.33 (10.98, 11.68) | 291.16 | 11.3 (8.41) | 3.5 (2.99) |

Abbreviation: Asterisks (*) indicate positive signals in algorithm; ROR, reporting odds ratio; PRR, proportional reporting ratio; EBGM, empirical Bayesian geometric mean; EBGM05, the lower limit of the 95% CI of EBGM; IC, information component; IC025, the lower limit of the 95% CI of the IC; χ2, chi-squared; CI, confidence interval; PT,preferred term; AEs, adverse events.

Supplementary Table 8:

AEs at the PT level for fondaparinux sodium in patients aged under 18 from FAERS data

| PT | Case numbers | ROR(95%CI) | PRR(95%CI) | χ2 | EBGM(EBGM05) | IC(IC025) |
| --- | --- | --- | --- | --- | --- | --- |
| Hepatitis* | 3 | 71.07 (22.39, 225.58) | 68.44 (67.33, 69.55) | 198.9 | 68.25 (25.96) | 6.09 (4.61) |
| Vomiting* | 3 | 2.93 (0.92, 9.27) | 2.85 (1.74, 3.96) | 3.66 | 2.85 (1.09) | 1.51 (0.03) |
| Gamma-glutamyltransferase increased* | 2 | 55.67 (13.66, 226.92) | 54.3 (52.93, 55.67) | 104.45 | 54.18 (16.72) | 5.76 (4.06) |
| Haemorrhage* | 2 | 12.95 (3.18, 52.73) | 12.65 (11.28, 14.02) | 21.49 | 12.65 (3.91) | 3.66 (1.96) |
| Alanine aminotransferase increased* | 2 | 12.94 (3.18, 52.7) | 12.65 (11.28, 14.01) | 21.48 | 12.64 (3.9) | 3.66 (1.96) |
| Hepatotoxicity* | 2 | 44.34 (10.88, 180.67) | 43.25 (41.88, 44.62) | 82.45 | 43.18 (13.33) | 5.43 (3.73) |
| Nausea* | 2 | 3.41 (0.84, 13.9) | 3.35 (1.98, 4.72) | 3.33 | 3.35 (1.04) | 1.75 (0.05) |
| Accidental exposure to product* | 1 | 7.75 (1.08, 55.74) | 7.67(5.72,9.62) | 5.81 | 7.67 (1.47) | 2.94 (0.87) |
| Anaphylactic reaction* | 1 | 7.22 (1, 51.87) | 7.14 (5.19, 9.09) | 5.29 | 7.14 (1.37) | 2.84 (0.77) |
| Spontaneous haematoma* | 1 | 3000.39 (370.9, 24271.39) | 2962.89 (2960.83, 2964.96) | 2631.92 | 2633.79 (458.03) | 11.36 (9.11) |
| Skin necrosis* | 1 | 125.66 (17.39, 907.8) | 124.1 (122.15, 126.05) | 121.48 | 123.46 (23.6) | 6.95 (4.87) |
| Eosinophilia* | 1 | 32.78 (4.55, 235.92) | 32.38 (30.43, 34.33) | 30.38 | 32.34 (6.2) | 5.02 (2.95) |
| Epistaxis* | 1 | 7.24 (1.01, 52.05) | 7.16 (5.21, 9.11) | 5.31 | 7.16 (1.37) | 2.84 (0.77) |
| Alopecia* | 1 | 11.23 (1.56, 80.75) | 11.1 (9.15, 13.05) | 9.2 | 11.1 (2.13) | 3.47 (1.41) |
| Diarrhoea | 1 | 1.78 (0.25, 12.78) | 1.77 (-0.18, 3.72) | 0.34 | 1.77 (0.34) | 0.82 (-1.24) |
| Exposure via breast milk* | 1 | 34.67 (4.82, 249.58) | 34.25 (32.3, 36.2) | 32.25 | 34.21 (6.56) | 5.1 (3.03) |
| Haematochezia* | 1 | 9.39 (1.31, 67.49) | 9.28 (7.33, 11.23) | 7.4 | 9.28 (1.78) | 3.21 (1.15) |
| Foetal exposure during pregnancy | 1 | 3.82 (0.53, 27.47) | 3.79 (1.84, 5.73) | 2.06 | 3.79 (0.73) | 1.92 (-0.15) |
| Circulatory collapse* | 1 | 30.76 (4.27, 221.37) | 30.39 (28.44, 32.34) | 28.4 | 30.35 (5.82) | 4.92 (2.86) |
| Hypotension | 1 | 3.59 (0.5, 25.83) | 3.56 (1.61, 5.51) | 1.85 | 3.56 (0.68) | 1.83 (-0.23) |
| Intra-abdominal haemorrhage* | 1 | 347.86 (47.72, 2535.71) | 343.52 (341.56, 345.49) | 336.66 | 338.63 (64.25) | 8.4 (6.31) |
| Angioedema* | 1 | 12.28 (1.71, 88.29) | 12.14 (10.19, 14.09) | 10.22 | 12.13 (2.33) | 3.6 (1.53) |
| Fatigue | 1 | 2.31 (0.32, 16.59) | 2.29 (0.34, 4.24) | 0.73 | 2.29 (0.44) | 1.2 (-0.87) |
| Hepatocellular injury* | 1 | 29.99 (4.17, 215.83) | 29.63 (27.68, 31.58) | 27.64 | 29.59 (5.68) | 4.89 (2.82) |
| Transaminases increased* | 1 | 16.08 (2.24, 115.62) | 15.89 (13.94, 17.84) | 13.95 | 15.88 (3.05) | 3.99 (1.92) |
| Arthralgia* | 1 | 4.32 (0.6, 31.08) | 4.28 (2.33, 6.23) | 2.52 | 4.28 (0.82) | 2.1 (0.03) |
| Erythema | 1 | 1.3 (0.18, 9.34) | 1.3 (-0.65, 3.24) | 0.07 | 1.3 (0.25) | 0.37 (-1.69) |
| Haemarthrosis* | 1 | 17.99 (2.5, 129.43) | 17.78 (15.83, 19.73) | 15.84 | 17.77 (3.41) | 4.15 (2.08) |
| Joint effusion* | 1 | 83.33 (11.55, 600.99) | 82.3 (80.35, 84.25) | 80.05 | 82.02 (15.7) | 6.36 (4.29) |
| Joint swelling* | 1 | 17.34 (2.41, 124.75) | 17.14 (15.19, 19.09) | 15.2 | 17.13 (3.29) | 4.1 (2.03) |
| Subcutaneous haematoma* | 1 | 727.36 (98.27, 5383.41) | 718.28 (716.3, 720.25) | 695.22 | 697.18 (130.61) | 9.45 (7.32) |
| Injection site irritation* | 1 | 135.6 (18.76, 980.01) | 133.92 (131.96, 135.87) | 131.19 | 133.17 (25.45) | 7.06 (4.98) |
| Volume blood decreased* | 1 | 2667.01 (333.94, 21300.16) | 2633.68 (2631.63, 2635.74) | 2368.53 | 2370.42 (416.67) | 11.21 (8.97) |
| Haematuria* | 1 | 20.72 (2.88, 149.02) | 20.47 (18.52, 22.42) | 18.51 | 20.45 (3.92) | 4.35 (2.29) |
| Pleural effusion* | 1 | 13.56 (1.88, 97.49) | 13.4 (11.45, 15.35) | 11.48 | 13.39 (2.57) | 3.74 (1.68) |
| Abdominal pain | 1 | 2.39 (0.33, 17.19) | 2.37 (0.43, 4.32) | 0.8 | 2.37 (0.46) | 1.25 (-0.82) |
| Anxiety | 1 | 3.08 (0.43, 22.17) | 3.06 (1.11, 5.01) | 1.39 | 3.06 (0.59) | 1.61 (-0.45) |
| Blood creatinine increased* | 1 | 14.41 (2, 103.65) | 14.24 (12.3, 16.19) | 12.32 | 14.24 (2.73) | 3.83 (1.77) |
| Hypertension* | 1 | 5.11 (0.71, 36.75) | 5.06 (3.11, 7.01) | 3.27 | 5.06 (0.97) | 2.34 (0.27) |
| Klebsiella sepsis* | 1 | 190.49 (26.3, 1379.77) | 188.12 (186.16, 190.08) | 184.67 | 186.65 (35.6) | 7.54 (5.46) |
| Platelet count decreased* | 1 | 8.84 (1.23, 63.55) | 8.74 (6.79, 10.69) | 6.86 | 8.74 (1.68) | 3.13 (1.06) |
| Pneumothorax* | 1 | 28.16 (3.91, 202.64) | 27.82 (25.87, 29.77) | 25.84 | 27.79 (5.33) | 4.8 (2.73) |
| Pulmonary haemorrhage* | 1 | 28.7 (3.99, 206.52) | 28.35 (26.4, 30.3) | 26.37 | 28.32 (5.43) | 4.82 (2.76) |
| Pulmonary hypertension* | 1 | 25.07 (3.48, 180.37) | 24.77 (22.82, 26.72) | 22.8 | 24.74 (4.75) | 4.63 (2.56) |
| Respiratory distress* | 1 | 7.83 (1.09, 56.31) | 7.75 (5.8, 9.69) | 5.88 | 7.74 (1.49) | 2.95 (0.89) |
| Respiratory failure* | 1 | 5.89 (0.82, 42.33) | 5.83 (3.88, 7.77) | 4.01 | 5.83 (1.12) | 2.54 (0.48) |
| Blood alkaline phosphatase increased* | 1 | 23.73 (3.3, 170.72) | 23.45 (21.5, 25.39) | 21.48 | 23.42 (4.49) | 4.55 (2.48) |
| Blood bilirubin increased* | 1 | 18.8 (2.61, 135.22) | 18.58 (16.63, 20.52) | 16.63 | 18.56 (3.56) | 4.21 (2.15) |
| Gastrointestinal haemorrhage* | 1 | 19.84 (2.76, 142.73) | 19.61 (17.66, 21.55) | 17.65 | 19.59 (3.76) | 4.29 (2.23) |
| Aspartate aminotransferase increased* | 1 | 7.13 (0.99, 51.29) | 7.06 (5.11, 9) | 5.21 | 7.05 (1.35) | 2.82 (0.75) |

Abbreviation: Asterisks (*) indicate positive signals in algorithm; ROR, reporting odds ratio; PRR, proportional reporting ratio; EBGM, empirical Bayesian geometric mean; EBGM05, the lower limit of the 95% CI of EBGM; IC, information component; IC025, the lower limit of the 95% CI of the IC; χ2, chi-squared; CI, confidence interval; PT, preferred term.

Supplementary Table 9:

Top 50 most frequent AEs for fondaparinux sodium at the PT level in patients aged 18-64 from FAERS data

| PT | Case numbers | ROR(95%CI) | PRR(95%CI) | χ2 | EBGM(EBGM05) | IC(IC025) |
| --- | --- | --- | --- | --- | --- | --- |
| Haematoma* | 86 | 58.14 (46.9, 72.06) | 57.09 (56.88, 57.3) | 4678.94 | 56.36 (47.09) | 5.82 (5.5) |
| Anaemia* | 77 | 5.89 (4.71, 7.38) | 5.81 (5.59, 6.04) | 307.4 | 5.81 (4.81) | 2.54 (2.21) |
| Haemoglobin decreased* | 66 | 9.06 (7.11, 11.56) | 8.95 (8.71, 9.19) | 465.94 | 8.94 (7.29) | 3.16 (2.8) |
| Haemorrhage* | 64 | 9.98 (7.79, 12.77) | 9.86 (9.61, 10.1) | 508.76 | 9.83 (8) | 3.3 (2.94) |
| Pain in extremity* | 51 | 1.98 (1.5, 2.61) | 1.97 (1.7, 2.25) | 24.58 | 1.97 (1.57) | 0.98 (0.58) |
| Headache | 49 | 0.81 (0.61, 1.07) | 0.81 (0.53, 1.09) | 2.24 | 0.81 (0.64) | -0.31 (-0.72) |
| Pyrexia* | 49 | 1.54 (1.16, 2.04) | 1.54 (1.26, 1.81) | 9.2 | 1.53 (1.21) | 0.62 (0.21) |
| Dyspnoea | 45 | 1.03 (0.77, 1.38) | 1.03 (0.74, 1.32) | 0.03 | 1.03 (0.8) | 0.04 (-0.39) |
| Injection site pain* | 44 | 1.71 (1.27, 2.3) | 1.7 (1.41, 1.99) | 12.72 | 1.7 (1.33) | 0.76 (0.33) |
| Muscle haemorrhage* | 43 | 181.92 (133.91, 247.16) | 180.27 (179.97, 180.58) | 7358.52 | 173.07 (133.93) | 7.44 (6.99) |
| Vomiting | 40 | 0.98 (0.72, 1.33) | 0.98 (0.67, 1.28) | 0.02 | 0.98 (0.75) | -0.03 (-0.49) |
| Cerebral haemorrhage* | 40 | 21.32 (15.6, 29.12) | 21.14 (20.84, 21.45) | 764.23 | 21.05 (16.21) | 4.4 (3.94) |
| Thrombocytopenia* | 40 | 4.93 (3.61, 6.73) | 4.89 (4.59, 5.2) | 124 | 4.89 (3.77) | 2.29 (1.84) |
| Nausea | 36 | 0.5 (0.36, 0.7) | 0.51 (0.18, 0.83) | 17.71 | 0.51 (0.38) | -0.98 (-1.46) |
| Abdominal pain* | 33 | 1.47 (1.04, 2.06) | 1.46 (1.12, 1.8) | 4.84 | 1.46 (1.1) | 0.55 (0.05) |
| Post procedural haemorrhage* | 32 | 32.5 (22.93, 46.08) | 32.29 (31.94, 32.64) | 963.27 | 32.06 (23.94) | 5 (4.5) |
| Shock haemorrhagic* | 31 | 62.78 (43.99, 89.6) | 62.37 (62.02, 62.73) | 1845.54 | 61.5 (45.67) | 5.94 (5.43) |
| Heparin-induced thrombocytopenia* | 31 | 73.89 (51.75, 105.5) | 73.41 (73.05, 73.76) | 2177.18 | 72.2 (53.59) | 6.17 (5.66) |
| Contusion* | 31 | 4.43 (3.11, 6.31) | 4.41 (4.05, 4.76) | 81.66 | 4.4 (3.28) | 2.14 (1.63) |
| Chest pain* | 30 | 1.65 (1.15, 2.36) | 1.64 (1.29, 2) | 7.6 | 1.64 (1.22) | 0.72 (0.2) |
| Oedema peripheral* | 29 | 3 (2.08, 4.32) | 2.99 (2.62, 3.35) | 38.4 | 2.99 (2.2) | 1.58 (1.05) |
| Melaena* | 27 | 23.12 (15.82, 33.78) | 22.99 (22.62, 23.37) | 565.09 | 22.88 (16.65) | 4.52 (3.97) |
| Malaise | 27 | 0.8 (0.55, 1.17) | 0.8 (0.43, 1.18) | 1.29 | 0.8 (0.59) | -0.31 (-0.86) |
| Hypotension* | 27 | 1.77 (1.21, 2.58) | 1.76 (1.39, 2.14) | 8.91 | 1.76 (1.28) | 0.82 (0.27) |
| Asthenia | 26 | 0.93 (0.63, 1.36) | 0.93 (0.55, 1.31) | 0.14 | 0.93 (0.67) | -0.11 (-0.66) |
| Injection site haematoma* | 26 | 19.51 (13.26, 28.71) | 19.41 (19.02, 19.79) | 452 | 19.32 (13.99) | 4.27 (3.71) |
| Haematemesis* | 25 | 12.87 (8.68, 19.07) | 12.8 (12.41, 13.2) | 271.35 | 12.77 (9.19) | 3.67 (3.11) |
| Dizziness | 24 | 0.59 (0.39, 0.88) | 0.59 (0.19, 0.99) | 6.85 | 0.59 (0.42) | -0.76 (-1.34) |
| Urticaria* | 24 | 1.58 (1.06, 2.37) | 1.58 (1.18, 1.98) | 5.15 | 1.58 (1.13) | 0.66 (0.08) |
| Pain | 23 | 0.46 (0.31, 0.7) | 0.47 (0.06, 0.87) | 14.15 | 0.47 (0.33) | -1.1 (-1.69) |
| Rash | 22 | 0.68 (0.45, 1.04) | 0.69 (0.27, 1.1) | 3.19 | 0.69 (0.48) | -0.54 (-1.15) |
| Coma* | 22 | 4.36 (2.87, 6.63) | 4.35 (3.93, 4.76) | 56.7 | 4.34 (3.06) | 2.12 (1.52) |
| Platelet count decreased* | 22 | 3.08 (2.03, 4.69) | 3.07 (2.65, 3.49) | 30.76 | 3.07 (2.16) | 1.62 (1.02) |
| Pruritus | 21 | 0.71 (0.46, 1.09) | 0.71 (0.29, 1.14) | 2.44 | 0.71 (0.5) | -0.49 (-1.1) |
| Epistaxis* | 20 | 4.15 (2.67, 6.44) | 4.14 (3.7, 4.57) | 47.57 | 4.13 (2.86) | 2.05 (1.42) |
| Exposure during pregnancy* | 19 | 3.28 (2.09, 5.15) | 3.27 (2.82, 3.72) | 29.96 | 3.27 (2.24) | 1.71 (1.06) |
| Confusional state* | 19 | 1.63 (1.04, 2.56) | 1.63 (1.18, 2.08) | 4.66 | 1.63 (1.12) | 0.71 (0.06) |
| Diarrhoea | 19 | 0.4 (0.26, 0.63) | 0.4 (-0.04, 0.85) | 16.82 | 0.4 (0.28) | -1.31 (-1.95) |
| Fall | 18 | 0.94 (0.59, 1.49) | 0.94 (0.48, 1.4) | 0.07 | 0.94 (0.64) | -0.09 (-0.75) |
| Subdural haematoma* | 18 | 30.78 (19.34, 48.97) | 30.66 (30.2, 31.12) | 512.92 | 30.45 (20.65) | 4.93 (4.26) |
| Injection site haemorrhage* | 18 | 2.52 (1.59, 4.01) | 2.52 (2.06, 2.98) | 16.47 | 2.52 (1.71) | 1.33 (0.67) |
| Haematuria* | 18 | 8.37 (5.26, 13.3) | 8.34 (7.88, 8.8) | 116.07 | 8.32 (5.65) | 3.06 (2.39) |
| Haematocrit decreased* | 18 | 10.25 (6.45, 16.3) | 10.22 (9.76, 10.68) | 149.39 | 10.2 (6.92) | 3.35 (2.69) |
| Gastrointestinal haemorrhage* | 17 | 4.01 (2.49, 6.45) | 4 (3.52, 4.47) | 38.18 | 3.99 (2.68) | 2 (1.32) |
| Condition aggravated | 16 | 0.65 (0.4, 1.07) | 0.66 (0.17, 1.14) | 2.91 | 0.66 (0.43) | -0.61 (-1.31) |
| Cerebrovascular accident | 16 | 1.61 (0.99, 2.64) | 1.61 (1.12, 2.1) | 3.73 | 1.61 (1.07) | 0.69 (-0.01) |
| Erythema | 16 | 0.91 (0.56, 1.49) | 0.92 (0.43, 1.4) | 0.13 | 0.92 (0.61) | -0.13 (-0.83) |
| Arthralgia | 15 | 0.41 (0.25, 0.69) | 0.42 (-0.09, 0.92) | 12.46 | 0.42 (0.27) | -1.27 (-1.99) |
| Fatigue | 15 | 0.23 (0.14, 0.39) | 0.23 (-0.27, 0.74) | 38.03 | 0.23 (0.15) | -2.09 (-2.82) |
| Tachycardia | 15 | 1.65 (0.99, 2.74) | 1.65 (1.14, 2.15) | 3.82 | 1.65 (1.08) | 0.72 (0) |

Abbreviation: Asterisks (*) indicate positive signals in algorithm; ROR, reporting odds ratio; PRR, proportional reporting ratio; EBGM, empirical Bayesian geometric mean; EBGM05, the lower limit of the 95% CI of EBGM; IC, information component; IC025, the lower limit of the 95% CI of the IC; χ2, chi-squared; CI, confidence interval; PT, preferred term.

Supplementary Table 10:

Top 50 most frequent AEs for fondaparinux sodium at the PT level in patients aged over 65 from FAERS data

| PT | Case numbers | ROR(95%CI) | PRR(95%CI) | χ2 | EBGM(EBGM05) | IC(IC025) |
| --- | --- | --- | --- | --- | --- | --- |
| Anaemia* | 316 | 8.01 (7.16, 8.97) | 7.72 (7.61, 7.83) | 1849.42 | 7.69 (6.99) | 2.94 (2.78) |
| Haematoma* | 307 | 46.74 (41.63, 52.47) | 44.91 (44.8, 45.02) | 12811.4 | 43.64 (39.61) | 5.45 (5.28) |
| Haemoglobin decreased* | 152 | 7.26 (6.18, 8.52) | 7.13 (6.97, 7.29) | 799.84 | 7.1 (6.21) | 2.83 (2.59) |
| Haemorrhage* | 113 | 7.73 (6.42, 9.31) | 7.63 (7.45, 7.81) | 648.94 | 7.6 (6.5) | 2.93 (2.65) |
| Muscle haemorrhage* | 100 | 114.08 (92.99, 139.95) | 112.6 (112.4, 112.81) | 10295.96 | 104.87 (88.38) | 6.71 (6.41) |
| Cerebral haemorrhage* | 95 | 10.32 (8.42, 12.64) | 10.2 (10, 10.4) | 783.98 | 10.14 (8.55) | 3.34 (3.04) |
| Melaena* | 91 | 12.36 (10.04, 15.21) | 12.23 (12.02, 12.43) | 931.4 | 12.14 (10.2) | 3.6 (3.3) |
| Abdominal pain* | 86 | 3.25 (2.62, 4.02) | 3.22 (3.01, 3.43) | 131.95 | 3.22 (2.69) | 1.69 (1.37) |
| Shock haemorrhagic* | 86 | 36.69 (29.59, 45.49) | 36.29 (36.07, 36.5) | 2882.86 | 35.46 (29.62) | 5.15 (4.83) |
| Gastrointestinal haemorrhage* | 69 | 2.78 (2.2, 3.53) | 2.77 (2.53, 3) | 78.01 | 2.76 (2.27) | 1.47 (1.12) |
| Oedema peripheral* | 66 | 2.72 (2.14, 3.47) | 2.71 (2.47, 2.95) | 71.28 | 2.71 (2.21) | 1.44 (1.08) |
| Dyspnoea | 63 | 0.66 (0.52, 0.85) | 0.67 (0.42, 0.91) | 10.57 | 0.67 (0.54) | -0.58 (-0.95) |
| Renal failure* | 62 | 2.73 (2.12, 3.5) | 2.71 (2.46, 2.96) | 67.06 | 2.71 (2.2) | 1.44 (1.07) |
| Post procedural haemorrhage* | 62 | 35.28 (27.4, 45.42) | 35 (34.75, 35.25) | 2001.82 | 34.23 (27.7) | 5.1 (4.73) |
| Thrombocytopenia* | 60 | 3 (2.33, 3.87) | 2.98 (2.73, 3.24) | 79.23 | 2.98 (2.41) | 1.58 (1.2) |
| Fall | 59 | 0.74 (0.57, 0.96) | 0.74 (0.49, 1) | 5.28 | 0.74 (0.6) | -0.43 (-0.8) |
| Haematemesis* | 59 | 10.87 (8.41, 14.06) | 10.8 (10.54, 11.05) | 521.2 | 10.73 (8.65) | 3.42 (3.05) |
| Pain in extremity* | 58 | 1.33 (1.03, 1.73) | 1.33 (1.07, 1.59) | 4.78 | 1.33 (1.07) | 0.41 (0.03) |
| Hypotension* | 57 | 1.48 (1.14, 1.92) | 1.48 (1.22, 1.74) | 8.89 | 1.48 (1.19) | 0.56 (0.18) |
| Haematuria* | 54 | 6.23 (4.77, 8.15) | 6.2 (5.93, 6.46) | 234.65 | 6.18 (4.93) | 2.63 (2.24) |
| Subdural haematoma* | 54 | 10.72 (8.19, 14.02) | 10.65 (10.38, 10.92) | 469.09 | 10.58 (8.45) | 3.4 (3.01) |
| Vomiting | 52 | 0.93 (0.71, 1.23) | 0.93 (0.66, 1.21) | 0.24 | 0.93 (0.74) | -0.1 (-0.5) |
| Pallor* | 43 | 11.64 (8.62, 15.73) | 11.58 (11.28, 11.88) | 412.74 | 11.5 (8.94) | 3.52 (3.09) |
| General physical health deterioration* | 43 | 2.04 (1.51, 2.75) | 2.03 (1.73, 2.33) | 22.57 | 2.03 (1.58) | 1.02 (0.59) |
| Retroperitoneal haematoma* | 42 | 64.12 (47.05, 87.38) | 63.77 (63.46, 64.08) | 2490.23 | 61.23 (47.26) | 5.94 (5.49) |
| Red blood cell count decreased* | 42 | 7.58 (5.59, 10.27) | 7.54 (7.24, 7.84) | 237.31 | 7.51 (5.82) | 2.91 (2.47) |
| Malaise | 40 | 0.65 (0.48, 0.89) | 0.66 (0.35, 0.96) | 7.32 | 0.66 (0.51) | -0.61 (-1.06) |
| Pain | 39 | 0.66 (0.48, 0.91) | 0.67 (0.35, 0.98) | 6.61 | 0.67 (0.51) | -0.59 (-1.04) |
| Coma* | 37 | 6.12 (4.43, 8.46) | 6.1 (5.78, 6.42) | 157.2 | 6.08 (4.64) | 2.6 (2.13) |
| Nausea | 36 | 0.38 (0.28, 0.53) | 0.38 (0.06, 0.71) | 35.85 | 0.38 (0.29) | -1.38 (-1.85) |
| Asthenia | 35 | 0.5 (0.36, 0.7) | 0.5 (0.17, 0.83) | 17.56 | 0.5 (0.38) | -1 (-1.48) |
| Headache | 35 | 0.6 (0.43, 0.83) | 0.6 (0.27, 0.93) | 9.45 | 0.6 (0.45) | -0.74 (-1.22) |
| Confusional state | 35 | 1.06 (0.76, 1.48) | 1.06 (0.73, 1.39) | 0.12 | 1.06 (0.8) | 0.09 (-0.4) |
| Heparin-induced thrombocytopenia* | 35 | 32.88 (23.5, 45.99) | 32.73 (32.4, 33.07) | 1054.06 | 32.06 (24.21) | 5 (4.52) |
| Abdominal wall haematoma* | 34 | 48.31 (34.31, 68.02) | 48.1 (47.76, 48.44) | 1519.85 | 46.65 (35.03) | 5.54 (5.05) |
| Rectal haemorrhage* | 34 | 3.53 (2.52, 4.95) | 3.52 (3.18, 3.85) | 61.24 | 3.51 (2.65) | 1.81 (1.32) |
| Platelet count decreased* | 34 | 1.71 (1.22, 2.39) | 1.7 (1.37, 2.04) | 9.88 | 1.7 (1.28) | 0.77 (0.28) |
| Epistaxis* | 33 | 2.25 (1.6, 3.17) | 2.25 (1.91, 2.59) | 22.89 | 2.25 (1.69) | 1.17 (0.67) |
| Cerebrovascular accident | 32 | 1 (0.71, 1.42) | 1 (0.66, 1.35) | 0 | 1 (0.75) | 0 (-0.5) |
| Post procedural haematoma* | 32 | 83.35 (58.35, 119.06) | 83.01 (82.65, 83.36) | 2457.81 | 78.74 (58.43) | 6.3 (5.78) |
| Shock* | 31 | 8.2 (5.76, 11.68) | 8.17 (7.82, 8.52) | 194.07 | 8.13 (6.05) | 3.02 (2.51) |
| Intra-abdominal haematoma* | 30 | 96.9 (66.95, 140.26) | 96.53 (96.16, 96.9) | 2666.08 | 90.8 (66.63) | 6.5 (5.97) |
| Haematocrit decreased* | 30 | 7.5 (5.23, 10.74) | 7.47 (7.11, 7.83) | 167.46 | 7.44 (5.51) | 2.9 (2.37) |
| Pyrexia | 29 | 0.65 (0.45, 0.93) | 0.65 (0.29, 1.01) | 5.55 | 0.65 (0.48) | -0.62 (-1.15) |
| Cerebral haematoma* | 29 | 25.86 (17.9, 37.36) | 25.77 (25.4, 26.13) | 678.91 | 25.35 (18.64) | 4.66 (4.13) |
| Skin necrosis* | 28 | 34.6 (23.78, 50.36) | 34.48 (34.11, 34.86) | 890.1 | 33.74 (24.64) | 5.08 (4.53) |
| Blood pressure decreased* | 27 | 2.18 (1.49, 3.18) | 2.18 (1.8, 2.55) | 17.19 | 2.18 (1.59) | 1.12 (0.57) |
| Injection site haematoma* | 27 | 26.1 (17.83, 38.2) | 26.01 (25.63, 26.39) | 638.34 | 25.58 (18.6) | 4.68 (4.13) |
| Hemiplegia* | 27 | 16.37 (11.19, 23.93) | 16.31 (15.93, 16.69) | 384.04 | 16.15 (11.75) | 4.01 (3.46) |
| Contusion* | 26 | 1.73 (1.18, 2.55) | 1.73 (1.35, 2.11) | 8 | 1.73 (1.25) | 0.79 (0.23) |

Abbreviation: Asterisks (*) indicate positive signals in algorithm; ROR, reporting odds ratio; PRR, proportional reporting ratio; EBGM, empirical Bayesian geometric mean; EBGM05, the lower limit of the 95% CI of EBGM; IC, information component; IC025, the lower limit of the 95% CI of the IC; χ2, chi-squared; CI, confidence interval; PT, preferred term.

Supplementary Table 11:

Top 50 most frequent AEs for fondaparinux sodium at the PT level reported by healthcare professionals from FAERS data

| PT | Case numbers | ROR(95%CI) | PRR(95%CI) | χ2 | EBGM(EBGM05) | IC(IC025) |
| --- | --- | --- | --- | --- | --- | --- |
| Haematoma* | 297 | 58.61 (52.15-65.87) | 56.71, (56.59-56.82) | 15945.7 | 55.62 (50.44) | 5.8 (5.63) |
| Anaemia* | 290 | 7.32 (6.51-8.23) | 7.12, (7-7.23) | 1527.61 | 7.1 (6.44) | 2.83 (2.66) |
| Haemoglobin decreased* | 163 | 8.47 (7.25-9.89) | 8.33, (8.18-8.49) | 1051.07 | 8.31 (7.3) | 3.06 (2.83) |
| Haemorrhage* | 158 | 10.22 (8.73-11.97) | 10.06, (9.9-10.21) | 1286.79 | 10.03 (8.79) | 3.33 (3.09) |
| Cerebral haemorrhage* | 115 | 15.82 (13.16-19.03) | 15.63, (15.45-15.82) | 1568.04 | 15.55 (13.33) | 3.96 (3.69) |
| Muscle haemorrhage* | 102 | 127.76 (104.66-155.95) | 126.32, (126.12-126.51) | 12144.55 | 121 (102.41) | 6.92 (6.63) |
| Melaena* | 100 | 18.39 (15.09-22.41) | 18.2, (18-18.39) | 1615.98 | 18.09 (15.33) | 4.18 (3.89) |
| Shock haemorrhagic* | 94 | 49.6 (40.41-60.89) | 49.09, (48.89-49.3) | 4354.86 | 48.28 (40.67) | 5.59 (5.29) |
| Abdominal pain* | 87 | 2.29 (1.85-2.82) | 2.27, (2.07-2.48) | 62.34 | 2.27 (1.9) | 1.18 (0.88) |
| Thrombocytopenia* | 86 | 3.01 (2.43-3.72) | 2.99, (2.78-3.2) | 114.03 | 2.99 (2.5) | 1.58 (1.27) |
| Post procedural haemorrhage* | 86 | 46.03 (37.16-57.02) | 45.6, (45.39-45.81) | 3693.35 | 44.9 (37.54) | 5.49 (5.18) |
| Dyspnoea | 83 | 0.97 (0.78-1.21) | 0.97, (0.76-1.19) | 0.06 | 0.97 (0.81) | -0.04 (-0.35) |
| Gastrointestinal haemorrhage* | 78 | 5 (4-6.25) | 4.97, (4.75-5.19) | 247.07 | 4.96 (4.11) | 2.31 (1.98) |
| Haematemesis* | 71 | 14.33 (11.34-18.11) | 14.23, (13.99-14.46) | 869.27 | 14.16 (11.64) | 3.82 (3.48) |
| Pain in extremity* | 68 | 1.87 (1.47-2.37) | 1.86, (1.62-2.1) | 27.12 | 1.86 (1.52) | 0.89 (0.55) |
| Vomiting | 67 | 0.93 (0.74-1.19) | 0.94, (0.7-1.17) | 0.3 | 0.94 (0.76) | -0.1 (-0.45) |
| Heparin-induced thrombocytopenia* | 61 | 46.62 (36.17-60.1) | 46.31, (46.06-46.57) | 2661.67 | 45.59 (36.87) | 5.51 (5.14) |
| Subdural haematoma* | 59 | 17.8 (13.77-23.01) | 17.69, (17.43-17.94) | 923.47 | 17.58 (14.18) | 4.14 (3.76) |
| Pyrexia | 59 | 0.91 (0.7-1.17) | 0.91, (0.65-1.16) | 0.58 | 0.91 (0.73) | -0.14 (-0.52) |
| Oedema peripheral* | 59 | 2.71 (2.09-3.49) | 2.69, (2.44-2.95) | 62.94 | 2.69 (2.17) | 1.43 (1.05) |
| Hypotension* | 55 | 1.36 (1.04-1.77) | 1.36, (1.09-1.62) | 5.15 | 1.36 (1.09) | 0.44 (0.05) |
| Haematuria* | 54 | 7.5 (5.74-9.8) | 7.46, (7.19-7.73) | 301.53 | 7.44 (5.95) | 2.9 (2.51) |
| Nausea | 53 | 0.49 (0.37-0.64) | 0.49, (0.22-0.76) | 28.65 | 0.49 (0.39) | -1.03 (-1.43) |
| Fall | 50 | 1.09 (0.83-1.44) | 1.09, (0.82-1.37) | 0.4 | 1.09 (0.87) | 0.13 (-0.28) |
| Post procedural haematoma* | 49 | 150.83 (113.08-201.17) | 150.01, (149.72-150.3) | 6890.8 | 142.57 (112.04) | 7.16 (6.74) |
| Headache | 44 | 0.57 (0.42-0.76) | 0.57, (0.28-0.87) | 14.32 | 0.57 (0.45) | -0.81 (-1.24) |
| Renal failure* | 43 | 2.04 (1.51-2.76) | 2.04, (1.74-2.34) | 22.76 | 2.04 (1.59) | 1.03 (0.59) |
| Retroperitoneal haematoma* | 42 | 80.58 (59.26-109.58) | 80.21, (79.91-80.52) | 3195.78 | 78.05 (60.35) | 6.29 (5.84) |
| Haematocrit decreased* | 42 | 9.56 (7.05-12.95) | 9.52, (9.21-9.82) | 319.22 | 9.49 (7.36) | 3.25 (2.8) |
| Coma* | 41 | 3.94 (2.9-5.35) | 3.92, (3.62-4.23) | 89.3 | 3.92 (3.03) | 1.97 (1.52) |
| Malaise | 41 | 0.77 (0.56-1.04) | 0.77, (0.46-1.07) | 2.93 | 0.77 (0.59) | -0.38 (-0.83) |
| Red blood cell count decreased* | 40 | 8.55 (6.27-11.68) | 8.52, (8.21-8.83) | 264.88 | 8.5 (6.55) | 3.09 (2.63) |
| Platelet count decreased* | 39 | 1.92 (1.4-2.62) | 1.91, (1.6-2.23) | 16.99 | 1.91 (1.47) | 0.93 (0.48) |
| Abdominal wall haematoma* | 38 | 82.61 (59.8-114.13) | 82.26, (81.94-82.59) | 2965.15 | 79.99 (61.03) | 6.32 (5.85) |
| Hepatic function abnormal* | 38 | 4.25 (3.09-5.84) | 4.23, (3.91-4.55) | 93.74 | 4.23 (3.24) | 2.08 (1.62) |
| Asthenia | 37 | 0.72 (0.52-1) | 0.72, (0.4-1.04) | 3.96 | 0.72 (0.55) | -0.47 (-0.94) |
| Intra-abdominal haematoma* | 37 | 148.26 (106.47-206.45) | 147.65, (147.32-147.98) | 5124.31 | 140.44 (106.45) | 7.13 (6.65) |
| Pallor* | 36 | 6.52 (4.7-9.05) | 6.5, (6.17-6.82) | 167.17 | 6.48 (4.93) | 2.7 (2.22) |
| Pain | 35 | 0.55 (0.4-0.77) | 0.55, (0.22-0.89) | 12.58 | 0.56 (0.42) | -0.85 (-1.33) |
| Shock* | 35 | 6.53 (4.69-9.11) | 6.51, (6.18-6.84) | 163.04 | 6.5 (4.92) | 2.7 (2.22) |
| Rectal haemorrhage* | 34 | 4.87 (3.48-6.82) | 4.85, (4.52-5.19) | 103.93 | 4.85 (3.66) | 2.28 (1.79) |
| Blood pressure decreased* | 32 | 3.03 (2.14-4.29) | 3.02, (2.67-3.37) | 43.27 | 3.02 (2.26) | 1.59 (1.09) |
| Dizziness | 32 | 0.53 (0.37-0.75) | 0.53, (0.18-0.88) | 13.37 | 0.53 (0.4) | -0.91 (-1.42) |
| Condition aggravated | 32 | 0.64 (0.45-0.9) | 0.64, (0.29-0.99) | 6.53 | 0.64 (0.48) | -0.64 (-1.15) |
| Chest pain | 31 | 1.11 (0.78-1.57) | 1.11, (0.75-1.46) | 0.32 | 1.11 (0.82) | 0.15 (-0.37) |
| Epistaxis* | 31 | 2.8 (1.97-3.98) | 2.79, (2.44-3.14) | 35.62 | 2.79 (2.08) | 1.48 (0.97) |
| General physical health deterioration | 29 | 1.39 (0.97-2.01) | 1.39, (1.03-1.76) | 3.22 | 1.39 (1.03) | 0.48 (-0.05) |
| Skin necrosis* | 29 | 22.89 (15.87-33.01) | 22.82, (22.45-23.18) | 600.33 | 22.65 (16.67) | 4.5 (3.97) |
| Platelet count increased* | 28 | 12.32 (8.5-17.87) | 12.29, (11.91-12.66) | 289.1 | 12.24 (8.97) | 3.61 (3.08) |
| Confusional state | 28 | 1.02 (0.7-1.48) | 1.02, (0.65-1.39) | 0.01 | 1.02 (0.75) | 0.03 (-0.51) |

Abbreviation: Asterisks (*) indicate positive signals in algorithm; ROR, reporting odds ratio; PRR, proportional reporting ratio; EBGM, empirical Bayesian geometric mean; EBGM05, the lower limit of the 95% CI of EBGM; IC, information component; IC025, the lower limit of the 95% CI of the IC; χ2, chi-squared; CI, confidence interval; PT, preferred term.

Supplementary Table 12:

Top 50 most frequent AEs for fondaparinux sodium at the PT level reported by consumers from FAERS data.

| PT | Case numbers | ROR(95%CI) | PRR(95%CI) | χ2 | EBGM(EBGM05) | IC(IC025) |
| --- | --- | --- | --- | --- | --- | --- |
| Haematoma* | 261 | 116.39 (102.66-131.97) | 112.42, (112.3-112.54) | 27824.29 | 108.53 (97.7) | 6.76 (6.58) |
| Anaemia* | 192 | 13.22 (11.45-15.26) | 12.91, (12.77-13.05) | 2105.2 | 12.86 (11.41) | 3.69 (3.47) |
| Haemorrhage* | 163 | 12.29 (10.52-14.36) | 12.05, (11.9-12.2) | 1648.22 | 12.01 (10.54) | 3.59 (3.36) |
| Haemoglobin decreased* | 104 | 10.35 (8.53-12.57) | 10.23, (10.03-10.42) | 863.94 | 10.2 (8.67) | 3.35 (3.07) |
| Muscle haemorrhage* | 92 | 338.23 (272.46-419.88) | 334.14, (333.92-334.35) | 27592.51 | 301.81 (251.86) | 8.24 (7.92) |
| Injection site pain* | 69 | 1.36 (1.08-1.73) | 1.36, (1.13-1.6) | 6.67 | 1.36 (1.12) | 0.45 (0.1) |
| Shock haemorrhagic* | 68 | 180.77 (141.42-231.07) | 179.16, (178.91-179.4) | 11391.33 | 169.45 (137.99) | 7.4 (7.05) |
| Pain in extremity* | 65 | 1.36 (1.07-1.74) | 1.36, (1.12-1.6) | 6.21 | 1.36 (1.11) | 0.44 (0.09) |
| Oedema peripheral* | 61 | 4.68 (3.63-6.02) | 4.65, (4.4-4.9) | 174.64 | 4.64 (3.76) | 2.21 (1.85) |
| Cerebral haemorrhage* | 58 | 19.94 (15.39-25.84) | 19.8, (19.54-20.05) | 1029.05 | 19.68 (15.84) | 4.3 (3.92) |
| Headache | 56 | 0.57 (0.44-0.74) | 0.57, (0.31-0.83) | 18.31 | 0.57 (0.46) | -0.81 (-1.19) |
| Pain | 55 | 0.62 (0.48-0.81) | 0.63, (0.36-0.89) | 12.33 | 0.63 (0.5) | -0.67 (-1.06) |
| Thrombocytopenia* | 55 | 14.99 (11.49-19.56) | 14.89, (14.63-15.15) | 709.57 | 14.82 (11.87) | 3.89 (3.5) |
| Contusion* | 55 | 3.62 (2.78-4.72) | 3.6, (3.34-3.86) | 103.39 | 3.6 (2.88) | 1.85 (1.46) |
| Hypotension* | 53 | 3.17 (2.42-4.16) | 3.16, (2.89-3.43) | 78.19 | 3.15 (2.52) | 1.66 (1.26) |
| Dyspnoea | 52 | 0.7 (0.53-0.92) | 0.7, (0.43-0.97) | 6.73 | 0.7 (0.56) | -0.51 (-0.91) |
| Malaise | 52 | 0.71 (0.54-0.93) | 0.71, (0.44-0.98) | 6.05 | 0.71 (0.57) | -0.49 (-0.89) |
| Melaena* | 48 | 45.05 (33.85-59.96) | 44.77, (44.49-45.06) | 2025.3 | 44.15 (34.76) | 5.46 (5.05) |
| Pyrexia* | 48 | 1.39 (1.05-1.85) | 1.39, (1.11-1.67) | 5.25 | 1.39 (1.1) | 0.47 (0.06) |
| Injection site haematoma* | 47 | 16.27 (12.21-21.69) | 16.18, (15.89-16.46) | 665.98 | 16.1 (12.66) | 4.01 (3.59) |
| Renal failure* | 46 | 4.42 (3.31-5.9) | 4.4, (4.11-4.68) | 120.7 | 4.39 (3.45) | 2.13 (1.71) |
| Abdominal pain* | 46 | 1.73 (1.29-2.31) | 1.72, (1.43-2.01) | 13.97 | 1.72 (1.35) | 0.78 (0.36) |
| Fall | 44 | 0.92 (0.68-1.23) | 0.92, (0.62-1.21) | 0.34 | 0.92 (0.71) | -0.13 (-0.56) |
| Nausea | 42 | 0.37 (0.28-0.51) | 0.38, (0.08-0.68) | 43.85 | 0.38 (0.29) | -1.41 (-1.85) |
| Heparin-induced thrombocytopenia* | 42 | 717.5 (512.68-1004.13) | 713.52, (713.19-713.86) | 24305.9 | 580.52 (438.21) | 9.18 (8.7) |
| Vomiting | 42 | 0.72 (0.53-0.98) | 0.72, (0.42-1.02) | 4.51 | 0.72 (0.56) | -0.47 (-0.91) |
| Epistaxis* | 41 | 3.99 (2.93-5.42) | 3.97, (3.67-4.28) | 91.16 | 3.97 (3.07) | 1.99 (1.54) |
| Platelet count decreased* | 39 | 3.74 (2.73-5.13) | 3.73, (3.41-4.04) | 77.87 | 3.72 (2.86) | 1.9 (1.44) |
| Cerebrovascular accident* | 38 | 1.68 (1.22-2.31) | 1.68, (1.36-1.99) | 10.39 | 1.68 (1.28) | 0.74 (0.28) |
| International normalised ratio increased* | 36 | 24.65 (17.74-34.24) | 24.53, (24.21-24.86) | 806.46 | 24.35 (18.49) | 4.61 (4.13) |
| Confusional state* | 35 | 1.92 (1.37-2.67) | 1.91, (1.58-2.24) | 15.24 | 1.91 (1.45) | 0.93 (0.45) |
| Subdural haematoma* | 35 | 38.35 (27.46-53.57) | 38.18, (37.85-38.51) | 1251.99 | 37.73 (28.53) | 5.24 (4.75) |
| Asthenia | 34 | 0.63 (0.45-0.88) | 0.63, (0.3-0.97) | 7.26 | 0.63 (0.48) | -0.66 (-1.15) |
| Pallor* | 34 | 14.48 (10.33-20.3) | 14.42, (14.08-14.76) | 422.83 | 14.36 (10.82) | 3.84 (3.35) |
| Gastrointestinal haemorrhage* | 33 | 3.46 (2.46-4.88) | 3.45, (3.11-3.79) | 57.48 | 3.45 (2.59) | 1.79 (1.29) |
| Pruritus | 33 | 0.67 (0.48-0.95) | 0.67, (0.33-1.01) | 5.26 | 0.67 (0.51) | -0.57 (-1.07) |
| General physical health deterioration* | 33 | 3.36 (2.39-4.74) | 3.35, (3.01-3.69) | 54.52 | 3.35 (2.52) | 1.74 (1.25) |
| Haematemesis* | 32 | 14.22 (10.04-20.14) | 14.17, (13.82-14.51) | 389.9 | 14.11 (10.54) | 3.82 (3.31) |
| Haematuria* | 30 | 10.53 (7.35-15.08) | 10.49, (10.13-10.85) | 256.86 | 10.46 (7.75) | 3.39 (2.87) |
| Abdominal wall haematoma* | 29 | 263.86 (180.5-385.73) | 262.85, (262.48-263.23) | 6975.24 | 242.44 (176.45) | 7.92 (7.37) |
| Urticaria* | 29 | 1.47 (1.02-2.12) | 1.47, (1.11-1.83) | 4.36 | 1.47 (1.08) | 0.56 (0.03) |
| Chest pain | 28 | 1.2 (0.83-1.74) | 1.2, (0.83-1.57) | 0.93 | 1.2 (0.88) | 0.26 (-0.27) |
| Retroperitoneal haematoma* | 27 | 278.02 (187.43-412.4) | 277.03, (276.64-277.43) | 6818.49 | 254.45 (182.94) | 7.99 (7.42) |
| Dizziness | 27 | 0.35 (0.24-0.5) | 0.35, (-0.03-0.72) | 33.34 | 0.35 (0.25) | -1.52 (-2.07) |
| Rash | 26 | 0.49 (0.33-0.72) | 0.49, (0.1-0.87) | 14.02 | 0.49 (0.35) | -1.03 (-1.59) |
| Red blood cell count decreased* | 26 | 7.28 (4.95-10.71) | 7.26, (6.88-7.65) | 140.14 | 7.25 (5.25) | 2.86 (2.3) |
| Coma* | 25 | 7.85 (5.3-11.64) | 7.83, (7.44-8.22) | 148.66 | 7.81 (5.62) | 2.97 (2.4) |
| Rectal haemorrhage* | 25 | 4.86 (3.28-7.19) | 4.84, (4.45-5.23) | 76.16 | 4.84 (3.48) | 2.27 (1.71) |
| Post procedural haemorrhage* | 25 | 16.74 (11.29-24.82) | 16.69, (16.3-17.08) | 366.84 | 16.61 (11.95) | 4.05 (3.49) |
| Erythema | 25 | 0.78 (0.53-1.16) | 0.79, (0.39-1.18) | 1.47 | 0.79 (0.57) | -0.35 (-0.92) |

Abbreviation: Asterisks (*) indicate positive signals in algorithm; ROR, reporting odds ratio; PRR, proportional reporting ratio; EBGM, empirical Bayesian geometric mean; EBGM05, the lower limit of the 95% CI of EBGM; IC, information component; IC025, the lower limit of the 95% CI of the IC; χ2, chi-squared; CI, confidence interval; PT, preferred term.

Supplementary Table 13

Top 50 most frequent adverse events for fondaparinux sodium excluding common medication co-usage at the PT level from FAERS data

| PT | Case numbers | ROR(95%CI) | PRR(95%CI) | χ2 | EBGM(EBGM05) | IC(IC025) |
| --- | --- | --- | --- | --- | --- | --- |
| Haematoma* | 516 | 77.14 (70.59, 84.29) | 74.65 (74.56, 74.73) | 36697.15 | 73.05 (67.83) | 6.19 (6.06) |
| Anaemia* | 449 | 9.05 (8.24, 9.94) | 8.82 (8.73, 8.91) | 3113.9 | 8.8 (8.13) | 3.14 (3) |
| Haemorrhage* | 316 | 11.91 (10.65, 13.31) | 11.69 (11.58, 11.8) | 3082.66 | 11.65 (10.61) | 3.54 (3.38) |
| Haemoglobin decreased* | 265 | 9.74 (8.62, 11) | 9.59 (9.47, 9.71) | 2036.72 | 9.57 (8.64) | 3.26 (3.08) |
| Muscle haemorrhage* | 171 | 173.77 (148.9, 202.79) | 171.9 (171.74, 172.05) | 27645.27 | 163.6 (143.77) | 7.35 (7.13) |
| Cerebral haemorrhage* | 162 | 17.24 (14.77, 20.14) | 17.08 (16.92, 17.23) | 2441.22 | 17 (14.93) | 4.09 (3.86) |
| Shock haemorrhagic* | 130 | 64.19 (53.93, 76.41) | 63.67 (63.5, 63.84) | 7871.46 | 62.51 (54.03) | 5.97 (5.71) |
| Melaena* | 130 | 22.19 (18.67, 26.39) | 22.02 (21.85, 22.19) | 2592.54 | 21.88 (18.93) | 4.45 (4.2) |
| Thrombocytopenia* | 129 | 4.46 (3.75, 5.3) | 4.43 (4.26, 4.6) | 342.51 | 4.42 (3.83) | 2.15 (1.89) |
| Dyspnoea | 128 | 0.86 (0.72, 1.02) | 0.86 (0.69, 1.03) | 2.99 | 0.86 (0.74) | -0.22 (-0.47) |
| Abdominal pain* | 127 | 2.1 (1.76, 2.5) | 2.09 (1.91, 2.26) | 72.15 | 2.09 (1.8) | 1.06 (0.8) |
| Pain in extremity* | 122 | 1.53 (1.28, 1.83) | 1.53 (1.35, 1.71) | 22.56 | 1.53 (1.32) | 0.61 (0.35) |
| Gastrointestinal haemorrhage* | 113 | 4.92 (4.09, 5.92) | 4.89 (4.71, 5.08) | 350.08 | 4.89 (4.19) | 2.29 (2.02) |
| Post procedural haemorrhage* | 113 | 36.55 (30.34, 44.02) | 36.29 (36.11, 36.48) | 3837.84 | 35.92 (30.74) | 5.17 (4.89) |
| Oedema peripheral* | 113 | 3.41 (2.84, 4.11) | 3.39 (3.21, 3.58) | 191.06 | 3.39 (2.91) | 1.76 (1.49) |
| Heparin-induced thrombocytopenia* | 104 | 80.57 (66.29, 97.93) | 80.05 (79.86, 80.24) | 7930.84 | 78.22 (66.44) | 6.29 (6) |
| Vomiting | 102 | 0.84 (0.69, 1.02) | 0.84 (0.64, 1.03) | 3.22 | 0.84 (0.71) | -0.26 (-0.54) |
| Haematemesis* | 100 | 14.8 (12.16, 18.03) | 14.72 (14.52, 14.91) | 1273.4 | 14.66 (12.43) | 3.87 (3.58) |
| Hypotension* | 97 | 1.84 (1.5, 2.24) | 1.83 (1.63, 2.03) | 36.72 | 1.83 (1.55) | 0.87 (0.58) |
| Pyrexia | 97 | 1.06 (0.86, 1.29) | 1.05 (0.86, 1.25) | 0.28 | 1.05 (0.89) | 0.08 (-0.22) |
| Headache | 96 | 0.58 (0.47, 0.71) | 0.58 (0.38, 0.78) | 29.55 | 0.58 (0.49) | -0.79 (-1.08) |
| Nausea | 92 | 0.44 (0.36, 0.54) | 0.45 (0.24, 0.65) | 64.24 | 0.45 (0.38) | -1.17 (-1.47) |
| Fall | 92 | 1.05 (0.85, 1.29) | 1.05 (0.85, 1.25) | 0.21 | 1.05 (0.88) | 0.07 (-0.23) |
| Subdural haematoma* | 92 | 24.03 (19.56, 29.51) | 23.89 (23.69, 24.1) | 2004.19 | 23.73 (19.98) | 4.57 (4.27) |
| Malaise | 91 | 0.77 (0.63, 0.95) | 0.77 (0.57, 0.98) | 6.17 | 0.77 (0.65) | -0.37 (-0.67) |
| Pain | 88 | 0.53 (0.43, 0.65) | 0.53 (0.32, 0.74) | 36.33 | 0.53 (0.45) | -0.91 (-1.21) |
| Injection site pain | 85 | 1.13 (0.92, 1.4) | 1.13 (0.92, 1.35) | 1.34 | 1.13 (0.95) | 0.18 (-0.13) |
| Haematuria* | 80 | 8.66 (6.95, 10.79) | 8.62 (8.4, 8.84) | 537.88 | 8.6 (7.16) | 3.1 (2.78) |
| Contusion* | 80 | 3.18 (2.55, 3.96) | 3.17 (2.95, 3.39) | 119 | 3.17 (2.64) | 1.66 (1.34) |
| Renal failure* | 78 | 2.12 (1.7, 2.65) | 2.12 (1.9, 2.34) | 46.13 | 2.12 (1.76) | 1.08 (0.76) |
| Platelet count decreased* | 77 | 2.74 (2.19, 3.43) | 2.73 (2.51, 2.95) | 84.49 | 2.73 (2.26) | 1.45 (1.12) |
| Epistaxis* | 69 | 3.48 (2.75, 4.41) | 3.47 (3.24, 3.71) | 121.46 | 3.47 (2.85) | 1.79 (1.45) |
| Cerebrovascular accident* | 66 | 1.44 (1.13, 1.83) | 1.43 (1.19, 1.68) | 8.71 | 1.43 (1.17) | 0.52 (0.17) |
| Injection site haematoma* | 65 | 15.71 (12.3, 20.05) | 15.64 (15.4, 15.89) | 887.17 | 15.58 (12.7) | 3.96 (3.6) |
| Coma* | 64 | 5.08 (3.97, 6.49) | 5.06 (4.82, 5.31) | 208.36 | 5.05 (4.12) | 2.34 (1.98) |
| Red blood cell count decreased* | 64 | 8.43 (6.6, 10.79) | 8.4 (8.16, 8.65) | 416.68 | 8.39 (6.83) | 3.07 (2.71) |
| Confusional state* | 63 | 1.47 (1.14, 1.88) | 1.46 (1.22, 1.71) | 9.27 | 1.46 (1.19) | 0.55 (0.19) |
| Pallor* | 63 | 8.65 (6.75, 11.08) | 8.62 (8.37, 8.87) | 423.53 | 8.6 (6.99) | 3.1 (2.74) |
| Asthenia | 63 | 0.63 (0.49, 0.81) | 0.63 (0.39, 0.88) | 13.33 | 0.63 (0.52) | -0.66 (-1.02) |
| Retroperitoneal haematoma* | 62 | 108.98 (84.59, 140.41) | 108.56 (108.31, 108.81) | 6401.28 | 105.2 (85.1) | 6.72 (6.35) |
| General physical health deterioration* | 59 | 2.08 (1.61, 2.68) | 2.07 (1.82, 2.33) | 32.72 | 2.07 (1.67) | 1.05 (0.68) |
| Abdominal wall haematoma* | 57 | 111.52 (85.62, 145.26) | 111.12 (110.85, 111.38) | 6021.91 | 107.6 (86.25) | 6.75 (6.36) |
| Haematocrit decreased* | 57 | 10.36 (7.98, 13.44) | 10.32 (10.06, 10.58) | 478.67 | 10.3 (8.28) | 3.36 (2.98) |
| Chest pain | 56 | 1.13 (0.87, 1.46) | 1.13 (0.86, 1.39) | 0.79 | 1.13 (0.9) | 0.17 (-0.21) |
| Dizziness | 56 | 0.42 (0.33, 0.55) | 0.43 (0.17, 0.69) | 43.54 | 0.43 (0.34) | -1.23 (-1.61) |
| Post procedural haematoma* | 56 | 123.72 (94.72, 161.61) | 123.29 (123.02, 123.55) | 6553.02 | 118.97 (95.14) | 6.89 (6.5) |
| Rectal haemorrhage* | 53 | 4.63 (3.53, 6.06) | 4.62 (4.35, 4.89) | 150.05 | 4.61 (3.68) | 2.21 (1.81) |
| Condition aggravated | 53 | 0.7 (0.53, 0.92) | 0.7 (0.43, 0.97) | 6.81 | 0.7 (0.56) | -0.51 (-0.91) |
| Intra-abdominal haematoma* | 49 | 154.98 (116.34, 206.45) | 154.5 (154.21, 154.78) | 7145.34 | 147.77 (116.25) | 7.21 (6.79) |
| Pruritus | 48 | 0.52 (0.39, 0.69) | 0.52 (0.24, 0.8) | 21.34 | 0.52 (0.41) | -0.94 (-1.35) |

Abbreviation: Asterisks (*) indicate positive signals in algorithm; ROR, reporting odds ratio; PRR, proportional reporting ratio; EBGM, empirical Bayesian geometric mean; EBGM05, the lower limit of the 95% CI of EBGM; IC, information component; IC025, the lower limit of the 95% CI of the IC; χ2, chi-squared; CI, confidence interval; PT, preferred term.

Supplementary Table 14

AIC results for the goodness-of-fit test of each model.

| **Model** | **AIC** |
| --- | --- |
| Log-Logistic | 17700.25214 |
| Log-Normal | 17733.27898 |
| Weibull | 18591.66352 |
| Gompertz | 18772.42728 |
| Gamma | 19312.57362 |
| Exponential | 20923.52316 |

Supplementary Table 15

**The READUS-PV checklist**

| **Section and topic** | **Item #** | **Checklist item** | **Location where item is reported** |
| --- | --- | --- | --- |
| **Title** |  |  |  |
|  | 1a | If disproportionality analyses are a prominent component of the published study, the study should be identified as a “disproportionality analysis”. The type of data and name of the database(s) should be specified. | Detailed in the 'Title' section on line 1-3. |
|  | 1b | Report the name of adverse event(s) and/or drug(s) under study, when applicable. |  |
| **Introduction** |  |  |  |
| Background | 2a | Describe the drug(s) and its utilization, the nature of the adverse event(s) under study and its frequency, and the existing knowledge on the drug-event combination. | Detailed in the ' Introduction' section on line 59 – 85. |
|  | 2b | Specify the rationale for performing the analysis, e.g., as part of routine pharmacovigilance, to investigate an overall safety profile, or to assess a pre-specified hypothesis. | Detailed in the ' Introduction' section on line 86 – 95. |
|  | 2c | Explain why ICSR databases and disproportionality analysis are suitable to fill the knowledge gap. | Detailed in the ' Introduction' section on line 96 – 98. |
| Objectives | 3 | State specific objectives, identifying the adverse event(s), the drug(s), and the reference group, including any pre-specified hypothesis, if applicable. | Detailed in the ' Introduction' section on line 107 – 113. |
| **Methods** |  |  |  |
| Study design | 4a | Identify the study (i.e., “disproportionality analysis”) and the type of data used (e.g., “individual case safety reports”). | Detailed in the ' Methods-2.1 Research methodology and data origins ' section on line 115 – 128. |
|  | 4b | Provide an outline of the entire study design, including primary and sensitivity analyses performed, and other designs such as case-by-case analysis or literature review. | Detailed in the ' Figure.1' section on line 129. |
| Data description, access, and pre-processing | 5a | Specify the name of the database(s), the database(s) custodian, and the coverage. Specify the type/number of drugs included within the database and the thesaurus, taxonomies, or ontologies used for coding drugs and events. | Detailed in the ' Methods-2.2. Data extraction' section on line 140 – 148 and line 160 – 171. |
|  | 5b | Specify the extraction dates and describe and justify all choices used for data pre-processing, including any data transformation or exclusion, if appropriate. | Detailed in the ' Methods-2.1 Research methodology and data origins ' section on line 116 – 128. |
| Variables definition | 6a | Describe the study population, including any restriction. | Detailed in the ' Introduction' section on line 106 – 109. |
|  | 6b | Describe the nature and the meaning of key variables assessed in the work. | Detailed in the ' Methods-2.2. Data extraction' section on line 141 – 148 and ' Methods-2.3. Statistical analysis' section on line 177– 181. |
|  | 6c | Specify and justify any grouping of drugs or events. For drugs, specify and justify whether active ingredients/trade names/salts were considered and/or the selected role. | Detailed in the ' Methods-2.2. Data extraction' section on line 153 – 154 and line 170-171. |
|  | 6d | Describe any additional data source used, the type of data, and how they interact with ICSRs. | Detailed in the ' Introduction' section on line 99 –105.  Detailed in the ' Methods-2.2. Data extraction' section on line 162 – 166. |
| Statistical methods | 7a | Present any descriptive analysis performed, specifying variables investigated, statistical tests, and significance thresholds. | Detailed in the ' Methods-2.3 Statistical analysis' section on line 173 – 218. |
|  | 7b | Describe the measure(s) selected for the disproportionality analysis including any threshold used to identify signals of disproportionate reporting. Explain the reason for this choice if applicable. |  |
|  | 7c | Clearly describe any sensitivity analysis and any tool to control confounding, including any restriction, subgroup, stratification, adjustment, or interaction. |  |
|  | 7d | Specify the variables and methods used for the case-by-case analysis, including any algorithm or criteria used to assess causality, if performed. |  |
|  | 7e | Specify any statistical methods used for other data sources. |  |
| **Results** |  |  |  |
| Participants | 8a | Specify the number of individual case safety reports included at each stage, including reasons for exclusion. | Detailed in the ' Results-3.1 Clinical characteristics' section on line 221 –236. |
|  | 8b | Provide key demographic and clinical characteristics of cases, if possible comparing cases with any appropriate reference group. |  |
| Disproportionality analysis | 9 | Present all results including confidence intervals. Present also results of sensitivity analyses, if performed. | Detailed in the ' Results-3.2 Signal detection associated with fondaparinux sodium / 3.3 Distribution of adverse events at the PT level / 3.4 Subgroup analysis / 3.5 Sensitivity analysis / 3.6 The assessment of time to onset for adverse events and Log-logistic distribution analysis' section on line 241 –475. |
| Case-by-case analysis | 10 | Present the case-by-case analysis of key variables. Present the causality assessment, if applicable. | N/A. Aggregated analysis of the entire cohort was provided instead of case-by-case review due to data volume. Detailed in the ' Table 1: Clinical characteristics of fondaparinux sodium adverse event reports from the FAERS (Q1 2004-Q3 2024) FAERS, JADER (Q2 2007 to Q2 2025), and VigiAccess database (Q2 2003 to Q4 2025).’on line 237. |
| **Discussion** |  |  |  |
| Key results | 11 | Discuss key results with reference to study objectives and contextualize them within the current literature and other consulted sources. Clearly discriminate between expected reactions and emerging safety signals. | Detailed in the ' Discussion' section on line 480-529. |
| External validity | 12a | Discuss the external validity of the results to the general population. | Detailed in the ' Discussion' section on line 640-679. |
|  | 12b | Discuss the potential relevance of results in clinical practice |  |
|  | 12c | Propose further study designs if applicable |  |
| Limitations | 13 | Present general limitations, making clear that disproportionality analysis alone cannot prove causation or measure incidence, and specific limitations, including confounding and reporting bias and efforts to mitigate them. | Detailed in the ' Limitations ' section on line 718-748. |
| **Declarations** |  |  |  |
|  | 14a | Provide the source of funding/sponsorship and the role of the funders/sponsors for the present study and for any original study on which the present article is based. | Detailed in the line 769-786. |
|  | 14b | Clearly identify potential commercial and intellectual conflicts of interest (e.g., link to any drug/event investigated, whether financial, legal action, or software used). |  |
|  | 14c | Declare any institutional approval needed or granted in the investigation. |  |
|  | 14d | Include a statement on data availability, code availability (including the version of the statistical software used), and protocol registration. |  |

**The READUS-PV checklist for abstracts**

| **Section and topic** | **Item #** | **Checklist item** | **Location where item is reported** |
| --- | --- | --- | --- |
| Background | 1a | State the aim/rationale for performing the study. | Line 17 - 22 |
|  | 1b | Specify the adverse event(s) and/or the drug(s) under study, when applicable. |  |
|  | 1c | Specify the specific population or setting, when applicable. |  |
| Methods | 2a | Identify the study as a “disproportionality analysis” and specify the type of data used. | Line 23 - 34 |
|  | 2b | Specify the name of the database(s) used and the type of access. |  |
|  | 2c | Specify the timeframe and geographical region, when applicable. |  |
|  | 2d | Specify the disproportionality measure(s) used and their statistical significance threshold(s). |  |
|  | 2e | Specify if a case-by-case analysis is performed. |  |
| Results | 3 | Report main findings including their precision (e.g., 95% confidence intervals), together with a short summary of the case-by-case analysis. | Line 35 - 48 |
| Conclusion | 4a | Clearly report key conclusions. | Line 49 - 54 |
|  | 4b | Acknowledge that the disproportionality analysis is a hypothesis generating or refinement approach. |  |
|  | 4c | State the implications and clinical relevance of the findings. |  |

**Supplementary Figures**


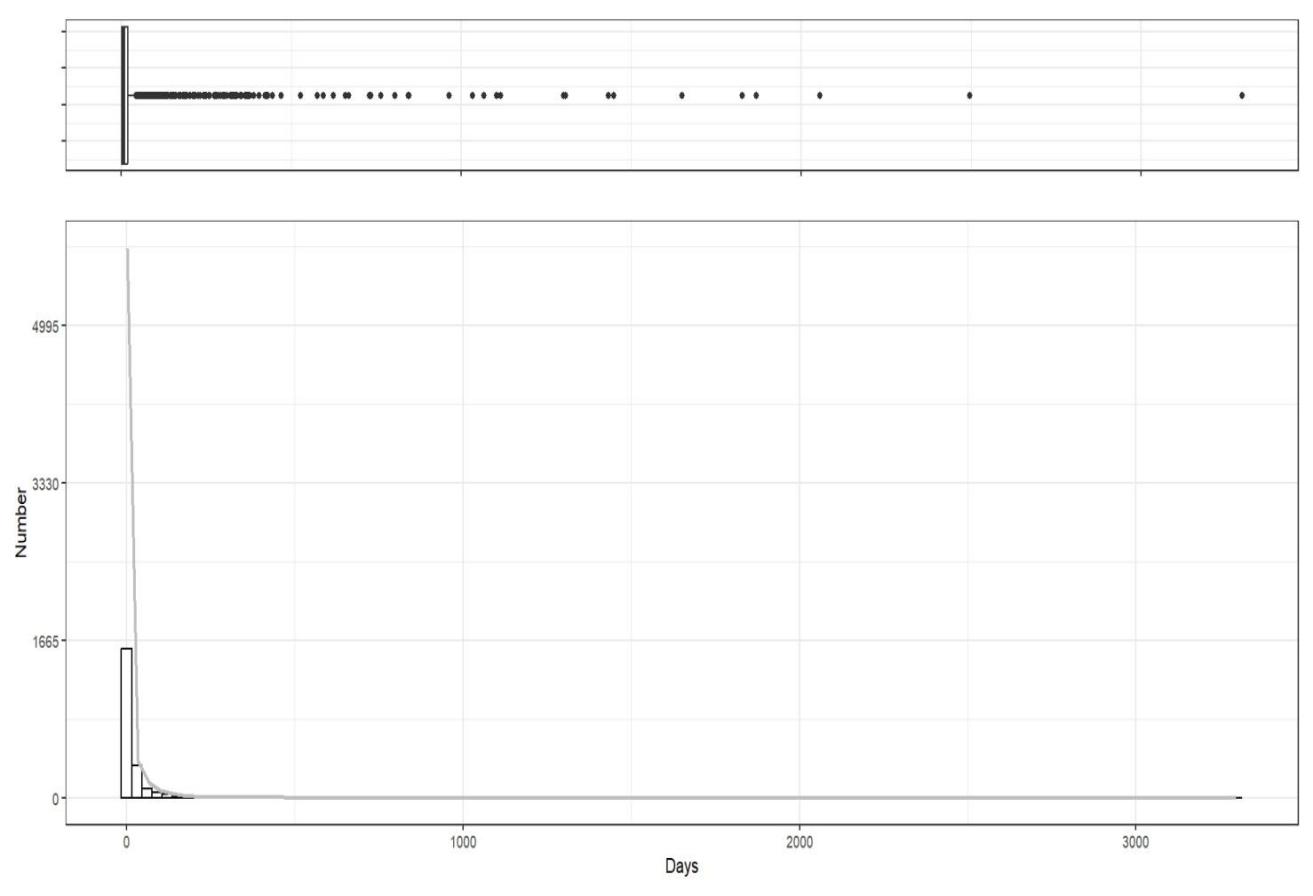


Supplementary Figure 1. Distribution of time-to-onset for fondaparinux sodium-related adverse events. The upper panel shows a box plot illustrating the distribution and outliers of onset time, while the lower panel presents a histogram displaying the number of cases over time. The x-axis shows time (days), and the y-axis represents number of reports.


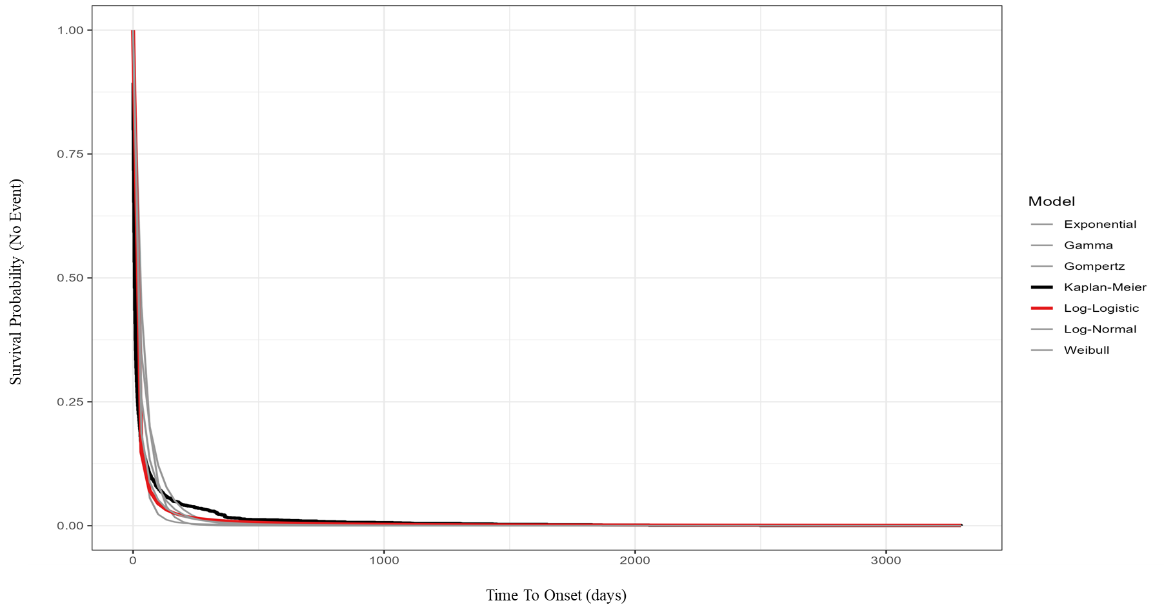


Supplementary Figure 2. Optimal model fitting plots based on the Akaike Information Criterion (AIC).
